# ELBW and ELGAN outcomes in developing nations–Systematic review and meta-analysis

**DOI:** 10.1371/journal.pone.0255352

**Published:** 2021-08-05

**Authors:** Viraraghavan Vadakkencherry Ramaswamy, Thangaraj Abiramalatha, Tapas Bandyopadhyay, Nasreen Banu Shaik, Prathik Bandiya, Debasish Nanda, Abdul Kareem Pullattayil S., Srinivas Murki, Charles Christoph Roehr

**Affiliations:** 1 Department of Neonatology, Ankura Hospital for Women and Children, Hyderabad, India; 2 Department of Neonatology, Sri Ramachandra Institute of Higher Education and Research, Chennai, India; 3 Department of Neonatology, Dr Ram Manohar Lohia Hospital & Post Graduate Institute of Medical Education and Research, New Delhi, India; 4 Department of Neonatology, Indira Gandhi Institute of Child Health, Bengaluru, India; 5 Department of Neonatology, Institute of Medical Sciences and SUM Hospital, Bhubaneswar, Orissa, India; 6 Department of Clinical Library, Sidra Medicine, Ar-Rayyan, Qatar; 7 Department of Neonatology, Paramitha Women and Children’s Hospital, Hyderabad, India; 8 National Perinatal Epidemiology Unit, Nuffield Department of Population Health, Medical Sciences, Division, University of Oxford, Oxford, United Kingdom; 9 Newborn Services, Southmead Hospital, North Bristol Trust, Bristol, United Kingdom; 10 University of Bristol, Women’s and Children Division, Bristol, United Kingdom; Center of Pediatrics, GERMANY

## Abstract

**Context:**

Morbidity and mortality amongst extremely low birth weight (ELBW) and extremely low gestational age neonates (ELGANs) in developing nations has not been well studied.

**Objectives:**

Evaluate survival until discharge, short- and long-term morbidities of ELBW and ELGANs in LMICs.

**Data sources:**

CENTRAL, EMBASE, MEDLINE and Web of Science.

**Study selection:**

Prospective and retrospective observational studies were included.

**Data extraction and synthesis:**

Four authors extracted data independently. Random-effects meta-analysis of proportions was used to synthesize data, modified QUIPS scale to evaluate quality of studies and GRADE approach to ascertain the certainty of evidence (CoE).

**Results:**

192 studies enrolling 22,278 ELBW and 18,338 ELGANs were included. Survival was 34% (95% CI: 31% - 37%) (CoE–low) for ELBW and 39% (34% - 44%) (CoE—moderate) for ELGANs. For ELBW neonates, the survival for low-income (LI), lower middle-income (LMI) and upper middle income (UMI) countries was 18% (11% - 28%), 28% (21% - 35%) and 39% (36% - 42%), respectively. For ELGANs, it was 13% (8% - 20%) for LI, 28% (21% - 36%) for LMI and 48% (42% - 53%) for UMI countries. There was no difference in survival between two epochs: 2000–2009 and 2010–2020. Except for necrotising enterocolitis [ELBW and ELGANs—8% (7% - 10%)] and periventricular leukomalacia [ELBW—7% (4% - 11%); ELGANs—6% (5%-7%)], rates of all other morbidities were higher compared to developed nations. Rates of neurodevelopmental impairment was 17% (7% - 34%) in ELBW neonates and 29% (23% - 37%) in ELGANs.

**Limitations:**

CoE was very low to low for all secondary outcomes.

**Conclusions:**

Mortality and morbidity amongst ELBW and ELGANs is still a significant burden in LMICs. CoE was very low to low for all the secondary outcomes, emphasizing the need for high quality prospective cohort studies.

**Trial registration:**

PROSPERO (CRD42020222873).

## Introduction

Prematurity is one of the leading causes of childhood mortality, rates of which are on the rise across the globe [1–4]. Available data indicate that about 80% of the preterm births happen in the geographical regions of Africa and South Asia [2, 3]. When countries are grouped by their World Bank income categories, it is found that approximately 90% of all preterm births occurs in low‐ and middle‐income countries (LMICs). The average preterm birth rate for LMICs is close to 9–12%, compared to approximately 9% for most of high‐income countries (HICs) [4].

The quantum of impact on decreasing the neonatal mortality rate (NMR) is more pronounced when community based interventions targeting the late preterm and term neonates are implemented successfully across LMICs [1]. It is estimated that about 80% of neonatal deaths can be thwarted in LMICs by achieving a 95% coverage rate of simple neonatal interventions such as neonatal resuscitation, avoiding hypothermia, clean cord practices, kangaroo mother care as well as supporting breast feeding, clubbed with antenatal practices [1]. Henceforth, higher level NICU care targeting extremely low birth weight (ELBW) neonates with a birth weight of less than 1000 grams and extremely low gestational age neonates (ELGANs) born at less than 28 weeks’ gestation might not be a priority as of now in many LMICs.

Every Newborn action plan by WHO envisages reducing national NMR to 12 per 1000 live births by the year 2030 [5]. Historical trends of NMR from developed nations reveal that once a NMR of 15 per 1000 live births is attained, augmenting community based health programmes with facility based neonatal care through establishment of higher level NICUs would facilitate achieving single digit NMR in LMICs [6]. Improved survival rates amongst ELBW and ELGANs in LMICs with such an approach might also be associated with increased rates of typical morbidities of prematurity, including bronchopulmonary dysplasia (BPD), retinopathy of prematurity (ROP) and long term neurodevelopmental impairment (NDI) [7–10]. While previous systematic reviews have tried to quantify the burden of mortality and morbidity in high risk neonates including preterm infants from LMICs, none have been published till date with exclusive focus on the particularly vulnerable group of ELBW neonates and ELGANs [11–13]. Assessing the burden of mortality and morbidity in these infants is vital in developing future newborn targeted health interventions and policies, especially for those LMICs that are transitioning from community based care to a facility based one. Hence, this systematic review and meta-analysis was conducted to study the survival, short- and long-term outcomes of ELGANs and ELBW neonates from LMICs.

## Methods

The protocol for the systematic review was registered with PROSPERO (CRD42020222873) [14]. The reporting of the review is in accordance with PRISMA guidelines [15].

### Literature search

Electronic databases: MEDLINE, EMBASE, Web of Science and Cochrane CENTRAL were searched from 1^st^ January 2000 till 21^st^ November 2020. There were no language restrictions. Google translate (California, USA) was used for translating non-English literature. Studies published in Urdu and Persian were translated with the help of a translator as there were some limitations in Google translate for these languages. Studies published as abstracts were also eligible for inclusion. Only published data was used in this systematic review. Four authors (TA, NBS and PB, DN) conducted the literature search independently in pairs of two using Rayyan-QCRI software [16]. The search strategy for the different databases is provided in S1 Table in S1 File.

### Inclusion criteria

Retrospective as well as prospective observational studies that had reported on outcomes of ELGANs (born at less than 28 weeks’ of gestation) and / or ELBW neonates (birth weight of less than 1000 grams) from a LMIC as per the World Bank country classifications by income levels (2020) were eligible for inclusion [17]. Randomized controlled trials were excluded as certain ELGANs and ELBW neonates who would otherwise have been eligible for inclusion might have been excluded due to a variety of reasons such as stringent inclusion criteria, refusal of consent and presence of co-morbid conditions among others.

### Outcomes

The primary outcome of the review was proportion of neonates who had survived until discharge. Secondary outcomes included prevalence of intraventricular hemorrhage (IVH) (> grade II) [18], periventricular leukomalacia (PVL) (any severity), necrotizing enterocolitis (NEC) (stage II or more) [19], PDA requiring surgical or medical treatment, BPD [oxygen requirement at 36 weeks’ postmenstrual age (PMA)], sepsis (early onset and late onset sepsis) diagnosed based on blood culture as well as on other blood markers, ROP (any ROP, ROP stage ≥ 2 as per ICROP classification [20], ROP requiring laser therapy or intravitreal bevacizumab), extrauterine growth retardation (EUGR) (defined as weight less than 10^th^ centile at 36 weeks’ PMA), any NDI (as defined by authors) and cerebral palsy of any severity, both assessed at 18–24 months’ corrected age.

### Risk of bias (ROB) assessment

The risk of bias assessment was done using a modified QUIPS scale [21]. Four parameters namely, representativeness of the sample, definition and assessment of the outcomes, evaluation of baseline characteristics of the enrolled subjects known to affect the outcomes and method of data collection were evaluated. Studies that had satisfied all the four criteria were classified as having a low risk of bias. Those fulfilling two or three criteria were adjudged as having an intermediate risk of bias and those satisfying one or none were classified as high risk of bias studies. Two authors (TA, NBS) evaluated the risk of bias independently. Disagreements were resolved by consulting a third author (VVR).

### Data collection and synthesis

The data for relevant outcomes were extracted using a pre-specified proforma by two authors independently (PB, DN). Statistical analysis was done using the R-Software. Meta-analysis of proportions was used for synthesis of data. Raw data was used if the proportions were between 0.20 to 0.80. Otherwise, data was logit transformed. Freeman-Tukey Double arcsine transformation of data was preferred over logit transformation when there were many proportions with 0 or 1 values. ‘Metaprop’ package in R-software was used for meta-analysis of proportions. ‘Metaprop’ can analysis meta-analysis of raw data based on proportions as well as convert raw data to logit transformation or Freeman-Tukey Double arcsine transformation and re-transform to proportions by default for meaningful interpretation. A random effects model was chosen as significant heterogeneity was anticipated as reported in prior studies on newborn survival [22]. An inverse variance method was chosen with DerSimonian Liard estimator being used for assessing between study variance [23]. The final estimates were expressed as proportions with 95% confidence interval.

### Certainty of evidence (CoE) assessment

CoE was assessed using a modified GRADE approach [24]. Outcomes from prospective studies started as high quality evidence and other type of studies as low. The parameters of risk of bias, inconsistency, imprecision, indirectness and publication bias were evaluated for downgrading the evidence further. I^2^ value was used to adjudge significant heterogeneity. Imprecision was evaluated based on the point estimate and the 95% confidence interval. If these were to cross a decision threshold to intervene, the CoE was downgraded by one level for imprecision. Though publication bias assessment in meta-analysis of prognosis studies is a contentious topic, we assessed publication bias using funnel plots and Begg’s rank test based on GRADE working group guidelines [24, 25].

### Sensitivity analyses

The following sensitivity analyses were performed for the primary outcome

- Categorizing studies from countries based on three income levels–low income (LI), lower middle-income (LMI) and upper middle-income (UMI).
- Categorizing studies based on geographical regions where they are situated.
- Excluding studies with low sample size (less than 50 subjects).
- Comparison of survival between two time periods—epoch 1: 2000–2009 and epoch 2: 2010–2019.
- Analyzing studies which had evaluated neonates with varying baseline sickness.

## Results

**Fig 1:**
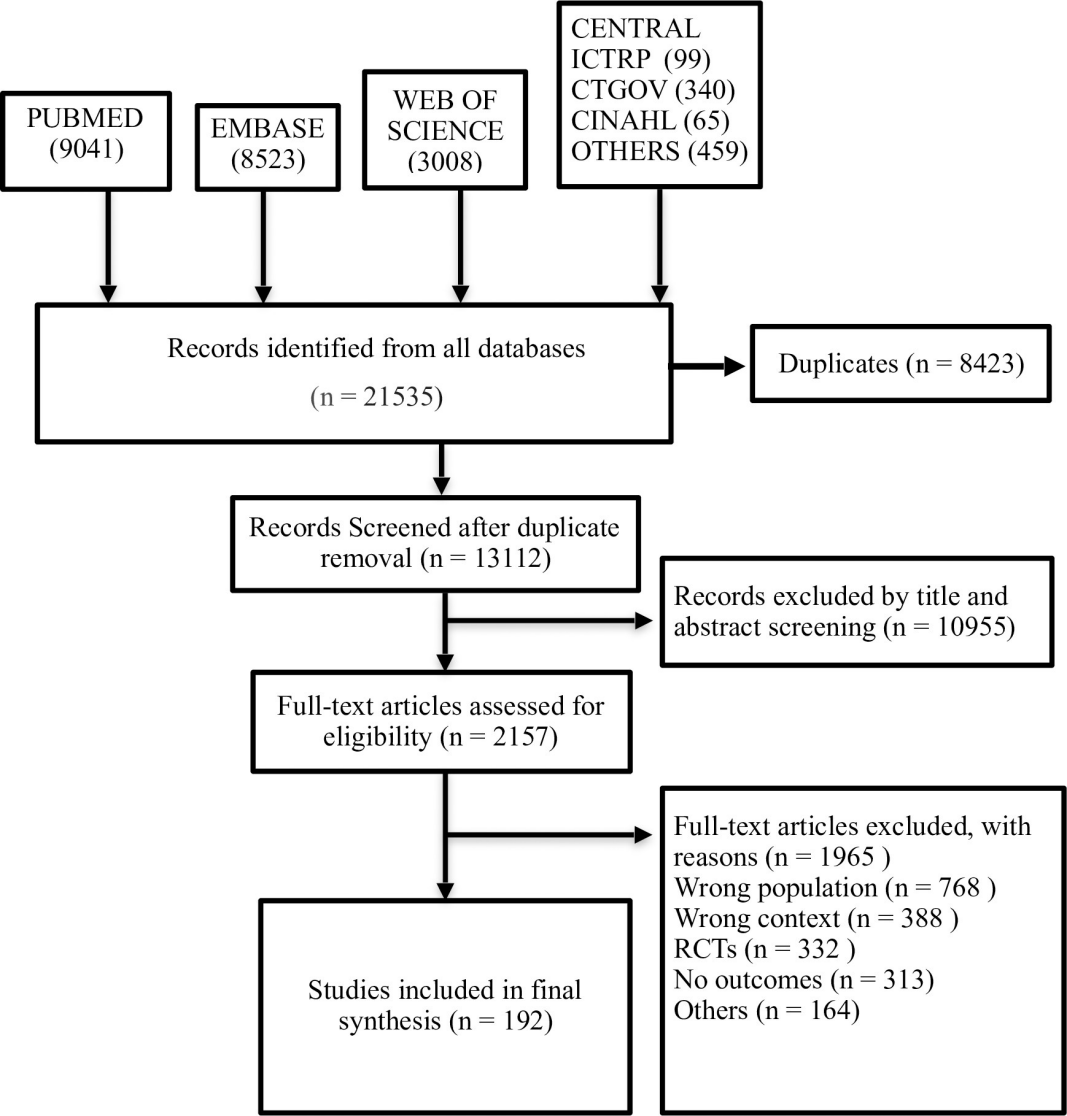
PRISMA flow.

A total of 21,535 studies were identified from the literature search, of which 2157 full texts were assessed for eligibility after removal of duplicates as well as title, abstract screening and 192 studies (ELBW neonates– 22,278; ELGANs– 18,338) were included in the final synthesis (S1 File references 1–192). The PRISMA flow is given in Fig 1. Ninety-two studies with 13,667 ELBW and sixty studies enrolling 8,412 ELGANs had reported on the primary outcome measure of survival until discharge. The included studies were predominantly from middle-income countries. Studies from twenty-four countries situated in six geographical regions namely, Asia, Africa, Europe, Middle East, North America and South America had reported on survival for ELGANs. Similarly, studies from thirty-one countries located in seven different geographical regions (aforementioned regions along with Caribbean) had reported on survival for ELBW neonates. There was a preponderance of studies published from Brazil, China, India, Iran and South Africa. The denominator of the primary outcome measure (live births versus NICU admissions) and level of NICU care were inconsistently reported. Only five studies with 273 ELGANs and ELBW neonates had assessed the long-term neurodevelopmental outcomes at 24 months’ corrected age. The characteristics of included studies is given in Table 1.

**Table 1:** Characteristics of included studies.

AUTHOR/YEAR	REGION	COUNTRY	INCOME CLASSIFICATION	ELGAN / ELBW	PERIOD OF STUDY	SAMPLE SIZE
Abdul-Mumin 2020	Africa	Ghana	LMI	Both	2017	ELGAN-20, ELBW-21
Adegoke 2014	Africa	Nigeria	LMI	Both	2012–2013	ELGAN-15, ELBW-19
Adhikari 2017	Asia	Nepal	LMI	ELBW	2014–2015	38
Adinma 2013	Africa	Nigeria	LMI	ELGAN	2000–2009	2
Afjeh 2013	Middle East	Iran	UMI	Both	2007–2010	ELGAN-196, ELBW-147
Afjeh 2017	Middle East	Iran	UMI	Both	2011–2014	ELGAN-203, ELBW-156
Aggarwal 2002	Asia	India	LMI	ELBW	1999–2000	12
Ahlsen 2015	Africa	Malawi	LI	ELBW	2012	45
Ahmeti 2010	Europe	Kosovo	UMI	ELBW	2002	73
Ali 2019	Asia	Pakistan	LMI	ELBW	2016–2017	200
Ali 2016	Asia	Bangladesh	LMI	ELBW	2013–2014	113
Alizadeh 2015	Middle East	Iran	UMI	Both	2005–2010	ELGAN-32, ELBW-17
Altamemmi 2019	Middle East	Iraq	UMI	ELBW	2003–2008	32
Amadi 2019	Africa	Nigeria	LMI	ELGAN	NA	15
Amadi2015	Africa	Nigeria	LMI	ELBW	2011–2014	22
Andegiorgish 2020	Africa	Eritrea	LI	ELBW	2016	22
Araz-Ersan 2013	Middle East	Turkey	UMI	Both	1996–2010	ELGAN-283, ELBW-410
Arnold 2010	Africa	South Africa	UMI	ELBW	1992–1995	18
Atalay 2013	Middle East	Turkey	UMI	ELBW	2010	ELBW-20
Atasay2003	Middle East	Turkey	UMI	Both	1997–2000	ELGAN-37, ELBW-31
Azeredo Cardoso 2013	South America	Brazil	UMI	ELBW	2002–2006	305
Bajaj 2020	Asia	India	LMI	ELBW	2017–2019	97
Ballot 2010	Africa	South Africa	UMI	ELBW	2006–2007	143
Ballot 2012	Africa	South Africa	UMI	ELBW	2006–2007	95
Ballot 2017	Africa	South Africa	UMI	ELBW	2013–2016	546
Ballot 2017a	Africa	South Africa	UMI	ELBW	2013	34
Bas 2015	Middle East	Turkey	UMI	Both	2011–2013	ELGAN-3737, ELBW-2694
Bas 2018	Middle East	Turkey	UMI	Both	2016–2017	ELGAN-1539, ELBW-1109
Basiri 2015	Middle East	Iran	UMI	Both	2012	ELGAN-68, ELBW-64
Basu 2008	Asia	India	LMI	Both	NA	ELGAN-46, ELBW-45
Bhunwal 2018	Asia	India	LMI	Both	2012–2013	ELGAN-16, ELBW-34
Bokade 2018	Asia	India	LMI	ELBW	2016–2017	47
Bolat 2012	Middle East	Turkey	UMI	ELBW	2009–2011	59
Bonotto 2007	South America	Brazil	UMI	ELGAN	1992–1999	12
Boo 2012	Asia	Malaysia	UMI	Both	2007	ELGAN-1246, ELBW-1051
Braimah 2020	Africa	Ghana	LMI	Both	2018–2019	ELGAN-28, ELBW-20
Buenos Aires 2012	South America	Argentina	UMI	Both	2008–2010	ELGAN-426, ELBW-451
Carneiro 2012	South America	Brazil	UMI	ELBW	2007–2010	57
Castro 2007	South America	Brazil	UMI	ELBW	2002–2003	270
Cauicharagon 2017	North America	Mexico	UMI	ELGAN	2005–2014	2
Cenkcelebi 2014	Mideast	Turkey	UMI	ELBW	2010–2013	235
Cetinkaya 2014	Mideast	Turkey	UMI	ELBW	2011–2013	197
Chaudhari 2009	Asia	India	LMI	Both	2000–2006	ELGAN-58, ELBW-58
Chen 2019	Asia	China	UMI	ELBW	2016–2018	284
Chen 2012	Asia	China	UMI	ELGAN	2005–2006	95
Chen 2015	Asia	China	UMI	ELBW	2010–2012	161
Chiabi 2014	Africa	Cameroon		Both	2003–2011	ELGAN-75, ELBW-74
Chidiebere 2018	Africa	Nigeria	LMI	ELBW	2013–2016	20
Chioukh 2018	Africa	Tunisia	LMI	ELGAN	2012–2013	109
Coyles 2020	Africa	South Africa	UMI	ELBW	2013–2015	104
Dearaujo 2007	South America	Brazil	UMI	Both	1998–2004	ELGAN-30, ELBW-61
Debritoa 2003	South America	Brazil	UMI	Both	1997–2000	ELGAN-70, ELBW-79
Decarvalho 2007	South America	Brazil	UMI	ELGAN	2002–2004	108
Demello 2007	South America	Brazil	UMI	Both	1991–2000	ELGAN-51, ELBW-60
Gebeşçe 2016	Middle East	Turkey	UMI	ELBW	2007–2011	18
Gezmu 2020	Africa	Botswana	UMI	Both	2018–2019	ELGAN-37, ELBW-32
Gharaibeh 2011	Middle East	Jordan	UMI	ELBW	2006–2007	11
Ghaseminejad 2011	Middle East	Iran	UMI	ELBW	2006–2008	7
Golestan 2008	Middle East	Iran	UMI	ELBW	2004	35
Goncalves 2014	South America	Brazil	UMI	ELBW	2009–2011	24
Gooden 2013	Caribbean	Jamaica	UMI	ELBW	2005–2006	46
Gordana 2019	Europe	Serbia	UMI	Both	2006–2011	ELGAN-220, ELBW-157
Goulart 2011	South America	Brazil	UMI	ELBW	1997–2003	36
Gupta 2020	Asia	India	LMI	Both	2017	ELGAN-30, ELBW-49
Hadi 2013	Middle East	Egypt	LMI	ELGAN	2010–2012	15
Haghighi 2013	Middle East	Iran	UMI	ELBW	2010–2011	52
Hakeem 2012	Middle East	Egypt	LMI	ELBW	2009–2010	3
Hendriks 2014	Africa	South Africa	UMI	ELBW	2006–2009	97
Ho 2001	Asia	Malaysia	UMI	Both	1996	ELGAN-168, ELBW-136
Hussain 2012	Middle East	Iraq	UMI	ELGAN	2018–2019	44
Jiang 2020	Asia	China	UMI	ELGAN	2015–2016	320
Jirapaet 2010	Asia	Thailand	UMI	ELBW	1998–2001	5
Jodeiry 2012	Middle East	Iran	UMI	ELGAN	NA	51
Kalimba 2013	Africa	South Africa	UMI	ELBW	2006–2010	382
Karabulut 2019	Middle East	Turkey	UMI	ELGAN	2015–2018	87
Kareem 2011	Middle East	Iraq	UMI	ELBW	2003–2009	48
Karkhaneh 2008	Middle East	Iran	UMI	Both	2003–2007	ELGAN-199, ELBW-117
Kidamba 2018	Africa	South Africa	UMI	Both	2013	ELGAN-15, ELBW-15
Kift 2016	Africa	South Africa	UMI	ELGAN	2009–2014	98
Kirsten 2012	Africa	South Africa	UMI	ELGAN	2007–2009	309
Klingenberg 2003	Africa	Tanzania	LMI	ELBW	1999	11
Koksal 2002	Middle East	Turkey	UMI	ELBW	NA	12
Kong 2016	Asia	China	UMI	ELGAN	2013–2014	148
Kong 2020	Asia	China	UMI	ELGAN	2013–2014	50
Kulali 2019	Middle East	Turkey	UMI	ELGAN	2011–2015	165
Lara-Molina 2013	North America	Mexico	UMI	ELBW	2008–2010	44
Lermann 2008	South America	Brazil	UMI	Both	2002–2004	ELGAN-7, ELBW-28
Li 2018	Asia	China	UMI	ELGAN	2010–2015	79
Li 2019	Asia	China	UMI	ELGAN	2010–2016	307
Lin 2015	Asia	China	UMI	ELBW	2011	258
Liu 2014	Asia	China	UMI	Both	2009–2012	ELGAN-85, ELBW-24
Liu 2005	Asia	China	UMI	ELBW	1996–2000	37
Lomuto 2008	South America	Argentina	UMI	ELBW	2008	190
Luthuli 2019	Africa	South Africa	UMI	Both	2011–2014	ELGAN-53, ELBW-105
Mabhandi 2019	Africa	South Africa	UMI	ELBW	2015–2017	89
Montano Perez 2019	North America	Mexico	UMI	ELBW	2010–2014	52
Martinez-Cruz 2012	North America	Mexico	UMI	ELBW	2000–2008	139
Martinez 2010	North America	Mexico	UMI	ELBW	2005–2006	152
Mcgready 2018	Asia	Thailand	UMI	ELGAN	1995–2015	132
Medina-Valenton 2016	South America	Brazil	UMI	Both	2012–2014	ELGAN-23, ELBW-25
Mekasha 2020	Africa	Ethiopia	LI	ELBW	2016–2018	164
Miles 2017	Asia	Vietnam	LMI	Both	2011–2012	ELGAN-5, ELBW-63
Moghaddam 2015	Middle East	Iran	UMI	ELBW	2010–2014	108
Muhe 2019	Africa	Ethiopia	LI	Both	2016–2018	ELGAN-104, ELBW-165
Mukhopadhyay 2013	Asia	India	LMI	ELBW	2001–2010	436
Nakubulwa 2020	Africa	Uganda	LI	ELBW	2017–2018	18
Navaei 2010	Middle East	Iran	UMI	Both	2005–2006	ELGAN-122, ELBW-107
NEOCOSUR 2002	South America	Multi-Country	UMI	Both	1997–1998	ELGAN-95, ELBW-126
Nepal 2020	Asia	Nepal	LMI	Both	2019	ELGAN-2, ELBW-3
Nevacinovic 2020	Europe	Bosnia	UMI	ELBW	NA	49
NNPD 2004	Asia	India	LMI	ELBW	2000	101
Ntuli 2020	Africa	South Africa	UMI	Both	2015	ELGAN-25, ELBW-50
Ogunlesi 2011	Africa	Nigeria	LMI	ELBW	2008	15
Okello 2019	Africa	Uganda	LI	ELBW	2015–2017	36
Omoigberale 2010	Africa	Benin	LMI	Both	2003–2006	ELGAN-190, ELBW-151
Omer 2014	Africa	Sudan	LI	ELBW	2012–2013	6
Onalo 2015	Africa	Nigeria	LMI	ELBW	2006–2010	42
Onyiriuka 2009	Africa	Nigeria	LMI	ELBW	2000–2003	9
Oomen 2019	Asia	India	LMI	ELBW	2010–2012	30
Osorno-Covarrubiasa 2002	North America	Mexico	UMI	Both	1995–1999	ELGAN-50, ELBW-162
Ozcan 2015	Middle East	Turkey	UMI	ELGAN	2014–2015	18
Pervin 2015	Asia	Bangladesh	LMI	Both	2007–2010	ELGAN-14, ELBW-15
Pinheiro 2010	South America	Brazil	UMI	ELBW	1999–2006	445
Piriyapokin 2020	Asia	Thailand	UMI	ELGAN	2005–2015	67
Poudel 2010	Asia	Nepal	LMI	Both	2005–2008	ELGAN-9, ELBW-23
Pourarian 2016	Middle East	Iran	UMI	ELBW	2014	28
Prabha 2014	Asia	India	LMI	Both	2008–2013	ELGAN-7, ELBW-6
Pradhan 2019	Asia	Bhutan	LMI	ELGAN	2017	18
Qazi 2011	Asia	Pakistan	LMI	ELGAN	2009	3
Qian 2008	Asia	China	UMI	ELGAN	2004–2005	45
Rezaeizadeh 2018	Middle East	Iran	UMI	Both	2013–2016	ELGAN-85, ELBW-127
Roy 2006	Asia	India	LMI	ELBW	2001–2005	36
Ruiz-Pelaez 2014	South America	Colombia	UMI	Both	2004	ELGAN-81, ELBW-86
Rylance 2013	Africa	Malawi	LI	ELBW	2010	71
Sivanandan 2016	Asia	India	LMI	Both	1999–2014	ELGAN-39, ELBW-125
Sabzehei 2013	Middle East	Iran	UMI	Both	2007–2010	ELGAN-84, ELBW-53
Saucedo 2008	North America	Mexico	UMI	ELBW	2002–2006	727
Sackey 2019	Africa	Ghana	LMI	Both	2011–2015	ELGAN-539, ELBW-560
Sahin 2014	Middle East	Turkey	UMI	ELGAN	2010–2012	109
Sahoo 2020	Asia	India	LMI	Both	2013–2018	ELGAN-95, ELBW-231
Saeidi 2009	Middle East	Iran	UMI	ELBW	2005–2006	52
Saeidi 2017	Middle East	Iran	UMI	ELBW	2013–2015	14
Saini 2016	Asia	India	LMI	Both	NA	ELGAN-7, ELBW-17
Salahuddin 2018	Asia	Pakistan	LMI	ELGAN	2015–2016	11
Saygili 2016	Middle East	Turkey	UMI	Both	2010–2013	ELGAN-203, ELBW-110
Sehgal 2004	Asia	India	LMI	Both	2000–2001	ELGAN-9, ELBW-52
Seid 2019	Africa	Ethiopia	LI	Both	2014–2017	ELGAN-25, ELBW-29
Serce 2014	Middle East	Turkey	UMI	Both	2010–2011	ELGAN-156, ELBW-179
Shrestha 2010	Asia	Nepal	LMI	ELGAN	2005	12
Singh2020	Asia	India	LMI	ELBW	2016	9
Siswanto 2018	Asia	Indonesia	UMI	Both	2005–2015	ELGAN-185, ELBW-182
Sousa 2017	South America	Brazil	UMI	ELBW	2014–2015	158
Sritipsukho 2017	Asia	Thailand	UMI	ELBW	2003–2006	21
Sun 2013	Asia	China	UMI	Both	2010	ELGAN-32, ELBW-28
Tamene 2020	Africa	Ethiopia	LI	Both	2017–2018	ELGAN-15, ELBW-23
Taqui 2008	Asia	Pakistan	LMI	Both	2003–2006	ELGAN-15, ELBW-14
Thakre 2017	Asia	India	LMI	ELBW	2011–2013	7
Thakur2013	Asia	India	LMI	Both	2010–2012	ELGAN-81, ELBW-283
Tosif 2019	Asia	Solomon Island	LMI	ELBW	2014–2016	45
Tran 2015	Asia	Vietnam	LMI	Both	2010–2011	ELGAN-29, ELBW-26
Trotman 2012	Caribbean	Jamaica	UMI	ELBW	1999–2010	286
Trotman 2006	Caribbean	Jamaica	UMI	Both	1995–2000	ELGAN-4, ELBW-7
Trotman 2007	Caribbean	Jamaica	UMI	ELBW	1987–2001	40
Trotman 2007a	Caribbean	Jamaica	UMI	Both	2002–2003	ELGAN-14, ELBW-47
Tshehla 2019	Africa	South Africa	UMI	ELBW	2016–2017	71
Ugwu 2010	Africa	Nigeria	LMI	ELBW	2002–2009	149
Undela 2019	Asia	India	LMI	Both	2017	ELGAN-6, ELBW-10
Velaphi 2005	Africa	South Africa	UMI	ELBW	2000–2002	453
Viau 2015	South America	Brazil	UMI	ELGAN	2006–2007	671
Vilanova 2019	South America	Brazil	UMI	ELBW	2000–2015	1325
Vural 2007	Middle East	Turkey	UMI	ELBW	2003–2005	19
Wang 2012	Asia	China	UMI	ELBW	2009–2010	160
Welbeck 2003	Africa	Ghana	LMI	Both	1995	ELGAN-382, ELBW-128
Winkler 2020	Africa	Tanzania	LMI	ELBW	2014–2018	8
Wu 2018	Asia	China	UMI	ELBW	2012–2015	60
Wu 2019	Asia	China	UMI	Both	2008–2017	ELGAN-2051, ELBW-1303
Xu 2013	Asia	China	UMI	Both	2010–2012	ELGAN-285, ELBW-99
Xu 2019	Asia	China	UMI	ELGAN	2013–2014	148
Yadav 2019	Asia	Nepal	LMI	Both	2019	ELGAN-6, ELBW-5
Yakoob 2014	Asia	India	LMI	ELGAN	1998–2003	38
Yau 2014	Asia	China	UMI	ELBW	2007–2012	131
Zea-Vera 2019	South America	Peru	UMI	ELBW	2012–2014	69
Zepeda Romero 2016	North America	Mexico	UMI	ELGAN	2012–2014	9
Zhang 2011	Asia	China	UMI	Both	1999–2009	ELGAN-29, ELBW-39
Zhang 2016	Asia	China	UMI	Both	2011–2012	ELGAN-13, ELBW-10
Zhang 2019	Asia	China	UMI	Both	2016–2017	ELGAN-89, ELBW-55
Zhang 2020	Asia	China	UMI	Both	2007–2016	ELGAN-32, ELBW-100
Zhou 2014	Asia	China	UMI	ELGAN	2010–2011	72
Zhu 2020	Asia	China	UMI	ELGAN	2010–2019	441
Ziadeh 2000	Middle East	Jordan	UMI	ELBW	1996–1999	12
Ziylan 2006	Middle East	Turkey	UMI	ELBW	1998–2003	114
Zuniga 2013	Africa	Burundi	LI	ELBW	2011	11

### Risk of bias

52 studies had a high risk of bias, 131 studies had an intermediate risk of bias and only 7 studies had a low risk of bias. Risk of bias was unclear in two studies. Only 31 studies had described the baseline characteristics of the neonates known to affect the survival and other morbidities such as receipt of antenatal corticosteroids, gender, small for gestational age (SGA) Status, Apgar scores and Level of NICU care. 83 studies had a prospective study design. The risk of bias of included studies is given in S2 Table in S1 File.

### Primary outcome

#### 1a. Survival until discharge for ELBW neonates

The overall survival was 34% (95% CI: 31% - 37%). There was significant heterogeneity in the survival rates between studies. Sub-group analysis to address the heterogeneity was done by analyzing studies based on the income status, region and country of origin. While there were widely varying survival rates between countries of similar economic status as well as region of origin, it was much lesser when studies were analyzed according to the country of origin.

**Fig 2:**
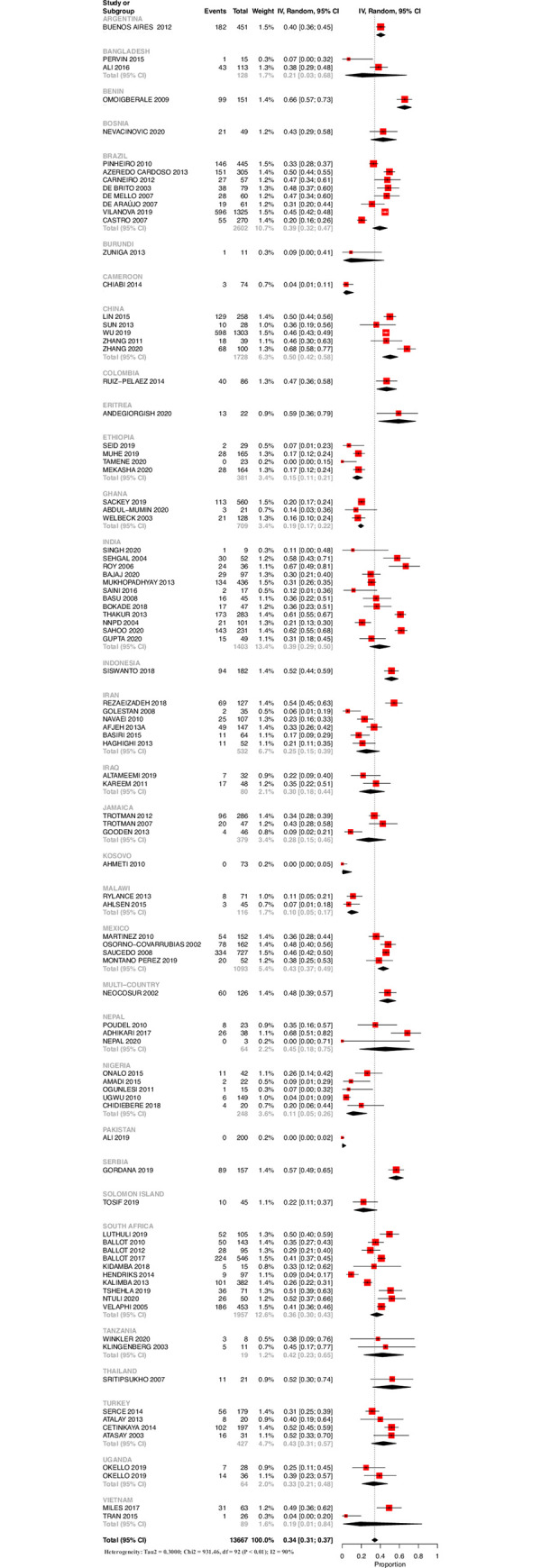
Survival until discharge for ELBW neonates based on country of origin.

For ELBW neonates, the survival for LI, LMI and UMI countries was 18% (11% - 28%), 28% (21% - 35%) and 39% (36% - 42%), respectively (S1 and S2 Figs in S1 File). While most of the countries from the African subcontinent had a survival rate of less than 20%, single center studies from Benin, Eritrea, South Africa and Tanzania reported better survival rates of more than 40% for ELBW neonates. While single center studies from China and India had reported survival rates of more than 60%, it was much lesser from the other Asian countries. While the countries from South America had a reported survival of 39% - 48%, the only middle-income country from North America, Mexico had a survival rate of 43% (37% - 49%). Amongst the countries from the Middle East, Turkey had the highest survival rate of 43% (31% - 57%). Country-wise survival outcomes for ELBW neonates is given in Fig 2. Publication bias was detected for this outcome (S3 Fig in S1 File)

#### 1b. Survival until discharge for ELGANs

**Fig 3:**
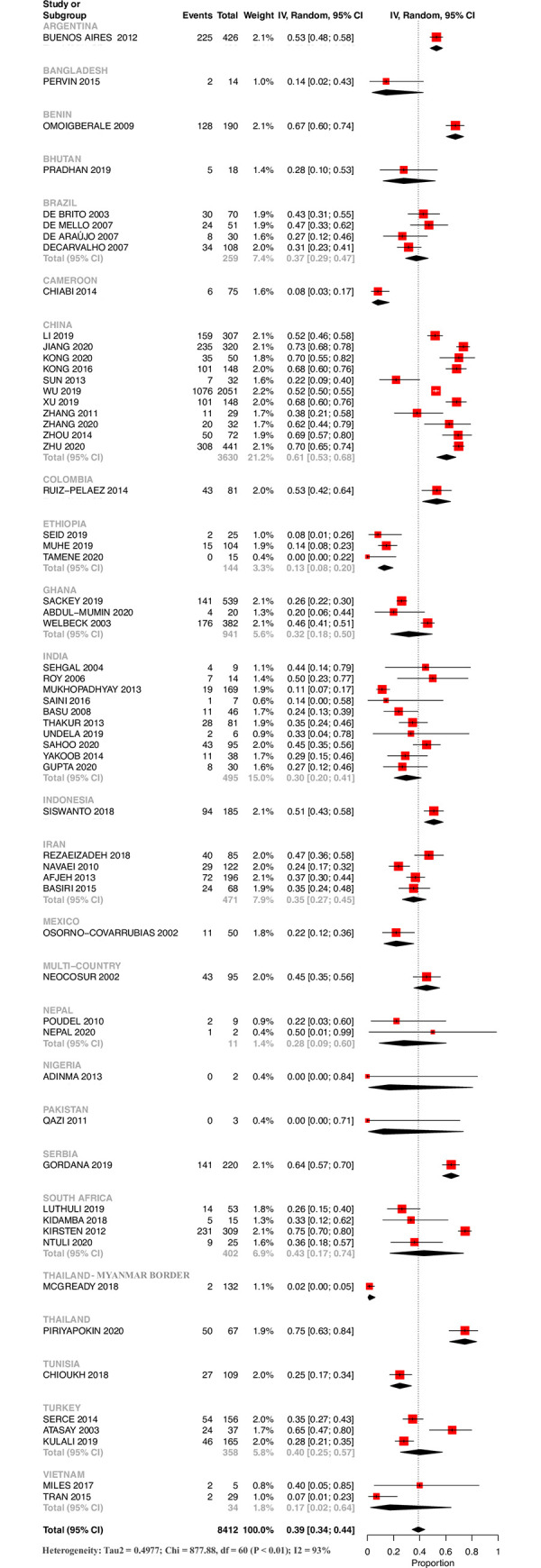
Survival until discharge for ELGANs based on country of origin.

Survival until discharge was 39% (34% - 44%). It was 13% (8% - 20%) for LI, 28% (21% - 36%) for LMI and 48% (42% - 53%) for UMI countries. Similar to ELBW survival, there was considerable variation between countries of similar economic classification and geographic region (S4-S6 Figs in S1 File) Single-center outlier studies from Benin, China, South Africa, Thailand and Turkey had reported relatively higher survival of more than 60% when compared to other countries (Fig 3). The survival as assessed by synthesizing data from multiple studies was highest in China [61% (53% - 68%)].

### Secondary outcomes

#### Short-term and long-term neurological outcomes

*2a*. *Severe IVH and PVL*. The rate of severe IVH and PVL in ELBW neonates was 14% (9% - 20%) and 7% (4% - 11%), respectively. For ELGANs, while the overall rate of severe IVH was 14% (11% - 19%). it was 6% (5% - 7%) for PVL (S7-S11 Figs in S1 File).

*2b*. *CP and NDI assessed at 24 months’ corrected age*. Only one study had reported NDI outcome for ELBW neonates which was 17% (7% - 34%). Meta-analysis of five studies revealed a NDI prevalence of 29% (23% - 37%) for ELGANs. The prevalence of CP in ELGAN population was 3% (1% - 9%) (S12-S14 Figs in S1 File)

#### Cardio-respiratory outcomes

*3a*. *PDA*. There was considerable variation in the reported rates of PDA requiring intervention between countries which was 15% (7% - 30%) for ELBW neonates and 50% (35% - 65%) for ELGANs (S15-S19 Figs in S1 File).

*3b*. *Requirement of invasive mechanical ventilation*. Higher rates of requirement of mechanical ventilation was reported from China which was 78% (65% - 92%) for ELBW neonates and 75% (65% - 84%) for ELGANs (S20 and S21 Figs in S1 File)

*3c*. *BPD assessed as oxygen requirement at 36 weeks’ PMA*. For ELBW neonates, BPD prevalence was 39% (30% - 48%). One study from South Africa had reported a relatively lower rate of 17% (6% - 33%). The overall prevalence of BPD was 37% (29% - 47%) in ELGANs. Three centers from China, India and Turkey had reported a higher rate of more than 60% (S22-S25 Figs in S1 File).

#### Sepsis

The rates of any sepsis and culture proven sepsis in ELBW neonates was 37% (28% - 48%) and 28% (21% - 35%), respectively. Rates of any and culture proven sepsis in ELGANs was 40% (25% - 57%) and 21% (12% - 32%), respectively (S26-S31 Figs in S1 File).

#### NEC stage II or more

The rates of NEC were uniform across countries with its prevalence being 8% (7% - 10%) for ELBW neonates as well as ELGANs. Publication bias was detected for studies reporting on NEC in ELBW neonates (S32-S35 Figs in S1 File).

#### EUGR

Two studies from the African sub-continent had reported on EUGR rates in ELBW neonates which was 88% (80% - 93%) (S36 Fig in S1 File).

#### ROP

Amongst ELBW neonates, any stage ROP, severe ROP and ROP requiring intervention rates was 49% (42% - 55%), 24% (19% - 30%) and 18% (12% - 27%), respectively. One study each from India, Iran and Pakistan had reported severe ROP rate of more than 50%. Also, some reports from China, Turkey and Jordan had ROP requiring intervention rates of more than 30%.

In ELGANs, any stage ROP, severe ROP and ROP requiring intervention was reported in 53% (46% - 59%), 22% (16% - 30%) and 20% (13% - 29%) of neonates, respectively. Single center reports from Thailand and Brazil had rates exceeding 40%.

Data related to ROP is given in S37-S48 Figs in S1 File.

#### Sensitivity analyses

*4a*. *Excluding studies with low sample size*. Analysis of survival outcome after excluding studies with low sample size did not result in any significant changes in the effect estimate when compared to the primary analysis for both ELBW neonates and ELGANs (S49 and S50 Figs in S1 File).

*4b*. *Comparison of survival between epoch 1*: *2000–2009 and epoch 2*: *2010–2019*. There was no significant difference in the survival rates between the two epochs for ELBW neonates (Test of moderators p value– 0.83) and ELGANs. (Test of moderators p value– 0.78) (S51 and S52 Figs in S1 File).

*4c*. *Analysing studies which had evaluated neonates with varying baseline sickness*. While of most of the studies were single centre studies, survival rates were reported by five studies for ELBW neonates and ELGANs with RDS. ELBW survival was 33% (20% - 50%) and ELGANs survival was 37% (28% - 47%) for RDS. While 41% (28% - 56%) of ELBW neonates who underwent invasive ventilation survived, the survival rate was 23% (9% - 48%) for ELGANs.

### Certainty of evidence

CoE for the primary outcome measure of survival until discharge for ELGANs was moderate and for ELBW neonates was low. While most of the included studies reporting on the primary outcome measure were prospective in design and started with high CoE, they were downgraded for serious inconsistency by one level. The outcome of survival until discharge for ELBW neonates was further downgraded by one level for publication bias. The CoE for other secondary outcomes were very low to low, with retrospective study design and heterogeneity being reasons for downgrading the CoE. The CoE is given in Table 2.

**Table 2:** Certainty of evidence for the primary outcome and all secondary outcomes for ELBW neonates and ELGANs.

**Outcomes**	**Number of studies**	**Number of neonates evaluated**	**Rate (95% CI)**	**Predominant type of studies**	**Risk of bias**	**Inconsistency**	**Indirectness**	**Imprecision**	**Publication bias**	**CoE**
**ELGANs**										
**Survival**	66	8,412	39% [34%- 44%]	Prospective	Not serious	Serious	Not serious	Not serious	None	Moderate
**Severe IVH**	8	2,001	14% [11%- 19%]	Retrospective	Serious	Not serious	Not serious	Serious	-	Very low
**PVL**	7	1,905	6% [5%-7%]	Retrospective	Not serious	Not serious	Not serious	Serious	-	Very low
**NDI**	4	243	29% [23%-37%]	Retrospective	Not serious	Not serious	Not serious	Serious	-	Very low
**CP**	2	105	3% [1%-9%]	Prospective	Not serious	Not serious	Not serious	Very serious	-	Low
**PDA requiring intervention**	5	511	50% [35%-65%]	Prospective	Not serious	Very serious	Not serious	Serious	-	Very low
**BPD**	7	2,809	37% [29%-47%]	Retrospective	Not serious	Serious	Not serious	Serious	None	Very low
**Any sepsis**	7	855	40% [25%-57%]	Retrospective	Not serious	Serious	Not serious	Serious	-	Very low
**Culture positive sepsis**	7	2,301	21% [12%- 32%]	Retrospective	Not serious	Not serious	Not serious	Serious	-	Very low
**NEC stage ≥II**	14	4,094	8% [7%- 10%]	Retrospective	Not serious	Not serious	Not serious	Serious	-	Very low
**Severe ROP**	14	6,003	22% [16%-30%]	Retrospective	Not serious	Serious	Not serious	Not serious	None	Very low
**ROP requiring intervention**	14	1,796	20% [13%- 29%]	Retrospective	Not serious	Not serious	Not serious	Not serious	None	Low
**Outcomes**			**Rate (95% CI)**	**Predominat type of studies**	**Risk of bias**	**Inconsistency**	**Indirectness**	**Imprecision**	**Publication bias**	**Overall Certainty of Evidence**
**ELBW neonates**										
**Survival**	92	13,667	34% [31%-37%]	Prospective	Not serious	Serious	Not serious	Not serious	Serious	Low
**Severe IVH**	11	1.098	14% [9%- 20%]	Retrospective	Not serious	Not serious	Not serious	Not serious	None	Low
**PVL**	8	616	7% [4%-11%]	Retrospective	Not serious	Not serious	Not serious	Serious	-	Very low
**NDI**	1	30	17% [6%-35%]	Prospective	Not serious	-	Not serious	Very serious	-	Low
**PDA requiring intervention**	4	486	15% [7%-30%]	Retrospective	Not serious	Serious	Not serious	Serious	-	Very low
**BPD**	10	594	39% [30%-48%]	Retrospective	Not serious	Serious	Not serious	Serious	None	Very low
**Any sepsis**	12	1,284	37% [28%-48%]	Prospective	Not serious	Serious	Not serious	Serious	None	Low
**Culture positive sepsis**	11	1,465	28% [21%-35%]	Retrospective	Not serious	Serious	Not serious	Serious	None	Very low
**NEC stage ≥II**	14	2,914	8% [7%-10%]	Retrospective	Not serious	Not serious	Not serious	Serious	Serious	Very low
**EUGR**	2	107	88% [80%-93%]	Retrospective	Serious	Not serious	Not serious	Serious	-	Very low
**Severe ROP**	17	5,413	24% [19%-30%]	Retrospective	Not serious	Not serious	Not serious	Not serious	None	Low
**ROP requiring intervention**	11	1,293	18% [12%-27%]	Retrospective	Not serious	Not serious	Not serious	Not serious	None	Low

## Discussion

This systematic review and meta-analysis included 192 studies (ELBW neonates– 22,278; ELGANs– 18,338) from LMICs situated in different geographical regions across the globe. To the best of our knowledge, this is the only systematic review evaluating survival and morbidities in ELGANs or ELBW neonates from LMICs.

The primary outcome of the review was the proportion of neonates who had survived until discharge. The results of this study indicate that the overall survival until discharge of ELBW neonates was 34% and ELGANs was 39%, with significant heterogeneity between studies based on the income status, region as well as country of origin. These survival rates are much lower than that reported from HICs [22]. Myrhaug et al. in their meta-analysis (2019) had reported a survival rate of 74% at 25 weeks’ and 90% at 27 weeks’ amongst neonates born alive in HICs [22]. Such stark differences in survival between LMICs and HICs can be explained by many reasons. Attitudes towards providing life-saving intervention to these immature infants are quite different between LMICs and HICs. In LMICs, the focus of healthcare programmes to reduce NMR comprise predominantly of cost-effective high impact interventions comprising of early essential newborn care services which are predominantly targeted to address mortality in relatively bigger neonates [6, 26]. Decreasing mortality rates in ELGANs in LMICs through establishment of higher level NICUs might impose further financial burden as well as risk of inequity, diverting resources from the relatively more mature neonates. Further, absence of healthcare insurance schemes in LMICs result in financial constraints for parents who eventually have to bear most of the costs incurred [27]. Finally, ‘denominator bias’ due to lack of surveillance data and consequent inconsistent reporting from LMICs regarding live births versus NICU admissions might also result in significant variability in survival rates in LMICs when compared to HICs [28, 29].

There was significant variation in survival between LMICs as well. Similar findings are also seen in the studies published from HICs [30]. While some studies from HICs have pointed out factors such as differing gestational age cut-offs for instituting active care, selective versus comprehensive care and variations in clinical care practices for babies born at less than 25 weeks’ gestation being some of the reasons for varying survival rates between different countries, such comparative studies are lacking for LMICs [31–33]. Differing survival rates between studies from the same LMIC might be explained by the survival gap between rural and urban areas [34]. Whether there are any differences in survival between private versus public sector systems in LMICs still remains a contested topic, it was beyond the scope of our review to look into the same [35]. Differences in health policy, financial resources, access to and use of health services, infrastructure, and economic development might also explain the significant variability in survival between countries of similar economic status [36–38].

Sensitivity analysis indicated that there was no significant differences in the survival rates between the two epochs (epoch 1: 2000–2009 and epoch 2: 2010–2019). Similar findings were noted in the meta-analysis by Myrhaug et al. for HICs [22]. However, some neonatal network studies from other HICs have shown both an improving as well as static trend for survival in the past few decades [39–43]. The rates of some secondary outcomes such as NEC and PVL were comparable with data from HICs [40–42]. This might be due to the fact that many of the HICs provide active care for neonates born from 22 weeks’ gestation who are at the highest risk of major brain injury, whilst most of the ELBW neonates and ELGANs enrolled in studies from LMICs were of higher gestational ages ranging from 25–27 weeks [31, 33, 40, 44]. However, rates of all other morbidities were higher in LMICs when compared to HICs. Information from limited studies in the meta-analysis have shown that the rates of NDI was 17% (7% - 34%) and 29% (23% - 37%) in ELBW neonates and ELGANs, respectively. Milner et al. in their systematic review and meta-analysis of long-term neurodevelopmental outcomes of preterm VLBW neonates in resource limited settings had reported a lower NDI rate of 21.4% (11.6% - 30.8%) [12]. This could be because of the fact that Milner et al.’s study consisted of neonates who were gestationally more mature. Also, many studies from HICs such as Croatia, Korea, Poland and Taiwan were included in their study.

This systematic review had some limitations. There was considerable heterogeneity in the various outcome measures despite performing multiple sensitivity and sub-group analyses to address the same. The analysis might be limited by denominator bias as survival rates could not be assessed based on different denominators such as live births versus NICU admissions, due to inconsistent reporting in studies. Accurate gestational age assessment is still a major issue in LMICs and might have influenced our final estimates. Further, analysis by stratification on the basis of gestational age or birth weight could not be performed for ELGANs and ELBW neonates. Important confounders determining survival such as antenatal corticosteroid use, multiple gestation, SGA status, gender, chorioamnionitis and level of NICU care were not reported by majority of the included studies, thus precluding any sensitivity analysis based on these parameters. The overall CoE was very low to low for all the secondary outcomes. Finally, though the literature search included the standard databases as recommended by the Cochrane group, studies from LMICs might be more likely to be published in alternative databases as well.

## Conclusion

Mortality and morbidity of ELBW neonates and ELGANs is still a huge burden in LMICs, with significant differences in their occurrence between countries of similar economic status and geographical region of location. For ELBW neonates, the survival for LI, LMI and UMI countries was 18% (11% - 28%), 28% (21% - 35%) and 39% (36% - 42%), respectively. For ELGANs, it was 13% (8% - 20%) for LI, 28% (21% - 36%) for LMI and 48% (42% - 53%) for UMI countries. These are significantly lower than the survival rates reported from HICs. The CoE for most of the outcomes was very low to low, emphasizing the need for surveillance and high quality prospective cohort studies from LMICs on this sub-group of vulnerable neonates. Such studies should provide data related to still births, live births, delivery room deaths, important baseline characteristics such as antenatal corticosteroid use, multiple gestation status, SGA, gender, chorioamnionitis and level of NICU care, and sub-group data on different gestational ages or birth weights for ELGANs or ELBW neonates. Such data would not only quantify the burden of mortality and morbidity in these preterm infants, but also enable evaluating the post-implementation impact of important public health interventions.

## Supporting information

S1 Checklist PRISMA checklist for reporting of the review. (DOC)

S1 File This file with tables and figures showing various outcomes assessed, literature search strategy, risk of bias of included studies and references of included studies. (PDF)

## Abbreviations

BPD: Bronchopulmonary dysplasia
CoE: Certainty of evidence
ELBW: Extremely low birth weight
ELGANs: Extremely low gestational age neonates
EUGR: Extrauterine growth retardation
HICs: High income countries
IVH: Intraventricular hemorrhage
LMICs: Low- and middle-income countries
MA: Meta-analysis
NEC: Necrotising enterocolitis
NDI: Neurodevelopmental impairment
NMR: Neonatal mortality rate
PDA: Patent ductus arteriosus
PMA: Post-menstrual age
PVL: Periventricular leukomalacia
ROB: Risk of bias
ROP: Retinopathy of prematurity
SR: Systematic review
SDG: Sustainable Development Goals
VLBW: Very low birth weight
