# Supplementary material for: ELBW and ELGAN outcomes in developing nations–Systematic review and meta-analysis
Source: PLoS One. 2021 Aug 5;16(8):e0255352. doi: 10.1371/journal.pone.0255352 (PMC8342042; doi:10.1371/journal.pone.0255352)
Supplement: S1 File — (PDF) [file pone.0255352.s002.pdf]

S1 File

ELBW and ELGAN outcomes in developing nations – Systematic Review and meta-analysis

Supplement Figure 1: Primary outcome - survival until discharge for ELBW neonates analyzed based on income classification.
Supplement Figure 2: Primary outcome - survival until discharge for ELBW neonates analyzed based on geographic region of origin.
Supplement Figure 3: Publication bias for the primary outcome - survival until discharge for ELBW neonates.
Supplement Figure 4: Primary outcome - survival until discharge for ELGANs analyzed based on income classification.
Supplement Figure 5: Primary outcome - survival until discharge for ELGANs analyzed based on geographic region of origin.
Supplement Figure 6: Publication bias for the primary outcome - survival until discharge for ELGANs.
Supplement Figure 7: Secondary outcome - Severe IVH in ELBW neonates.
Supplement Figure 8: Publication bias for the secondary outcome - severe IVH in ELBW neonates.
Supplement Figure 9: Secondary outcome - Severe IVH in ELGANs.
Supplement Figure 10: Secondary outcome- PVL in ELBW neonates.
Supplement Figure 11: Secondary outcome - PVL in ELGANs.
Supplement Figure 12: Secondary outcome - NDI in ELBW neonates.
Supplement Figure 13: Secondary outcome- NDI in ELGANs.
Supplement Figure 14: Secondary outcome - CP in ELGANs.
Supplement Figure 15: Secondary outcome- Any PDA in ELBW neonates.
Supplement Figure 16: Secondary outcome - Any PDA in ELGANs.
Supplement Figure 17: Publication bias for the secondary outcome - Any PDA in ELBW neonates.
Supplement Figure 18: Secondary outcome- PDA requiring intervention in ELBW neonates.
Supplement Figure 19: Secondary outcome - PDA requiring intervention in ELGANs.
Supplement Figure 20: Secondary outcome- Requirement of invasive mechanical ventilation in ELBW neonates.
Supplement Figure 21: Secondary outcome - Requirement of invasive mechanical ventilation in ELGANs.
Supplement Figure 22: Secondary outcome- BPD in ELBW neonates.
Supplement Figure 23: Publication bias for the secondary outcome - BPD in ELBW neonates.
Supplement Figure 24: Secondary outcome- BPD in ELGANs.
Supplement Figure 25: Publication bias for the secondary outcome - BPD in ELGANs.
Supplement Figure 26: Secondary outcome- Any sepsis in ELBW neonates.
Supplement Figure 27: Publication bias for the secondary outcome - Any sepsis in ELBW neonates.
Supplement Figure 28: Secondary outcome- Any sepsis in ELGANs
Supplement Figure 29: Secondary outcome- Culture proven sepsis in ELBW neonates
Supplement Figure 30: Publication bias for the secondary outcome - Culture proven sepsis in ELBW neonates.
Supplement Figure 31: Secondary outcome- Culture proven sepsis in ELGANs
Supplement Figure 32: Secondary outcome- NEC in ELBW neonates
Supplement Figure 33: Publication bias for the secondary outcome - NEC in ELBW neonates.
Supplement Figure 34: Secondary outcome- NEC in ELGANs
Supplement Figure 35: Publication bias for the secondary outcome - NEC in ELGANs.

Supplement Figure 36: Secondary outcome- EUGR in ELBWs
Supplement Figure 37: Secondary outcome- Any ROP in ELBW neonates
Supplement Figure 38: Publication bias for the secondary outcome - Any ROP in ELBW neonates.
Supplement Figure 39: Secondary outcome- Any ROP in ELGANs
Supplement Figure 40: Publication bias for the secondary outcome - Any ROP in ELGANs.
Supplement Figure 41: Secondary outcome- Severe ROP in ELBW neonates
Supplement Figure 42: Publication bias for the secondary outcome - Severe ROP in ELBW neonates.
Supplement Figure 43: Secondary outcome- Severe ROP in ELGANs
Supplement Figure 44: Publication bias for the secondary outcome - Severe ROP in ELGANs.
Supplement Figure 45: Secondary outcome- ROP requiring intervention in ELBW neonates
Supplement Figure 46: Publication bias for the secondary outcome - ROP requiring intervention in ELBW neonates.
Supplement Figure 47: Secondary outcome- ROP requiring intervention in ELGANs
Supplement Figure 48: Publication bias for the secondary outcome - ROP requiring intervention in ELGANs.
Supplement Figure 49: Sensitivity analysis - primary outcome - survival until discharge for ELBW neonates analyzed based on country of origin after excluding small sample size studies.
Supplement Figure 50: Sensitivity analysis - primary outcome - survival until discharge for ELGANs analyzed based on country of origin after excluding small sample size studies.
Supplement Figure 51: Sensitivity analysis - primary outcome - Comparison of two epochs (2000-2009 & 2010-2020) for survival until discharge for ELBW neonates analyzed based on country of origin.
Supplement Figure 52: Sensitivity analysis - primary outcome - Comparison of two epochs (2000-2009 & 2010-2020) for survival until discharge for ELGANs analyzed based on country of origin.
Supplement Table 1: Literature search strategy.
Supplement Table 2: Risk of bias of included studies.
Supplement References of included studies

Supplement Figure 1: Primary outcome - survival until discharge for ELBW neonates analyzed based on income classification.

Study or Subgroup	Events	Total	Weight	IV, Random, 95% CI
LI				
OKELLO 2019	7	28	0.9%	0.25 [0.11; 0.45]
OKELLO 2019	14	36	1.1%	0.39 [0.23; 0.57]
RYLANCE 2013	8	71	1.0%	0.11 [0.05; 0.21]
SEID 2019	2	29	0.5%	0.07 [0.01; 0.23]
MUHE 2019	28	165	1.3%	0.17 [0.12; 0.24]
AHLSEN 2015	3	45	0.7%	0.07 [0.01; 0.18]
ANDEGIORGISH 2020	13	22	0.9%	0.59 [0.36; 0.79]
TAMENE 2020	0	23	0.2%	0.00 [0.00; 0.15]
ZUNIGA 2013	1	11	0.3%	0.09 [0.00; 0.41]
MEKASHA 2020	28	164	1.3%	0.17 [0.12; 0.24]
Total (95% CI)		594	8.3%	0.18 [0.11; 0.28]
LMI				
SINGH 2020	1	9	0.3%	0.11 [0.00; 0.48]
SEHGAL 2004	30	52	1.2%	0.58 [0.43; 0.71]
PERVIN 2015	1	15	0.3%	0.07 [0.00; 0.32]
ROY 2006	24	36	1.1%	0.67 [0.49; 0.81]
POUDEL 2010	8	23	0.9%	0.35 [0.16; 0.57]
SACKEY 2019	113	560	1.5%	0.20 [0.17; 0.24]
OMOIGBERALE 2009	99	151	1.4%	0.66 [0.57; 0.73]
ONALO 2015	11	42	1.1%	0.26 [0.14; 0.42]
BAJAJ 2020	29	97	1.3%	0.30 [0.21; 0.40]
MILES 2017	31	63	1.2%	0.49 [0.36; 0.62]
MUKHOPADHYAY 2013	134	436	1.5%	0.31 [0.26; 0.35]
SAINI 2016	2	17	0.5%	0.12 [0.01; 0.36]
ALI 2019	0	200	0.2%	0.00 [0.00; 0.02]
ABDUL–MUMIN 2020	3	21	0.7%	0.14 [0.03; 0.36]
ADHIKARI 2017	26	38	1.1%	0.68 [0.51; 0.82]
ALI 2016	43	113	1.3%	0.38 [0.29; 0.48]
AMADI 2015	2	22	0.5%	0.09 [0.01; 0.29]
BASU 2008	16	45	1.1%	0.36 [0.22; 0.51]
BOKADE 2018	17	47	1.2%	0.36 [0.23; 0.51]
OGUNLESI 2011	1	15	0.3%	0.07 [0.00; 0.32]
THAKUR 2013	173	283	1.4%	0.61 [0.55; 0.67]
TOSIF 2019	10	45	1.1%	0.22 [0.11; 0.37]
TRAN 2015	1	26	0.3%	0.04 [0.00; 0.20]
UGWU 2010	6	149	1.0%	0.04 [0.01; 0.09]
NNPD 2004	21	101	1.3%	0.21 [0.13; 0.30]
WELBECK 2003	21	128	1.3%	0.16 [0.10; 0.24]
WINKLER 2020	3	8	0.5%	0.38 [0.09; 0.76]
SAHOO 2020	143	231	1.4%	0.62 [0.55; 0.68]
GUPTA 2020	15	49	1.1%	0.31 [0.18; 0.45]
CHIABI 2014	3	74	0.7%	0.04 [0.01; 0.11]
CHIDIEBERE 2018	4	20	0.7%	0.20 [0.06; 0.44]
KLINGENBERG 2003	5	11	0.7%	0.45 [0.17; 0.77]
NEPAL 2020	0	3	0.2%	0.00 [0.00; 0.71]
Total (95% CI)		3130	30.3%	0.28 [0.21; 0.35]
UMI				
PINHEIRO 2010	146	445	1.5%	0.33 [0.28; 0.37]
RUIZ–PELAEZ 2014	40	86	1.3%	0.47 [0.36; 0.58]
REZAEIZADEH 2018	69	127	1.4%	0.54 [0.45; 0.63]
ALTAMEEMI 2019	7	32	0.9%	0.22 [0.09; 0.40]
SISWANTO 2018	94	182	1.4%	0.52 [0.44; 0.59]
MARTINEZ 2010	54	152	1.4%	0.36 [0.28; 0.44]
OSORNO–COVARRUBIAS 2002	78	162	1.4%	0.48 [0.40; 0.56]
SERCE 2014	56	179	1.4%	0.31 [0.25; 0.39]
GOLESTAN 2008	2	35	0.5%	0.06 [0.01; 0.19]
LUTHULI 2019	52	105	1.3%	0.50 [0.40; 0.59]
NAVAEI 2010	25	107	1.3%	0.23 [0.16; 0.33]
SAUCEDO 2008	334	727	1.5%	0.46 [0.42; 0.50]
AFJEH 2013A	49	147	1.4%	0.33 [0.26; 0.42]
AHMETI 2010	0	73	0.2%	0.00 [0.00; 0.05]
ATALAY 2013	8	20	0.9%	0.40 [0.19; 0.64]
AZEREDO CARDOSO 2013	151	305	1.4%	0.50 [0.44; 0.55]
BALLOT 2010	50	143	1.4%	0.35 [0.27; 0.43]
BALLOT 2012	28	95	1.3%	0.29 [0.21; 0.40]
BALLOT 2017	224	546	1.5%	0.41 [0.37; 0.45]
BASIRI 2015	11	64	1.1%	0.17 [0.09; 0.29]
CARNEIRO 2012	27	57	1.2%	0.47 [0.34; 0.61]
CETINKAYA 2014	102	197	1.4%	0.52 [0.45; 0.59]
DE BRITO 2003	38	79	1.3%	0.48 [0.37; 0.60]
DE MELLO 2007	28	60	1.2%	0.47 [0.34; 0.60]
ATASAY 2003	16	31	1.1%	0.52 [0.33; 0.70]
DE ARAÚJO 2007	19	61	1.2%	0.31 [0.20; 0.44]
KAREEM 2011	17	48	1.2%	0.35 [0.22; 0.51]
KIDAMBA 2018	5	15	0.8%	0.33 [0.12; 0.62]
LIN 2015	129	258	1.4%	0.50 [0.44; 0.56]
HENDRIKS 2014	9	97	1.1%	0.09 [0.04; 0.17]
KALIMBA 2013	101	382	1.4%	0.26 [0.22; 0.31]
SRITIPSUKHO 2007	11	21	0.9%	0.52 [0.30; 0.74]
SUN 2013	10	28	1.0%	0.36 [0.19; 0.56]
TSHEHLA 2019	36	71	1.3%	0.51 [0.39; 0.63]
NEOCOSUR 2002	60	126	1.4%	0.48 [0.39; 0.57]
BUENOS AIRES 2012	182	451	1.5%	0.40 [0.36; 0.45]
TROTMAN 2012	96	286	1.4%	0.34 [0.28; 0.39]
TROTMAN 2007	20	47	1.2%	0.43 [0.28; 0.58]
NTULI 2020	26	50	1.2%	0.52 [0.37; 0.66]
VELAPHI 2005	186	453	1.5%	0.41 [0.36; 0.46]
VILANOVA 2019	596	1325	1.5%	0.45 [0.42; 0.48]
GORDANA 2019	89	157	1.4%	0.57 [0.49; 0.65]
WU 2019	598	1303	1.5%	0.46 [0.43; 0.49]
ZHANG 2011	18	39	1.1%	0.46 [0.30; 0.63]
ZHANG 2020	68	100	1.3%	0.68 [0.58; 0.77]
GOODEN 2013	4	46	0.8%	0.09 [0.02; 0.21]
HAGHIGHI 2013	11	52	1.1%	0.21 [0.11; 0.35]
CASTRO 2007	55	270	1.4%	0.20 [0.16; 0.26]
NEVACINOVIC 2020	21	49	1.2%	0.43 [0.29; 0.58]
MONTANO PEREZ 2019	20	52	1.2%	0.38 [0.25; 0.53]
Total (95% CI)		9943	61.3%	0.39 [0.36; 0.42]
**Total (95% CI)**		**13667**	**100.0%**	**0.34 [0.31; 0.37]**

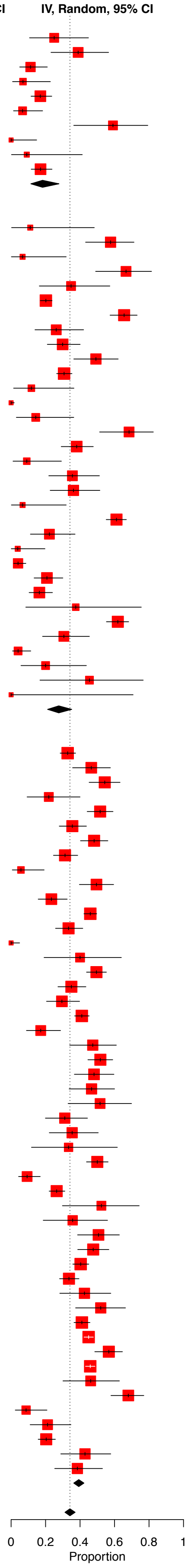

Heterogeneity: Tau2 = 0.3000; Chi2 = 931.46, df = 92 (P < 0.01); I2 = 90%

Supplement Figure 2: Primary outcome - survival until discharge for ELBW neonates analyzed based on geographic region of origin.

Study or Subgroup	Events	Total	Weight	IV, Random, 95% CI
**AFRICA**				
OKELLO 2019	7	28	0.9%	0.25 [0.11; 0.45]
OKELLO 2019	14	36	1.1%	0.39 [0.23; 0.57]
RYLANCE 2013	8	71	1.0%	0.11 [0.05; 0.21]
SEID 2019	2	29	0.5%	0.07 [0.01; 0.23]
SACKEY 2019	113	560	1.5%	0.20 [0.17; 0.24]
OMOIGBERALE 2009	99	151	1.4%	0.66 [0.57; 0.73]
ONALO 2015	11	42	1.1%	0.26 [0.14; 0.42]
LUTHULI 2019	52	105	1.3%	0.50 [0.40; 0.59]
MUHE 2019	28	165	1.3%	0.17 [0.12; 0.24]
ABDUL–MUMIN 2020	3	21	0.7%	0.14 [0.03; 0.36]
AHLSEN 2015	3	45	0.7%	0.07 [0.01; 0.18]
AMADI 2015	2	22	0.5%	0.09 [0.01; 0.29]
ANDEGIORGISH 2020	13	22	0.9%	0.59 [0.36; 0.79]
BALLOT 2010	50	143	1.4%	0.35 [0.27; 0.43]
BALLOT 2012	28	95	1.3%	0.29 [0.21; 0.40]
BALLOT 2017	224	546	1.5%	0.41 [0.37; 0.45]
KIDAMBA 2018	5	15	0.8%	0.33 [0.12; 0.62]
HENDRIKS 2014	9	97	1.1%	0.09 [0.04; 0.17]
KALIMBA 2013	101	382	1.4%	0.26 [0.22; 0.31]
TSHEHLA 2019	36	71	1.3%	0.51 [0.39; 0.63]
TAMENE 2020	0	23	0.2%	0.00 [0.00; 0.15]
OGUNLESI 2011	1	15	0.3%	0.07 [0.00; 0.32]
NTULI 2020	26	50	1.2%	0.52 [0.37; 0.66]
UGWU 2010	6	149	1.0%	0.04 [0.01; 0.09]
VELAPHI 2005	186	453	1.5%	0.41 [0.36; 0.46]
WELBECK 2003	21	128	1.3%	0.16 [0.10; 0.24]
WINKLER 2020	3	8	0.5%	0.38 [0.09; 0.76]
ZUNIGA 2013	1	11	0.3%	0.09 [0.00; 0.41]
MEKASHA 2020	28	164	1.3%	0.17 [0.12; 0.24]
CHIABI 2014	3	74	0.7%	0.04 [0.01; 0.11]
CHIDIEBERE 2018	4	20	0.7%	0.20 [0.06; 0.44]
KLINGENBERG 2003	5	11	0.7%	0.45 [0.17; 0.77]
Total (95% CI)		3752	31.3%	0.24 [0.19; 0.30]
**ASIA**				
SINGH 2020	1	9	0.3%	0.11 [0.00; 0.48]
SEHGAL 2004	30	52	1.2%	0.58 [0.43; 0.71]
PERVIN 2015	1	15	0.3%	0.07 [0.00; 0.32]
ROY 2006	24	36	1.1%	0.67 [0.49; 0.81]
POUDEL 2010	8	23	0.9%	0.35 [0.16; 0.57]
SISWANTO 2018	94	182	1.4%	0.52 [0.44; 0.59]
BAJAJ 2020	29	97	1.3%	0.30 [0.21; 0.40]
MILES 2017	31	63	1.2%	0.49 [0.36; 0.62]
MUKHOPADHYAY 2013	134	436	1.5%	0.31 [0.26; 0.35]
SAINI 2016	2	17	0.5%	0.12 [0.01; 0.36]
ALI 2019	0	200	0.2%	0.00 [0.00; 0.02]
ADHIKARI 2017	26	38	1.1%	0.68 [0.51; 0.82]
ALI 2016	43	113	1.3%	0.38 [0.29; 0.48]
BASU 2008	16	45	1.1%	0.36 [0.22; 0.51]
BOKADE 2018	17	47	1.2%	0.36 [0.23; 0.51]
LIN 2015	129	258	1.4%	0.50 [0.44; 0.56]
SRITIPSUKHO 2007	11	21	0.9%	0.52 [0.30; 0.74]
SUN 2013	10	28	1.0%	0.36 [0.19; 0.56]
THAKUR 2013	173	283	1.4%	0.61 [0.55; 0.67]
TOSIF 2019	10	45	1.1%	0.22 [0.11; 0.37]
TRAN 2015	1	26	0.3%	0.04 [0.00; 0.20]
NNPD 2004	21	101	1.3%	0.21 [0.13; 0.30]
WU 2019	598	1303	1.5%	0.46 [0.43; 0.49]
ZHANG 2011	18	39	1.1%	0.46 [0.30; 0.63]
ZHANG 2020	68	100	1.3%	0.68 [0.58; 0.77]
SAHOO 2020	143	231	1.4%	0.62 [0.55; 0.68]
GUPTA 2020	15	49	1.1%	0.31 [0.18; 0.45]
NEPAL 2020	0	3	0.2%	0.00 [0.00; 0.71]
Total (95% CI)		3860	28.7%	0.41 [0.35; 0.47]
**CARIBBEAN**				
TROTMAN 2012	96	286	1.4%	0.34 [0.28; 0.39]
TROTMAN 2007	20	47	1.2%	0.43 [0.28; 0.58]
GOODEN 2013	4	46	0.8%	0.09 [0.02; 0.21]
Total (95% CI)		379	3.4%	0.28 [0.15; 0.46]
**EUROPE**				
AHMETI 2010	0	73	0.2%	0.00 [0.00; 0.05]
GORDANA 2019	89	157	1.4%	0.57 [0.49; 0.65]
NEVACINOVIC 2020	21	49	1.2%	0.43 [0.29; 0.58]
Total (95% CI)		279	2.8%	0.35 [0.15; 0.63]
**MIDEAST**				
REZAEIZADEH 2018	69	127	1.4%	0.54 [0.45; 0.63]
ALTAMEEMI 2019	7	32	0.9%	0.22 [0.09; 0.40]
SERCE 2014	56	179	1.4%	0.31 [0.25; 0.39]
GOLESTAN 2008	2	35	0.5%	0.06 [0.01; 0.19]
NAVAEI 2010	25	107	1.3%	0.23 [0.16; 0.33]
AFJEH 2013A	49	147	1.4%	0.33 [0.26; 0.42]
ATALAY 2013	8	20	0.9%	0.40 [0.19; 0.64]
BASIRI 2015	11	64	1.1%	0.17 [0.09; 0.29]
CETINKAYA 2014	102	197	1.4%	0.52 [0.45; 0.59]
ATASAY 2003	16	31	1.1%	0.52 [0.33; 0.70]
KAREEM 2011	17	48	1.2%	0.35 [0.22; 0.51]
HAGHIGHI 2013	11	52	1.1%	0.21 [0.11; 0.35]
Total (95% CI)		1039	13.6%	0.32 [0.24; 0.41]
**NAMERICA**				
MARTINEZ 2010	54	152	1.4%	0.36 [0.28; 0.44]
OSORNO–COVARRUBIAS 2002	78	162	1.4%	0.48 [0.40; 0.56]
SAUCEDO 2008	334	727	1.5%	0.46 [0.42; 0.50]
MONTANO PEREZ 2019	20	52	1.2%	0.38 [0.25; 0.53]
Total (95% CI)		1093	5.4%	0.43 [0.37; 0.49]
**SAMERICA**				
PINHEIRO 2010	146	445	1.5%	0.33 [0.28; 0.37]
RUIZ–PELAEZ 2014	40	86	1.3%	0.47 [0.36; 0.58]
AZEREDO CARDOSO 2013	151	305	1.4%	0.50 [0.44; 0.55]
CARNEIRO 2012	27	57	1.2%	0.47 [0.34; 0.61]
DE BRITO 2003	38	79	1.3%	0.48 [0.37; 0.60]
DE MELLO 2007	28	60	1.2%	0.47 [0.34; 0.60]
DE ARAÚJO 2007	19	61	1.2%	0.31 [0.20; 0.44]
NEOCOSUR 2002	60	126	1.4%	0.48 [0.39; 0.57]
BUENOS AIRES 2012	182	451	1.5%	0.40 [0.36; 0.45]
VILANOVA 2019	596	1325	1.5%	0.45 [0.42; 0.48]
CASTRO 2007	55	270	1.4%	0.20 [0.16; 0.26]
Total (95% CI)		3265	14.9%	0.41 [0.35; 0.46]
**Total (95% CI)**		**13667**	**100.0%**	**0.34 [0.31; 0.37]**

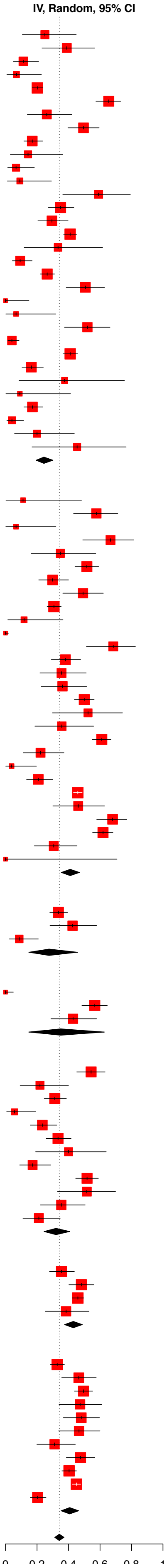

Heterogeneity: Tau2 = 0.3000; Chi2 = 931.46, df = 92 (P < 0.01); I2 = 90%

Supplement Figure 3: Publication bias for the primary outcome - survival until discharge for ELBW neonates.

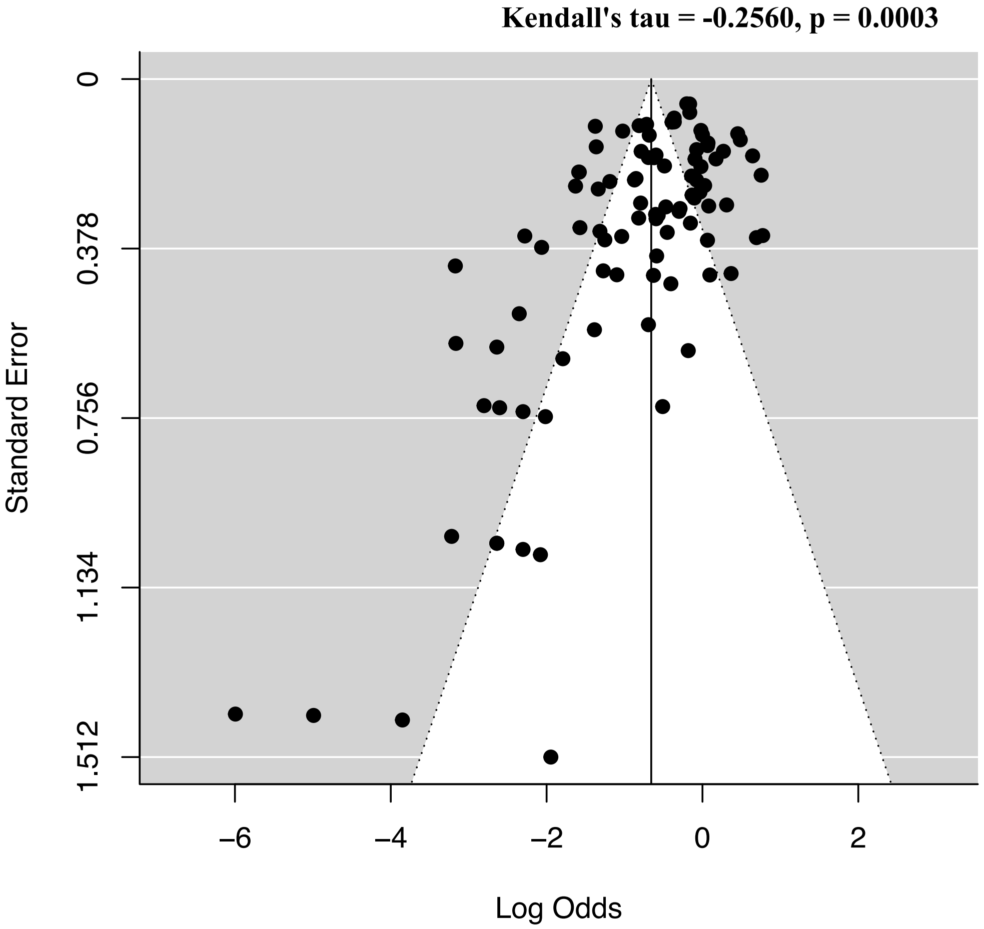

Supplement Figure 4: Primary outcome - survival until discharge for ELGANs analyzed based on income classification.

Study or Subgroup	Events	Total	Weight	IV, Random, 95% CI
**LI**				
SEID 2019	2	25	1.0%	0.08 [0.01; 0.26]
MUHE 2019	15	104	1.9%	0.14 [0.08; 0.23]
TAMENE 2020	0	15	0.4%	0.00 [0.00; 0.22]
Total (95% CI)		144	3.3%	0.13 [0.08; 0.20]
**LMI**				
SEHGAL 2004	4	9	1.1%	0.44 [0.14; 0.79]
PRADHAN 2019	5	18	1.4%	0.28 [0.10; 0.53]
PERVIN 2015	2	14	1.0%	0.14 [0.02; 0.43]
ROY 2006	7	14	1.4%	0.50 [0.23; 0.77]
POUDEL 2010	2	9	0.9%	0.22 [0.03; 0.60]
SACKEY 2019	141	539	2.1%	0.26 [0.22; 0.30]
OMOIGBERALE 2009	128	190	2.1%	0.67 [0.60; 0.74]
MILES 2017	2	5	0.8%	0.40 [0.05; 0.85]
MUKHOPADHYAY 2013	19	169	1.9%	0.11 [0.07; 0.17]
SAINI 2016	1	7	0.6%	0.14 [0.00; 0.58]
ABDUL–MUMIN 2020	4	20	1.3%	0.20 [0.06; 0.44]
ADINMA 2013	0	2	0.4%	0.00 [0.00; 0.84]
BASU 2008	11	46	1.7%	0.24 [0.13; 0.39]
THAKUR 2013	28	81	1.9%	0.35 [0.24; 0.46]
TRAN 2015	2	29	1.0%	0.07 [0.01; 0.23]
UNDELA 2019	2	6	0.9%	0.33 [0.04; 0.78]
WELBECK 2003	176	382	2.1%	0.46 [0.41; 0.51]
SAHOO 2020	43	95	2.0%	0.45 [0.35; 0.56]
YAKOOB 2014	11	38	1.7%	0.29 [0.15; 0.46]
QAZI 2011	0	3	0.4%	0.00 [0.00; 0.71]
GUPTA 2020	8	30	1.6%	0.27 [0.12; 0.46]
CHIABI 2014	6	75	1.6%	0.08 [0.03; 0.17]
CHIOUKH 2018	27	109	2.0%	0.25 [0.17; 0.34]
NEPAL 2020	1	2	0.4%	0.50 [0.01; 0.99]
Total (95% CI)		1892	32.5%	0.28 [0.21; 0.36]
**UMI**				
RUIZ–PELAEZ 2014	43	81	2.0%	0.53 [0.42; 0.64]
REZAEIZADEH 2018	40	85	2.0%	0.47 [0.36; 0.58]
SISWANTO 2018	94	185	2.1%	0.51 [0.43; 0.58]
OSORNO–COVARRUBIAS 2002	11	50	1.8%	0.22 [0.12; 0.36]
SERCE 2014	54	156	2.0%	0.35 [0.27; 0.43]
LI 2019	159	307	2.1%	0.52 [0.46; 0.58]
LUTHULI 2019	14	53	1.8%	0.26 [0.15; 0.40]
MCGREADY 2018	2	132	1.1%	0.02 [0.00; 0.05]
NAVAEI 2010	29	122	2.0%	0.24 [0.17; 0.32]
PIRIYAPOKIN 2020	50	67	1.9%	0.75 [0.63; 0.84]
AFJEH 2013	72	196	2.1%	0.37 [0.30; 0.44]
BASIRI 2015	24	68	1.9%	0.35 [0.24; 0.48]
DE BRITO 2003	30	70	1.9%	0.43 [0.31; 0.55]
DE MELLO 2007	24	51	1.9%	0.47 [0.33; 0.62]
ATASAY 2003	24	37	1.7%	0.65 [0.47; 0.80]
DE ARAÚJO 2007	8	30	1.6%	0.27 [0.12; 0.46]
DECARVALHO 2007	34	108	2.0%	0.31 [0.23; 0.41]
KIDAMBA 2018	5	15	1.3%	0.33 [0.12; 0.62]
JIANG 2020	235	320	2.1%	0.73 [0.68; 0.78]
KULALI 2019	46	165	2.0%	0.28 [0.21; 0.35]
KIRSTEN 2012	231	309	2.1%	0.75 [0.70; 0.80]
KONG 2020	35	50	1.8%	0.70 [0.55; 0.82]
KONG 2016	101	148	2.0%	0.68 [0.60; 0.76]
SUN 2013	7	32	1.6%	0.22 [0.09; 0.40]
NEOCOSUR 2002	43	95	2.0%	0.45 [0.35; 0.56]
BUENOS AIRES 2012	225	426	2.1%	0.53 [0.48; 0.58]
NTULI 2020	9	25	1.6%	0.36 [0.18; 0.57]
GORDANA 2019	141	220	2.1%	0.64 [0.57; 0.70]
WU 2019	1076	2051	2.2%	0.52 [0.50; 0.55]
XU 2019	101	148	2.0%	0.68 [0.60; 0.76]
ZHANG 2011	11	29	1.7%	0.38 [0.21; 0.58]
ZHANG 2020	20	32	1.7%	0.62 [0.44; 0.79]
ZHOU 2014	50	72	1.9%	0.69 [0.57; 0.80]
ZHU 2020	308	441	2.1%	0.70 [0.65; 0.74]
Total (95% CI)		6376	64.2%	0.48 [0.42; 0.53]
**Total (95% CI)**		**8412**	**100.0%**	**0.39 [0.34; 0.44]**

Heterogeneity: Tau2 = 0.4977; Chi2 = 877.88, df = 60 (P < 0.01); I2 = 93%

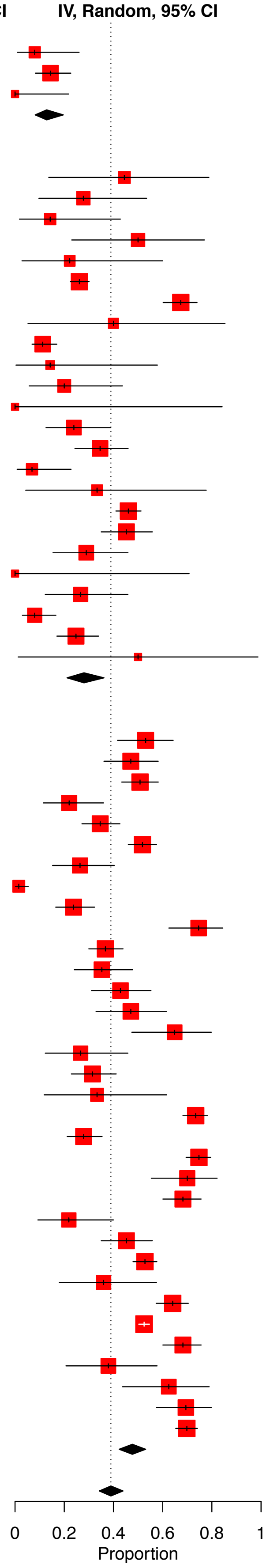

Supplement Figure 5: Primary outcome - survival until discharge for ELGANs analyzed based on geographic region of origin.

Study or Subgroup	Events	Total	Weight	IV, Random, 95% CI
AFRICA				
SEID 2019	2	25	1.0%	0.08 [0.01; 0.26]
SACKEY 2019	141	539	2.1%	0.26 [0.22; 0.30]
OMOIGBERALE 2009	128	190	2.1%	0.67 [0.60; 0.74]
LUTHULI 2019	14	53	1.8%	0.26 [0.15; 0.40]
MUHE 2019	15	104	1.9%	0.14 [0.08; 0.23]
ABDUL−MUMIN 2020	4	20	1.3%	0.20 [0.06; 0.44]
ADINMA 2013	0	2	0.4%	0.00 [0.00; 0.84]
KIDAMBA 2018	5	15	1.3%	0.33 [0.12; 0.62]
KIRSTEN 2012	231	309	2.1%	0.75 [0.70; 0.80]
TAMENE 2020	0	15	0.4%	0.00 [0.00; 0.22]
NTULI 2020	9	25	1.6%	0.36 [0.18; 0.57]
WELBECK 2003	176	382	2.1%	0.46 [0.41; 0.51]
CHIABI 2014	6	75	1.6%	0.08 [0.03; 0.17]
CHIOUKH 2018	27	109	2.0%	0.25 [0.17; 0.34]
Total (95% CI)		1863	21.7%	0.28 [0.18; 0.42]
ASIA				
SEHGAL 2004	4	9	1.1%	0.44 [0.14; 0.79]
PRADHAN 2019	5	18	1.4%	0.28 [0.10; 0.53]
PERVIN 2015	2	14	1.0%	0.14 [0.02; 0.43]
ROY 2006	7	14	1.4%	0.50 [0.23; 0.77]
POUDEL 2010	2	9	0.9%	0.22 [0.03; 0.60]
SISWANTO 2018	94	185	2.1%	0.51 [0.43; 0.58]
LI 2019	159	307	2.1%	0.52 [0.46; 0.58]
MCGREADY 2018	2	132	1.1%	0.02 [0.00; 0.05]
MILES 2017	2	5	0.8%	0.40 [0.05; 0.85]
MUKHOPADHYAY 2013	19	169	1.9%	0.11 [0.07; 0.17]
PIRIYAPOKIN 2020	50	67	1.9%	0.75 [0.63; 0.84]
SAINI 2016	1	7	0.6%	0.14 [0.00; 0.58]
BASU 2008	11	46	1.7%	0.24 [0.13; 0.39]
JIANG 2020	235	320	2.1%	0.73 [0.68; 0.78]
KONG 2020	35	50	1.8%	0.70 [0.55; 0.82]
KONG 2016	101	148	2.0%	0.68 [0.60; 0.76]
SUN 2013	7	32	1.6%	0.22 [0.09; 0.40]
THAKUR 2013	28	81	1.9%	0.35 [0.24; 0.46]
TRAN 2015	2	29	1.0%	0.07 [0.01; 0.23]
UNDELA 2019	2	6	0.9%	0.33 [0.04; 0.78]
WU 2019	1076	2051	2.2%	0.52 [0.50; 0.55]
XU 2019	101	148	2.0%	0.68 [0.60; 0.76]
ZHANG 2011	11	29	1.7%	0.38 [0.21; 0.58]
ZHANG 2020	20	32	1.7%	0.62 [0.44; 0.79]
ZHOU 2014	50	72	1.9%	0.69 [0.57; 0.80]
ZHU 2020	308	441	2.1%	0.70 [0.65; 0.74]
SAHOO 2020	43	95	2.0%	0.45 [0.35; 0.56]
YAKOOB 2014	11	38	1.7%	0.29 [0.15; 0.46]
QAZI 2011	0	3	0.4%	0.00 [0.00; 0.71]
GUPTA 2020	8	30	1.6%	0.27 [0.12; 0.46]
NEPAL 2020	1	2	0.4%	0.50 [0.01; 0.99]
Total (95% CI)		4589	47.2%	0.43 [0.36; 0.50]
EUROPE				
GORDANA 2019	141	220	2.1%	0.64 [0.57; 0.70]
Total (95% CI)		220	2.1%	0.64 [0.58; 0.70]
MIDEAST				
REZAEIZADEH 2018	40	85	2.0%	0.47 [0.36; 0.58]
SERCE 2014	54	156	2.0%	0.35 [0.27; 0.43]
NAVAEI 2010	29	122	2.0%	0.24 [0.17; 0.32]
AFJEH 2013	72	196	2.1%	0.37 [0.30; 0.44]
BASIRI 2015	24	68	1.9%	0.35 [0.24; 0.48]
ATASAY 2003	24	37	1.7%	0.65 [0.47; 0.80]
KULALI 2019	46	165	2.0%	0.28 [0.21; 0.35]
Total (95% CI)		829	13.8%	0.37 [0.30; 0.45]
NAMERICA				
OSORNO−COVARRUBIAS 2002	11	50	1.8%	0.22 [0.12; 0.36]
Total (95% CI)		50	1.8%	0.22 [0.13; 0.36]
SAMERICA				
RUIZ−PELAEZ 2014	43	81	2.0%	0.53 [0.42; 0.64]
DE BRITO 2003	30	70	1.9%	0.43 [0.31; 0.55]
DE MELLO 2007	24	51	1.9%	0.47 [0.33; 0.62]
DE ARAÚJO 2007	8	30	1.6%	0.27 [0.12; 0.46]
DECARVALHO 2007	34	108	2.0%	0.31 [0.23; 0.41]
NEOCOSUR 2002	43	95	2.0%	0.45 [0.35; 0.56]
BUENOS AIRES 2012	225	426	2.1%	0.53 [0.48; 0.58]
Total (95% CI)		861	13.5%	0.44 [0.37; 0.51]
**Total (95% CI)**		**8412**	**100.0%**	**0.39 [0.34; 0.44]**

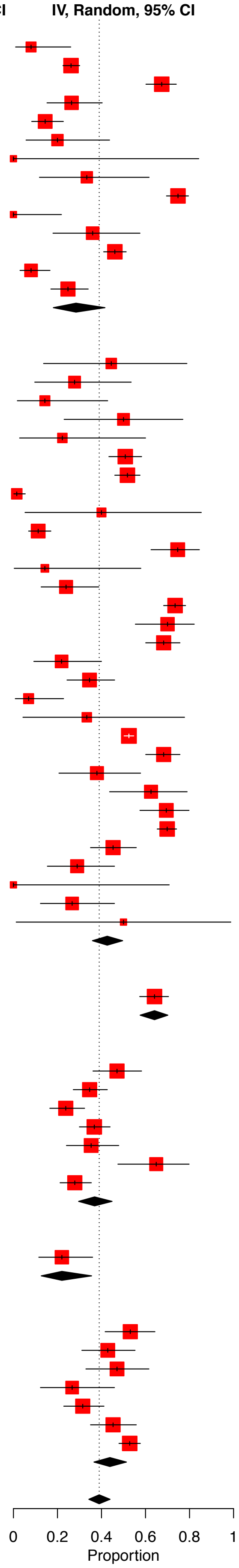

Heterogeneity: Tau2 = 0.4977; Chi2 = 877.88, df = 60 (P < 0.01); I2 = 93%

Supplement Figure 6: Publication bias for the primary outcome - survival until discharge for ELGANs.

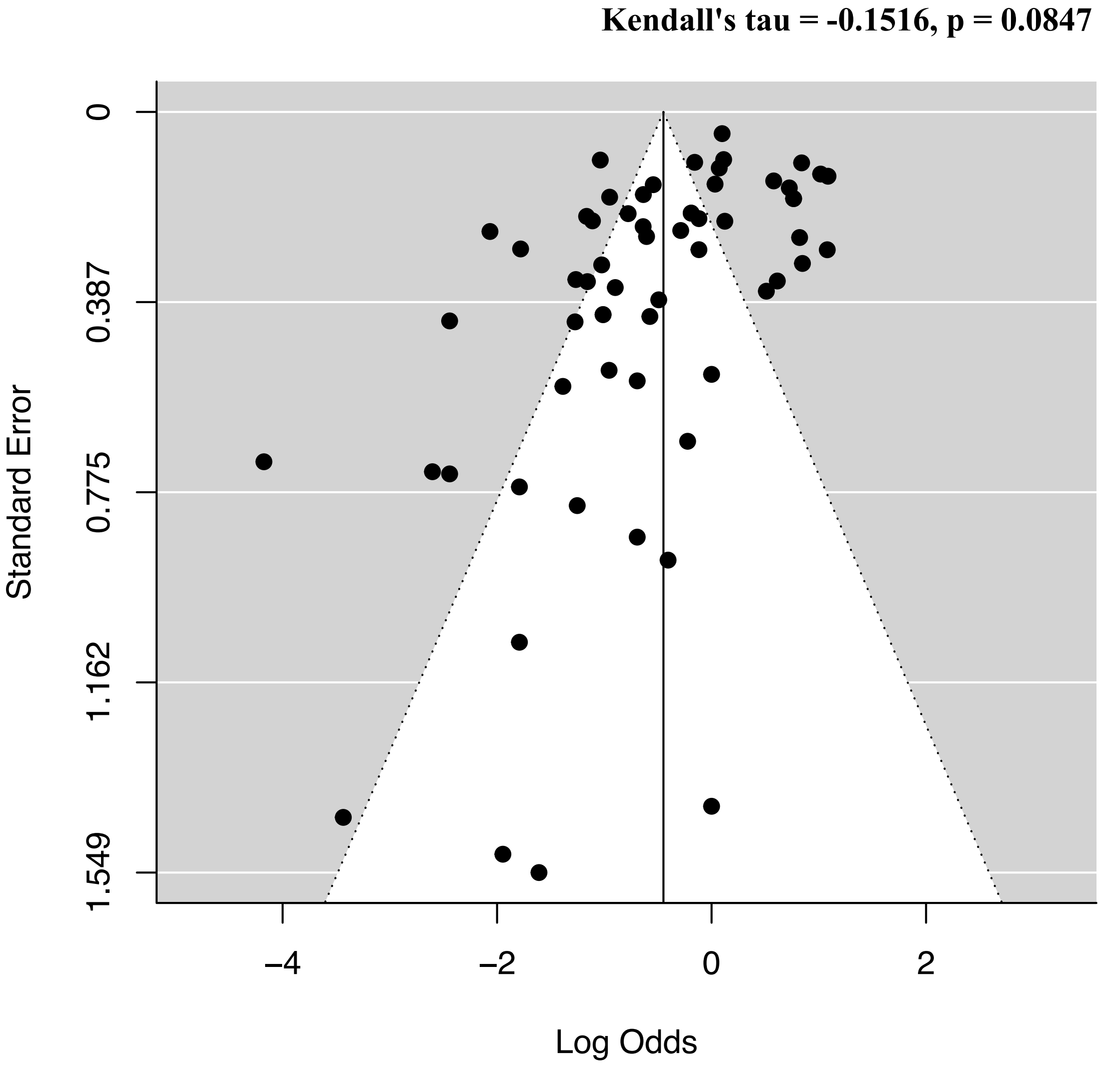

Supplement Figure 7: Secondary outcome - Severe IVH in ELBW neonates.

Study or Subgroup	Events	Total	Weight	IV, Random, 95% CI
CHINA				
LIN 2015	23	178	11.6%	0.13 [0.08; 0.19]
WANG 2012	9	160	10.2%	0.06 [0.03; 0.10]
ZHANG 2020	4	68	8.2%	0.06 [0.02; 0.14]
Total (95% CI)		406	30.0%	0.08 [0.04; 0.15]
INDIA				
MUKHOPADHYAY 2013	53	272	12.2%	0.19 [0.15; 0.25]
Total (95% CI)		272	12.2%	0.19 [0.15; 0.25]
PERU				
ZEA–VERA 2019	13	64	10.6%	0.20 [0.11; 0.32]
Total (95% CI)		64	10.6%	0.20 [0.12; 0.32]
SOUTH AFRICA				
TSHEHLA 2019	4	49	8.1%	0.08 [0.02; 0.20]
Total (95% CI)		49	8.1%	0.08 [0.03; 0.20]
THAILAND				
SRITIPSUKHO 2007	1	21	4.0%	0.05 [0.00; 0.24]
Total (95% CI)		21	4.0%	0.05 [0.01; 0.27]
TURKEY				
ATALAY 2013	7	20	8.7%	0.35 [0.15; 0.59]
CENK CELEBI 2014	12	235	10.8%	0.05 [0.03; 0.09]
KOKSAL 2002	5	12	7.4%	0.42 [0.15; 0.72]
VURAL 2007	5	19	8.1%	0.26 [0.09; 0.51]
Total (95% CI)		286	35.1%	0.22 [0.07; 0.53]
**Total (95% CI)**		**1098**	**100.0%**	**0.14 [0.09; 0.20]**

Heterogeneity: Tau2 = 0.4770; Chi2 = 52.62, df = 10 (P < 0.01); I2 = 81%

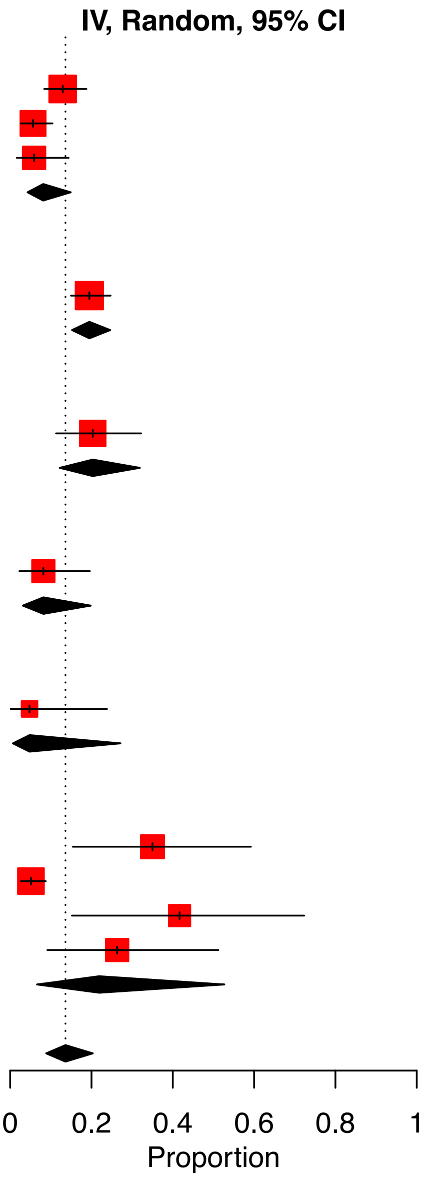

Supplement Figure 8: Publication bias for the secondary outcome - severe IVH in ELBW neonates.

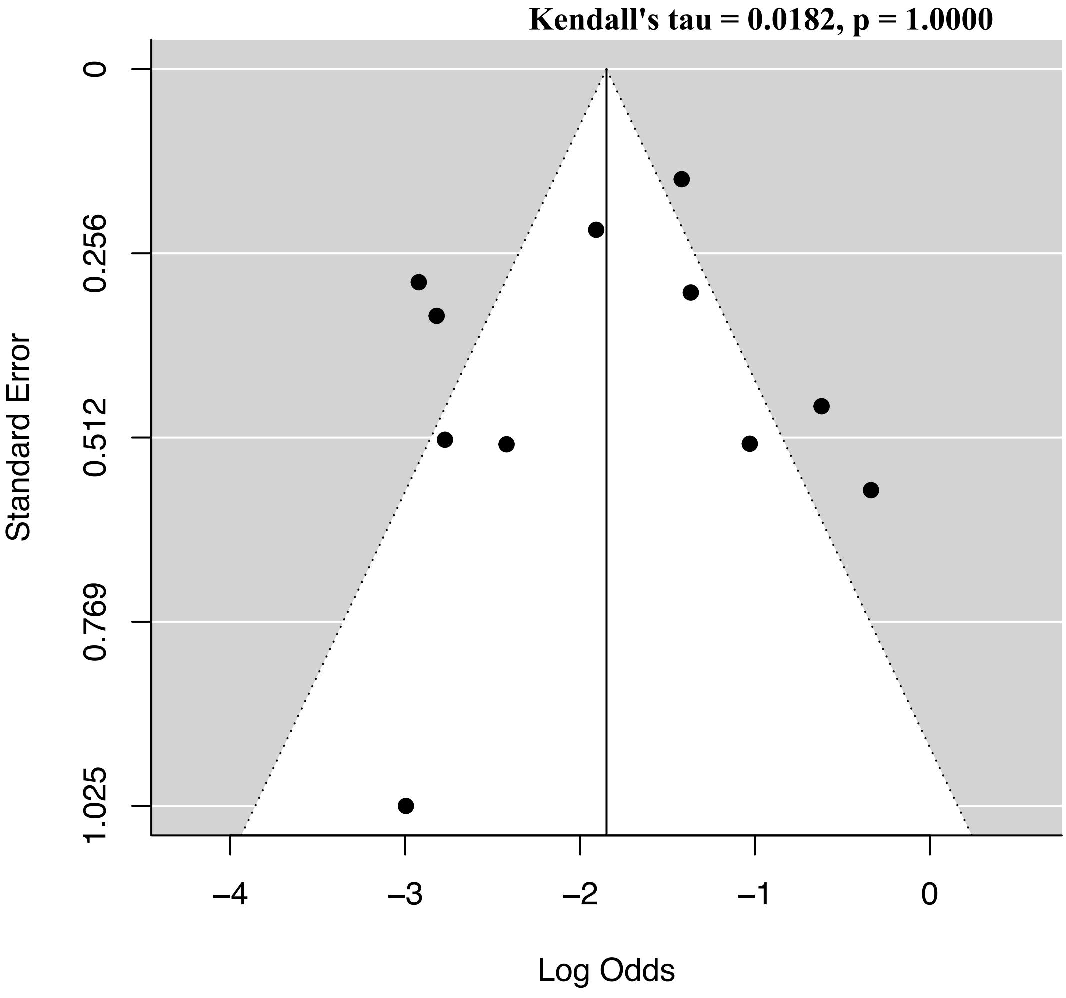

Supplement Figure 9: Secondary outcome - Severe IVH in ELGANs.

Study or Subgroup	Events	Total	Weight	IV, Random, 95% CI	IV, Random, 95% CI
CHINA					
CHEN 2012	13	68	12.6%	0.19 [0.11; 0.30]	
KONG 2020	5	50	8.4%	0.10 [0.03; 0.22]	
WU 2019	190	1431	19.3%	0.13 [0.12; 0.15]	
XU 2019	6	148	9.6%	0.04 [0.02; 0.09]	
ZHOU 2014	18	72	13.7%	0.25 [0.16; 0.37]	
Total (95% CI)		1769	63.6%	0.13 [0.08; 0.20]	
INDIA					
SAHOO 2020	19	95	14.2%	0.20 [0.12; 0.29]	
Total (95% CI)		95	14.2%	0.20 [0.13; 0.29]	
THAILAND					
PIRIYAPOKIN 2020	9	50	10.9%	0.18 [0.09; 0.31]	
Total (95% CI)		50	10.9%	0.18 [0.10; 0.31]	
TURKEY					
KARABULUT 2019	9	87	11.3%	0.10 [0.05; 0.19]	
Total (95% CI)		87	11.3%	0.10 [0.05; 0.19]	
**Total (95% CI)**		**2001**	**100.0%**	**0.14 [0.11; 0.19]**	

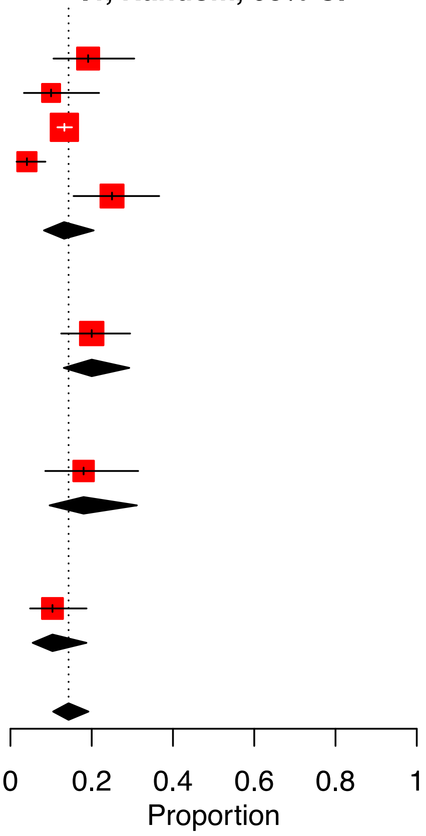

Heterogeneity: Tau2 = 0.1619; Chi2 = 24.56, df = 7 (P < 0.01); I2 = 71%

Supplement Figure 10: Secondary outcome- PVL in ELBW neonates.

Study or Subgroup	Events	Total	Weight	IV, Random, 95% CI
**BRAZIL**				
GOULART 2011	2	36	8.4%	0.06 [0.01; 0.19]
Total (95% CI)		36	8.4%	0.06 [0.01; 0.20]
**CHINA**				
LIN 2015	12	178	20.2%	0.07 [0.04; 0.11]
WANG 2012	2	160	8.7%	0.01 [0.00; 0.04]
ZHANG 2019	3	55	11.0%	0.05 [0.01; 0.15]
ZHANG 2020	4	68	13.0%	0.06 [0.02; 0.14]
Total (95% CI)		461	52.9%	0.05 [0.03; 0.09]
**INDIA**				
OOMMEN 2019	4	30	12.4%	0.13 [0.04; 0.31]
Total (95% CI)		30	12.4%	0.13 [0.05; 0.31]
**PERU**				
ZEA–VERA 2019	5	69	14.4%	0.07 [0.02; 0.16]
Total (95% CI)		69	14.4%	0.07 [0.03; 0.16]
**TURKEY**				
ATALAY 2013	4	20	11.8%	0.20 [0.06; 0.44]
Total (95% CI)		20	11.8%	0.20 [0.08; 0.43]
**Total (95% CI)**		**616**	**100.0%**	**0.07 [0.04; 0.11]**

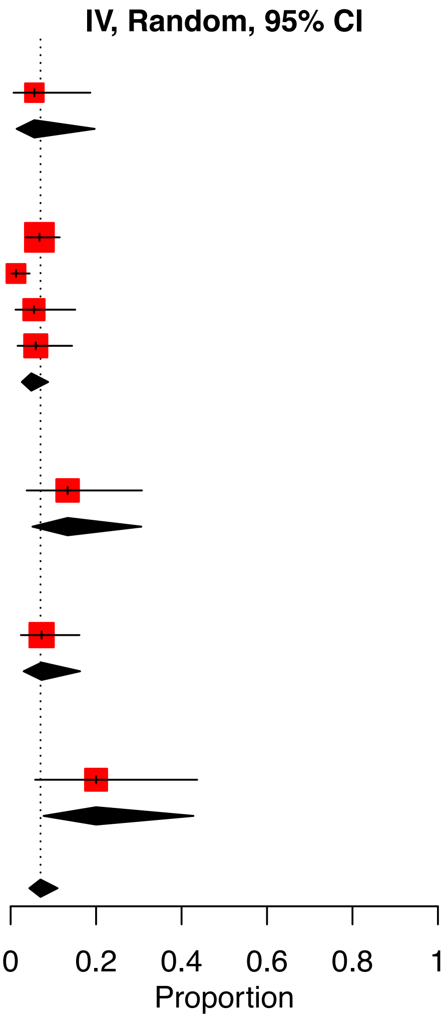

Heterogeneity: Tau2 = 0.2272; Chi2 = 13.12, df = 7 (P = 0.07); I2 = 47%

Supplement Figure 11: Secondary outcome - PVL in ELGANs.

Study or Subgroup	Events	Total	Weight	IV, Random, 95% CI
CHINA				
WU 2019	89	1431	77.5%	0.06 [0.05; 0.08]
ZHANG 2019	4	89	3.5%	0.04 [0.01; 0.11]
ZHOU 2014	6	50	4.9%	0.12 [0.05; 0.24]
CHEN 2012	7	95	6.0%	0.07 [0.03; 0.15]
KONG 2020	2	50	1.8%	0.04 [0.00; 0.14]
Total (95% CI)		1715	93.7%	0.06 [0.05; 0.08]
INDIA				
SAHOO 2020	3	95	2.7%	0.03 [0.01; 0.09]
Total (95% CI)		95	2.7%	0.03 [0.01; 0.09]
TURKEY				
KULALI 2019	4	95	3.6%	0.04 [0.01; 0.10]
Total (95% CI)		95	3.6%	0.04 [0.02; 0.11]
**Total (95% CI)**		**1905**	**100.0%**	**0.06 [0.05; 0.07]**

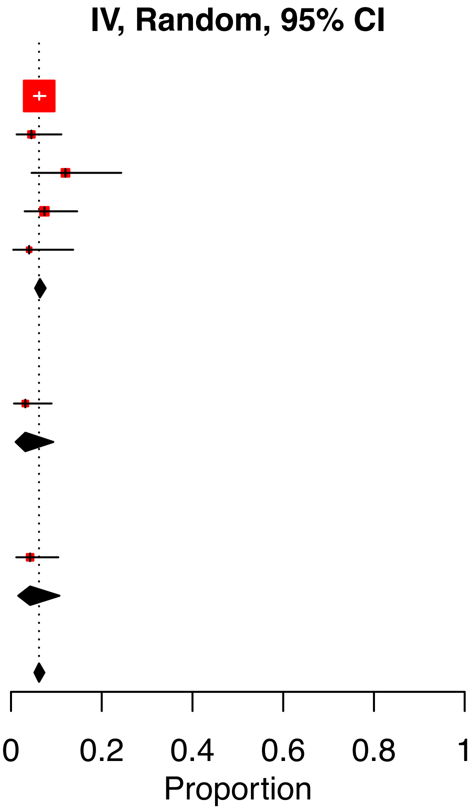

Heterogeneity: Tau2 = 0; Chi2 = 5.93, df = 6 (P = 0.43); I2 = 0%

Supplement Figure 12: Secondary outcome - NDI in ELBW neonates.

Study or Subgroup	Events	Total	IV, Random, 95% CI
INDIA			
OOMMEN 2019	5	30	0.17 [0.06; 0.35]
Total (95% CI)		30	0.17 [0.07; 0.34]

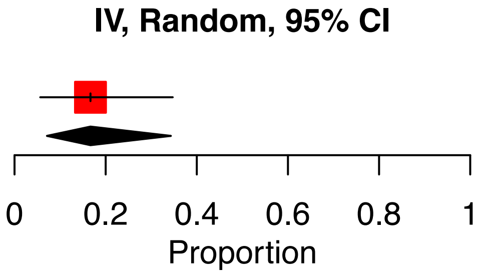

Supplement Figure 13: Secondary outcome- NDI in ELGANs.

Study or Subgroup	Events	Total	Weight	IV, Random, 95% CI
CHINA				
LI 2019	47	131	47.1%	0.36 [0.28; 0.45]
LI 2018	11	46	20.8%	0.24 [0.13; 0.39]
Total (95% CI)		177	68.0%	0.31 [0.21; 0.44]
THAILAND				
PIRIYAPOKIN 2020	9	40	18.0%	0.22 [0.11; 0.38]
Total (95% CI)		40	18.0%	0.23 [0.12; 0.38]
TUNISIA				
CHIOUKH 2018	7	26	14.0%	0.27 [0.12; 0.48]
Total (95% CI)		26	14.0%	0.27 [0.13; 0.47]
**Total (95% CI)**		**243**	**100.0%**	**0.29 [0.23; 0.37]**

IV, Random, 95% CI

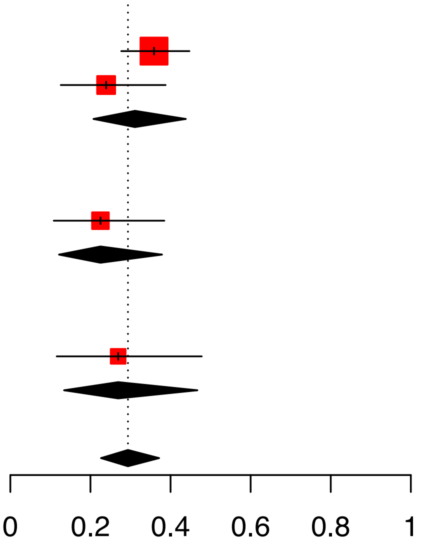

Heterogeneity: Tau2 = 0.0352; Chi2 = 4.05, df = 3 (P = 0.26); I2 = 26%

Supplement Figure 14: Secondary outcome - CP in ELGANs.

Study or Subgroup	Events	Total	Weight	IV, Random, 95% CI
CHINA				
LI 2018	2	79	67.0%	0.03 [0.00; 0.09]
Total (95% CI)		79	67.0%	0.03 [0.01; 0.10]
TUNISIA				
CHIOUKH 2018	1	26	33.0%	0.04 [0.00; 0.20]
Total (95% CI)		26	33.0%	0.04 [0.01; 0.23]
**Total (95% CI)**		**105**	**100.0%**	**0.03 [0.01; 0.09]**

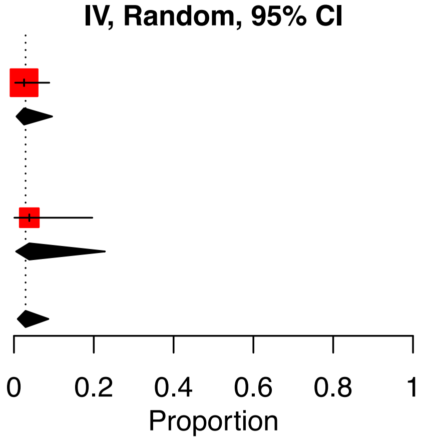

Heterogeneity: Tau2 = 0; Chi2 = 0.12, df = 1 (P = 0.73); I2 = 0%

Supplement Figure 15: Secondary outcome- Any PDA in ELBW neonates.

Study or Subgroup	Events	Total	Weight	IV, Random, 95% CI
BRAZIL				
SOUSA 2017	62	158	9.6%	0.39 [0.32; 0.47]
GOULART 2011	13	36	6.4%	0.36 [0.21; 0.54]
Total (95% CI)		194	16.0%	0.39 [0.32; 0.46]
INDIA				
ROY 2006	6	36	5.0%	0.17 [0.06; 0.33]
MUKHOPADHYAY 2013	74	272	10.0%	0.27 [0.22; 0.33]
THAKUR 2013	66	283	10.0%	0.23 [0.19; 0.29]
Total (95% CI)		591	25.0%	0.25 [0.21; 0.29]
IRAN				
REZAEIZADEH 2018	49	127	9.3%	0.39 [0.30; 0.48]
MOGHADDAM 2015	34	108	8.8%	0.31 [0.23; 0.41]
POURARIAN 2016	18	28	5.7%	0.64 [0.44; 0.81]
Total (95% CI)		263	23.7%	0.42 [0.29; 0.57]
THAILAND				
JIRAPAET 2010	3	5	1.8%	0.60 [0.15; 0.95]
Total (95% CI)		5	1.8%	0.60 [0.20; 0.90]
TURKEY				
SERCE 2014	32	179	9.0%	0.18 [0.13; 0.24]
ATALAY 2013	10	20	5.0%	0.50 [0.27; 0.73]
CENK CELEBI 2014	54	235	9.7%	0.23 [0.18; 0.29]
CETINKAYA 2014	67	197	9.8%	0.34 [0.27; 0.41]
Total (95% CI)		631	33.5%	0.28 [0.19; 0.39]
**Total (95% CI)**		**1684**	**100.0%**	**0.32 [0.26; 0.38]**

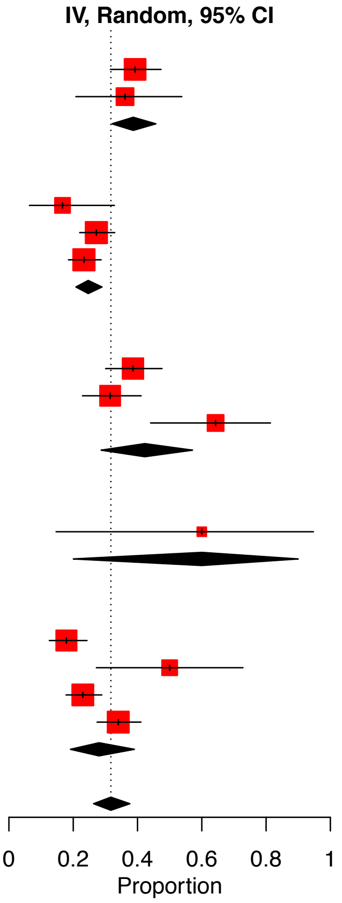

Heterogeneity: Tau2 = 0.1591; Chi2 = 58.92, df = 12 (P < 0.01); I2 = 80%

Supplement Figure 16: Secondary outcome - Any PDA in ELGANs.

Study or Subgroup	Events	Total	Weight	IV, Random, 95% CI
CHINA				
KONG 2016	45	118	34.2%	0.38 [0.29; 0.48]
Total (95% CI)		118	34.2%	0.38 [0.30; 0.47]
IRAN				
REZAEIZADEH 2018	29	85	32.5%	0.34 [0.24; 0.45]
Total (95% CI)		85	32.5%	0.34 [0.25; 0.45]
TURKEY				
SERCE 2014	28	156	33.4%	0.18 [0.12; 0.25]
Total (95% CI)		156	33.4%	0.18 [0.13; 0.25]
**Total (95% CI)**		**359**	**100.0%**	**0.29 [0.18; 0.44]**

Heterogeneity: Tau2 = 0.2760; Chi2 = 14.71, df = 2 (P < 0.01); I2 = 86%

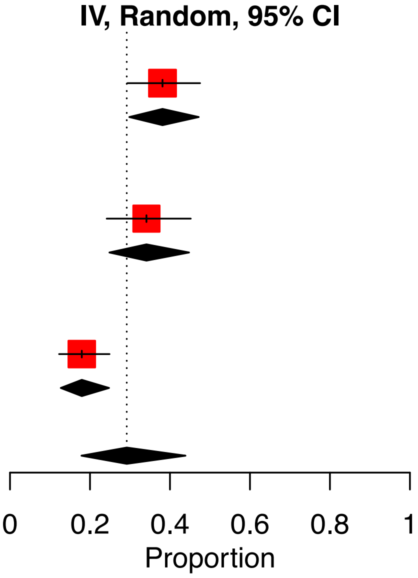

Supplement Figure 17: Publication bias for the secondary outcome - Any PDA in ELBW neonates.

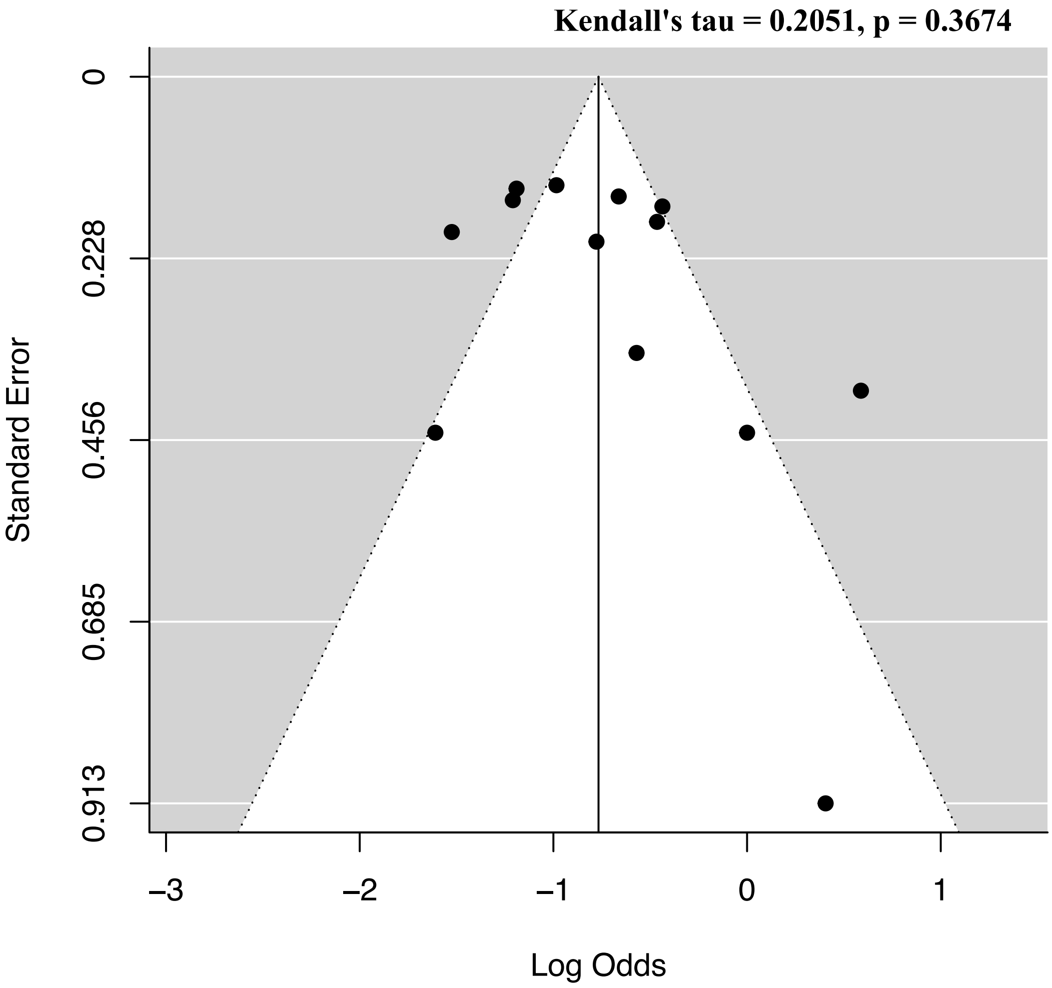

Supplement Figure 18: Secondary outcome- PDA requiring intervention in ELBW neonates.

Study or Subgroup	Events	Total	Weight	IV, Random, 95% CI
CHINA				
WANG 2012	10	160	25.5%	0.06 [0.03; 0.11]
ZHANG 2020	21	68	26.6%	0.31 [0.20; 0.43]
LIN 2015	54	206	28.2%	0.26 [0.20; 0.33]
Total (95% CI)		434	80.3%	0.18 [0.08; 0.37]
INDIA				
SEHGAL 2004	3	52	19.7%	0.06 [0.01; 0.16]
Total (95% CI)		52	19.7%	0.06 [0.02; 0.16]
**Total (95% CI)**		**486**	**100.0%**	**0.15 [0.07; 0.30]**

Heterogeneity: Tau2 = 0.7410; Chi2 = 31.24, df = 3 (P < 0.01); I2=90%

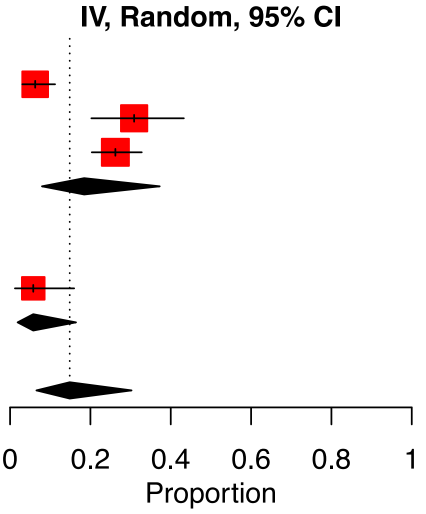

Supplement Figure 19: Secondary outcome - PDA requiring intervention in ELGANs.

Study or Subgroup	Events	Total	Weight	IV, Random, 95% CI
CHINA				
ZHOU 2014	51	72	19.5%	0.71 [0.59; 0.81]
KONG 2020	17	50	18.8%	0.34 [0.21; 0.49]
Total (95% CI)		122	38.3%	0.53 [0.20; 0.84]
INDIA				
SAHOO 2020	31	95	20.3%	0.33 [0.23; 0.43]
Total (95% CI)		95	20.3%	0.33 [0.24; 0.43]
TURKEY				
KARABULUT 2019	87	206	21.4%	0.42 [0.35; 0.49]
KULALI 2019	61	88	20.0%	0.69 [0.59; 0.79]
Total (95% CI)		294	41.4%	0.56 [0.30; 0.79]
**Total (95% CI)**		**511**	**100.0%**	**0.50 [0.35; 0.65]**

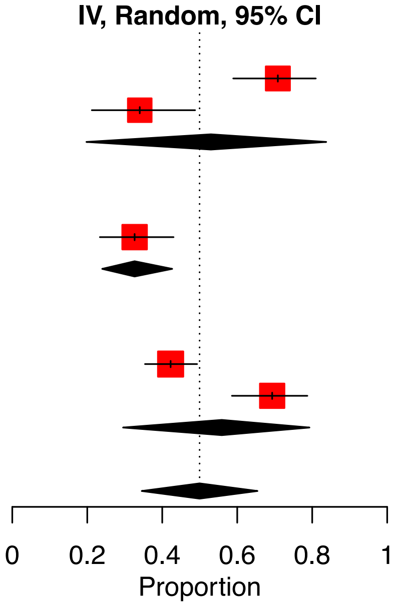

Heterogeneity: Tau = 0.4757; Chi2 = 44.14, df = 4 (P < 0.01); I = 91%

Supplement Figure 20: Secondary outcome- Requirement of invasive mechanical ventilation in ELBW neonates.

Study or Subgroup	Events	Total	Weight	IV, Random, 95% CI
BRAZIL				
GOULART 2011	28	36	15.7%	0.78 [0.61; 0.90]
Total (95% CI)		36	15.7%	0.78 [0.64; 0.91]
CHINA				
WANG 2012	115	160	18.0%	0.72 [0.64; 0.79]
ZHANG 2020	58	68	17.5%	0.85 [0.75; 0.93]
Total (95% CI)		228	35.5%	0.78 [0.65; 0.92]
INDIA				
SEHGAL 2004	25	52	15.7%	0.48 [0.34; 0.62]
Total (95% CI)		52	15.7%	0.48 [0.34; 0.62]
IRAN				
AFJEH 2017	83	156	17.7%	0.53 [0.45; 0.61]
Total (95% CI)		156	17.7%	0.53 [0.45; 0.61]
JAMAICA				
TROTMAN 2007	21	47	15.4%	0.45 [0.30; 0.60]
Total (95% CI)		47	15.4%	0.45 [0.30; 0.59]
**Total (95% CI)**		**519**	**100.0%**	**0.64 [0.51; 0.77]**

Heterogeneity: $Tau^2 = 0.0230$; $Chi^2 = 51.27$, df = 5 ($P < 0.01$); $I^2 = 90\%$

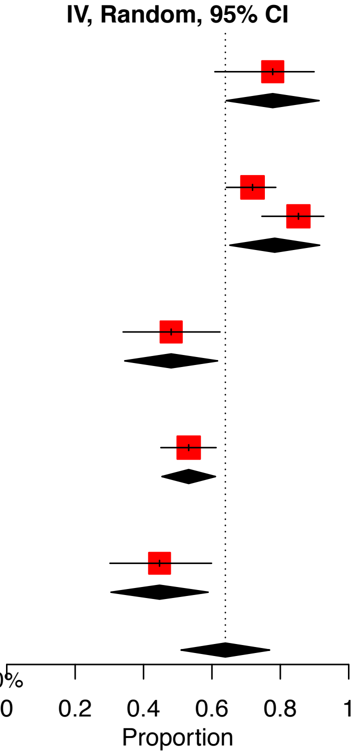

Supplement Figure 21: Secondary outcome - Requirement of invasive mechanical ventilation in ELGANs.

Study or Subgroup	Events	Total	Weight	IV, Random, 95% CI
CHINA				
WU 2019	1603	2051	15.3%	0.78 [0.76; 0.80]
XU 2019	83	148	14.1%	0.56 [0.48; 0.64]
ZHOU 2014	62	72	14.1%	0.86 [0.76; 0.93]
LI 2019	102	131	14.4%	0.78 [0.70; 0.85]
Total (95% CI)		2402	58.0%	0.75 [0.65; 0.84]
INDIA				
SAHOO 2020	67	95	13.8%	0.71 [0.60; 0.79]
Total (95% CI)		95	13.8%	0.71 [0.61; 0.80]
IRAN				
AFJEH 2017	111	203	14.5%	0.55 [0.48; 0.62]
Total (95% CI)		203	14.5%	0.55 [0.48; 0.62]
TUNISIA				
CHIOUKH 2018	45	109	13.8%	0.41 [0.32; 0.51]
Total (95% CI)		109	13.8%	0.41 [0.32; 0.51]
**Total (95% CI)**		**2809**	**100.0%**	**0.67 [0.56; 0.77]**

Heterogeneity: $Tau^2 = 0.0191$; $Chi^2 = 127.37$, df = 6 ($P < 0.01$); $I^2 = 95\%$

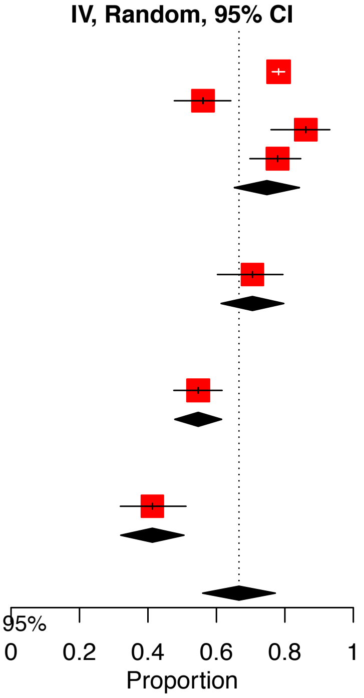

Supplement Figure 22: Secondary outcome- BPD in ELBW neonates

Study or Subgroup	Events	Total	Weight	IV, Random, 95% CI
BRAZIL				
GOULART 2011	8	36	10.3%	0.22 [0.10; 0.39]
Total (95% CI)		36	10.3%	0.22 [0.09; 0.36]
CHINA				
ZHANG 2020	32	68	10.9%	0.47 [0.35; 0.60]
LIN 2015	64	133	12.0%	0.48 [0.39; 0.57]
CHEN 2019	60	115	11.8%	0.52 [0.43; 0.62]
Total (95% CI)		316	34.6%	0.49 [0.44; 0.55]
INDIA				
BHUNWAL 2018	8	34	10.0%	0.24 [0.11; 0.41]
Total (95% CI)		34	10.0%	0.24 [0.09; 0.38]
SERBIA				
GORDANA 2019	48	89	11.4%	0.54 [0.43; 0.65]
Total (95% CI)		89	11.4%	0.54 [0.44; 0.64]
SOUTH AFRICA				
TSHEHLA 2019	6	36	10.8%	0.17 [0.06; 0.33]
Total (95% CI)		36	10.8%	0.17 [0.04; 0.29]
THAILAND				
SRITIPSUKHO 2007	6	11	5.5%	0.55 [0.23; 0.83]
Total (95% CI)		11	5.5%	0.55 [0.25; 0.84]
TURKEY				
SERCE 2014	22	56	10.5%	0.39 [0.26; 0.53]
ATALAY 2013	6	16	6.9%	0.38 [0.15; 0.65]
Total (95% CI)		72	17.5%	0.39 [0.28; 0.50]
**Total (95% CI)**		**594**	**100.0%**	**0.39 [0.30; 0.48]**

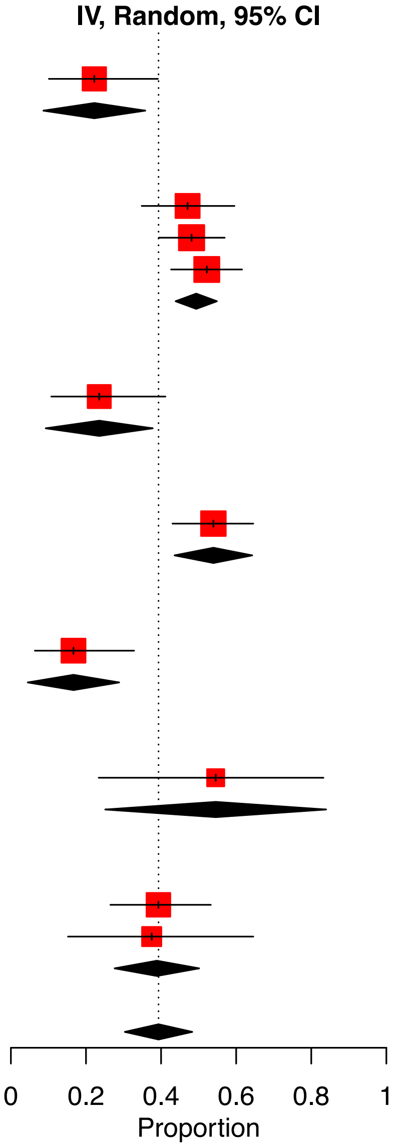

Heterogeneity Tau2= 0.1805; Chi2 = 30.00, df = 9 (P < 0.01); I2 = 70%

Supplement Figure 23: Publication bias for the secondary outcome - BPD in ELBW neonates.

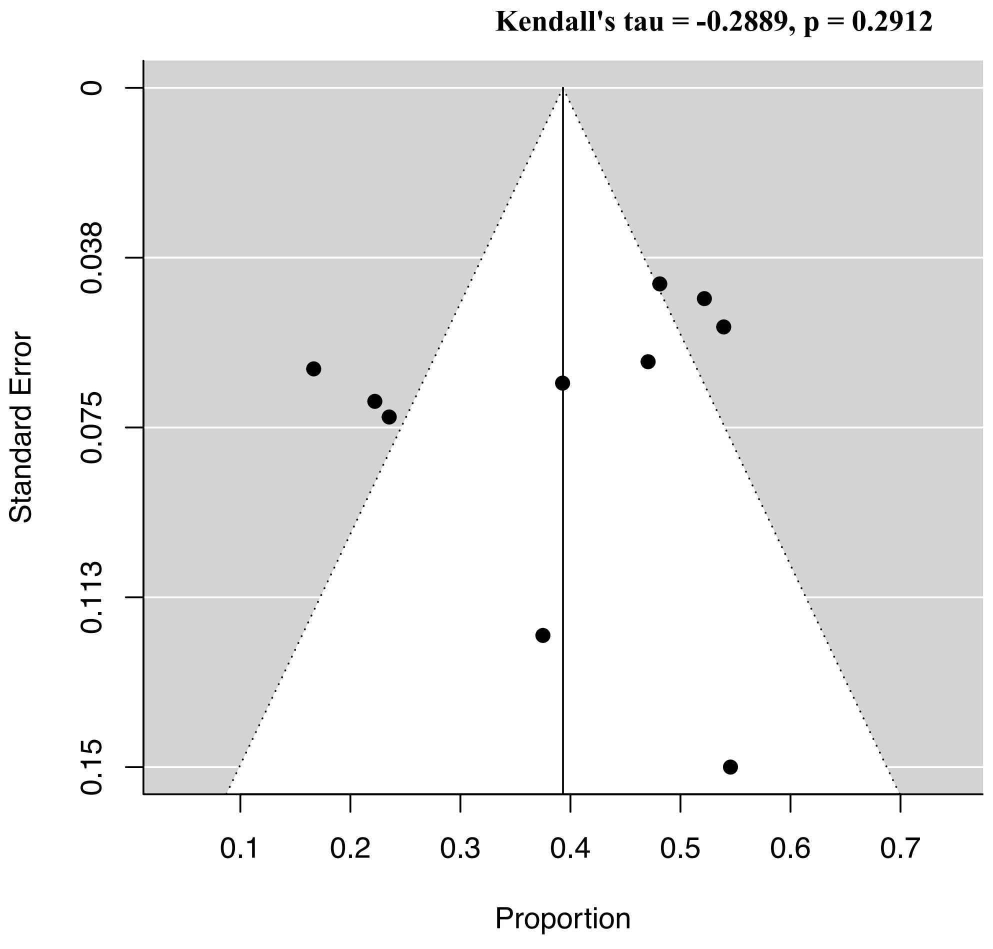

Supplement Figure 24: Secondary outcome- BPD in ELGANs

Study or Subgroup	Events	Total	Weight	IV, Random, 95% CI
CHINA				
WU 2019	374	1158	9.0%	0.32 [0.30; 0.35]
XU 2019	15	101	7.7%	0.15 [0.09; 0.23]
ZHOU 2014	9	58	7.0%	0.16 [0.07; 0.27]
JIANG 2020	144	235	8.7%	0.61 [0.55; 0.68]
KONG 2020	6	35	6.2%	0.17 [0.07; 0.34]
KONG 2016	35	101	8.3%	0.35 [0.25; 0.45]
LI 2019	60	131	8.5%	0.46 [0.37; 0.55]
Total (95% CI)		1819	55.4%	0.31 [0.21; 0.43]
INDIA				
BHUNWAL 2018	7	16	5.7%	0.44 [0.20; 0.70]
SAHOO 2020	26	43	7.4%	0.60 [0.44; 0.75]
Total (95% CI)		59	13.2%	0.55 [0.40; 0.70]
SERBIA				
GORDANA 2019	76	141	8.5%	0.54 [0.45; 0.62]
Total (95% CI)		141	8.5%	0.54 [0.46; 0.62]
TURKEY				
SERCE 2014	16	54	7.6%	0.30 [0.18; 0.44]
KARABULUT 2019	21	87	7.9%	0.24 [0.16; 0.35]
KULALI 2019	31	45	7.3%	0.69 [0.53; 0.82]
Total (95% CI)		186	22.9%	0.40 [0.17; 0.67]
**Total (95% CI)**		**2205**	**100.0%**	**0.37 [0.29; 0.47]**

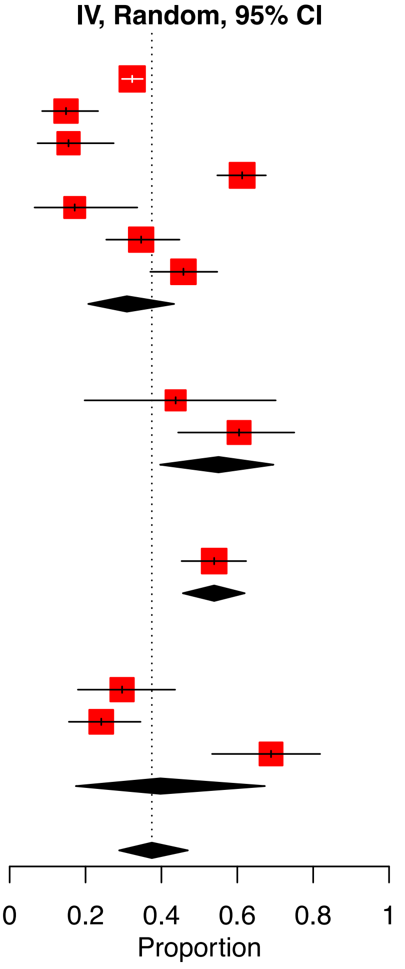

Heterogeneity: Tau2 = 0.4367; Chi2 = 154.74, df = 12 (P < 0.01); I2 = 92%

Supplement Figure 25: Publication bias for the secondary outcome - BPD in ELGANs.

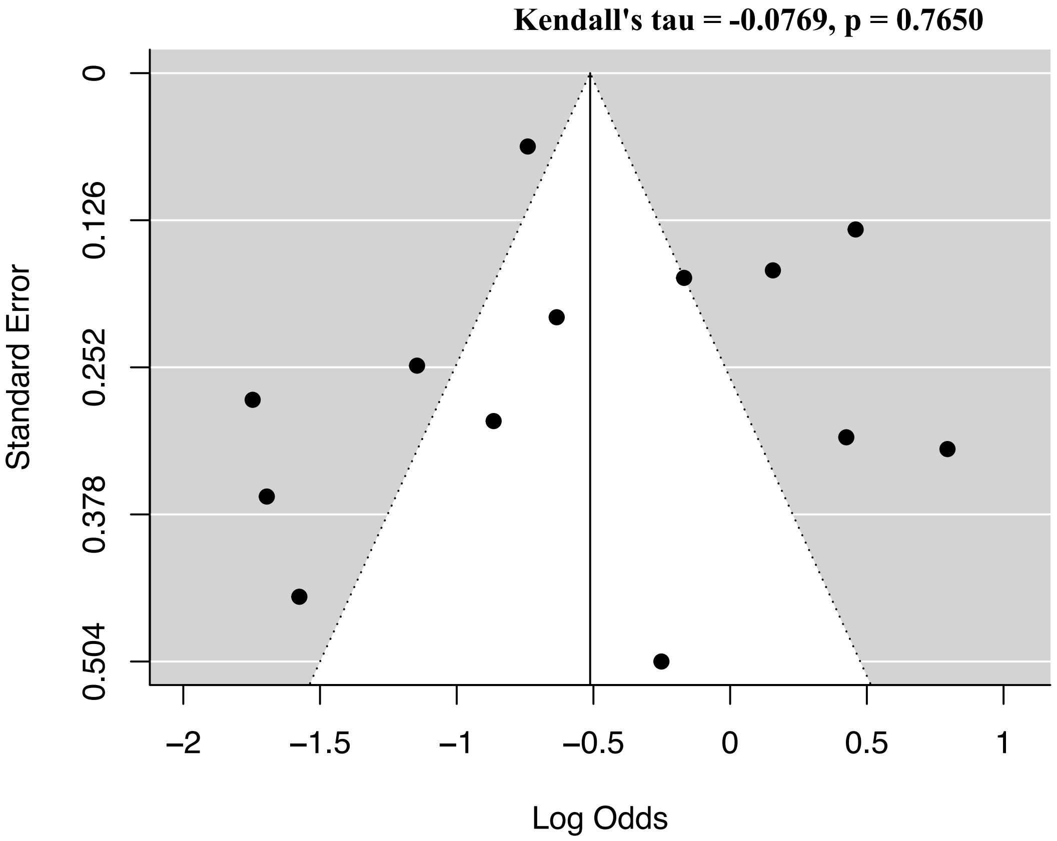

Supplement Figure 26: Secondary outcome- Any sepsis in ELBW neonates

Study or Subgroup	Events	Total	Weight	IV, Random, 95% CI
BRAZIL				
SOUSA 2017	51	158	9.3%	0.32 [0.25; 0.40]
GOULART 2011	27	36	7.5%	0.75 [0.58; 0.88]
Total (95% CI)		194	16.8%	0.54 [0.16; 0.88]
CHINA				
WANG 2012	27	160	9.0%	0.17 [0.11; 0.24]
Total (95% CI)		160	9.0%	0.17 [0.12; 0.23]
INDIA				
OOMMEN 2019	5	30	6.5%	0.17 [0.06; 0.35]
UNDELA 2019	7	10	4.9%	0.70 [0.35; 0.93]
Total (95% CI)		40	11.4%	0.39 [0.06; 0.88]
IRAN				
REZAEIZADEH 2018	37	127	9.1%	0.29 [0.21; 0.38]
MOGHADDAM 2015	13	108	8.3%	0.12 [0.07; 0.20]
Total (95% CI)		235	17.4%	0.20 [0.08; 0.42]
NIGERIA				
OGUNLESI 2011	37	78	8.9%	0.47 [0.36; 0.59]
Total (95% CI)		78	8.9%	0.47 [0.37; 0.58]
SOUTH AFRICA				
TSHEHLA 2019	42	142	9.2%	0.30 [0.22; 0.38]
Total (95% CI)		142	9.2%	0.30 [0.23; 0.38]
TURKEY				
SERCE 2014	93	179	9.4%	0.52 [0.44; 0.59]
BOLAT 2012	33	59	8.6%	0.56 [0.42; 0.69]
CETINKAYA 2014	90	197	9.4%	0.46 [0.39; 0.53]
Total (95% CI)		435	27.4%	0.50 [0.44; 0.55]
**Total (95% CI)**		**1284**	**100.0%**	**0.37 [0.28; 0.48]**

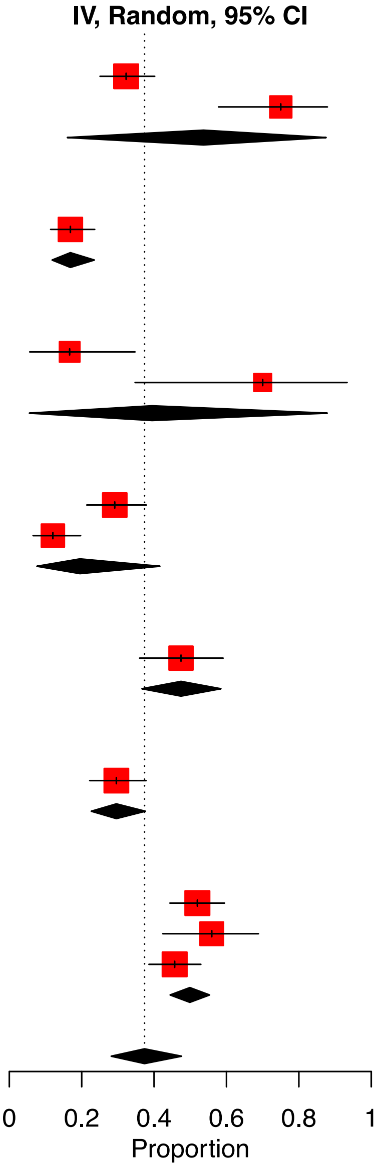

Heterogeneity: Tau2 = 0.4660; Chi2 = 119.55, df = 11 (P < 0.01); I2 = 91%

Supplement Figure 27: Publication bias for the secondary outcome - Any sepsis in ELBW neonates.

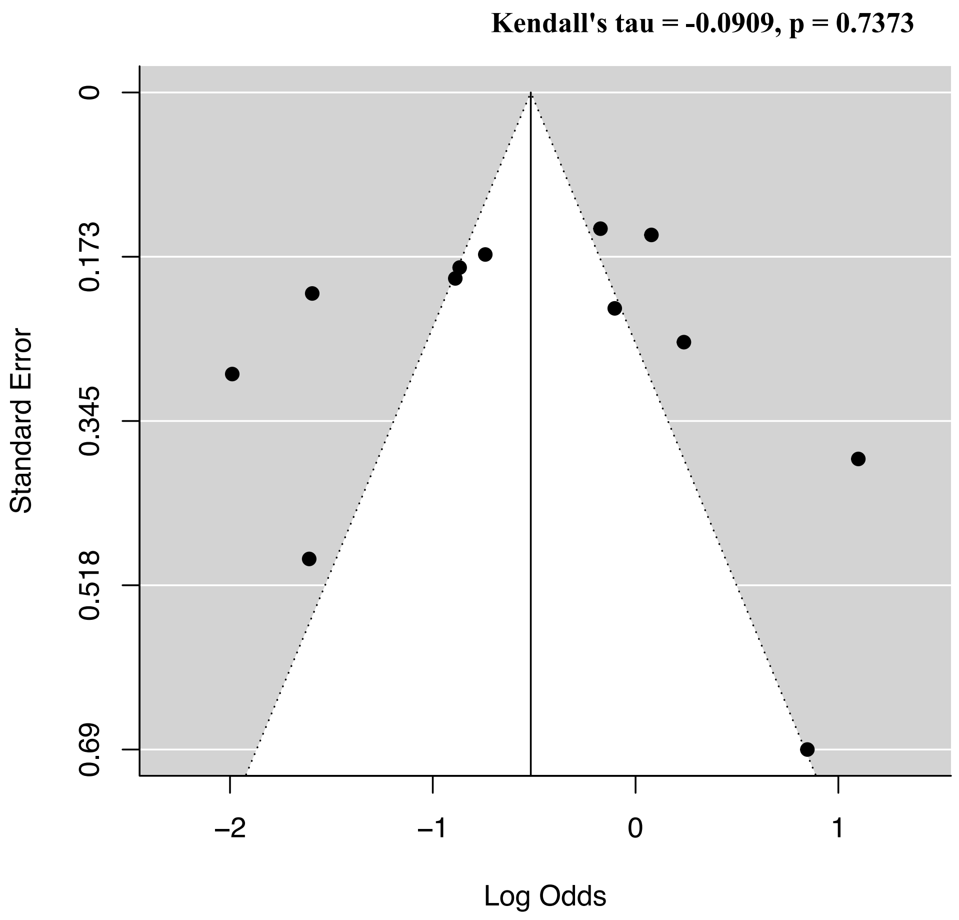

Supplement Figure 28: Secondary outcome- Any sepsis in ELGANs

Study or Subgroup	Events	Total	Weight	IV, Random, 95% CI
CHINA				
XU 2019	20	148	15.6%	0.14 [0.08; 0.20]
ZHANG 2020	12	68	14.8%	0.18 [0.09; 0.29]
ZHOU 2014	60	72	14.8%	0.83 [0.73; 0.91]
JIANG 2020	112	320	16.5%	0.35 [0.30; 0.41]
Total (95% CI)		608	61.7%	0.35 [0.14; 0.64]
INDIA				
UNDELA 2019	5	6	6.5%	0.83 [0.36; 1.00]
Total (95% CI)		6	6.5%	0.83 [0.37; 0.98]
IRAN				
REZAEIZADEH 2018	27	85	15.7%	0.32 [0.22; 0.43]
Total (95% CI)		85	15.7%	0.32 [0.23; 0.42]
TURKEY				
SERCE ı 2014	73	156	16.2%	0.47 [0.39; 0.55]
Total (95% CI)		156	16.2%	0.47 [0.39; 0.55]
**Total (95% CI)**		**855**	**100.0%**	**0.40 [0.25; 0.57]**

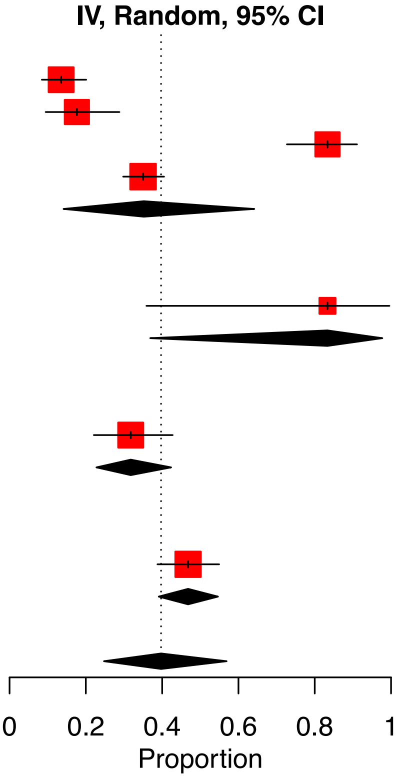

Heterogeneity: Tau2 = 0.7490; Chi2 = 97.81, df = 6 (P < 0.01); I2 = 94%

Supplement Figure 29: Secondary outcome- Culture proven sepsis in ELBW neonates

Study or Subgroup	Events	Total	Weight	IV, Random, 95% CI
CHINA				
LIN 2015	45	206	10.9%	0.22 [0.16; 0.28]
Total (95% CI)		206	10.9%	0.22 [0.17; 0.28]
INDIA				
SEHGAL 2004	7	52	7.4%	0.13 [0.06; 0.26]
ROY 2006	9	36	7.8%	0.25 [0.12; 0.42]
MUKHOPADHYAY 2013	112	272	11.5%	0.41 [0.35; 0.47]
THAKURa 2013	95	283	11.4%	0.34 [0.28; 0.39]
Total (95% CI)		643	38.1%	0.30 [0.21; 0.40]
MALAYSIA				
HO 2001	30	136	10.4%	0.22 [0.15; 0.30]
Total (95% CI)		136	10.4%	0.22 [0.16; 0.30]
NIGERIA				
OGUNLESI 2011	28	78	10.0%	0.36 [0.25; 0.48]
Total (95% CI)		78	10.0%	0.36 [0.26; 0.47]
SOUTH AFRICA				
TSHEHLA 2019	19	142	9.8%	0.13 [0.08; 0.20]
Total (95% CI)		142	9.8%	0.13 [0.09; 0.20]
THAILAND				
JIRAPAET 2010	3	5	2.9%	0.60 [0.15; 0.95]
Total (95% CI)		5	2.9%	0.60 [0.20; 0.90]
TURKEY				
ATALAY 2013	12	20	6.8%	0.60 [0.36; 0.81]
CENK CELEBI 2014	48	235	11.0%	0.20 [0.15; 0.26]
Total (95% CI)		255	17.8%	0.37 [0.09; 0.77]
**Total (95% CI)**		**1465**	**100.0%**	**0.28 [0.21; 0.35]**

IV, Random, 95% CI

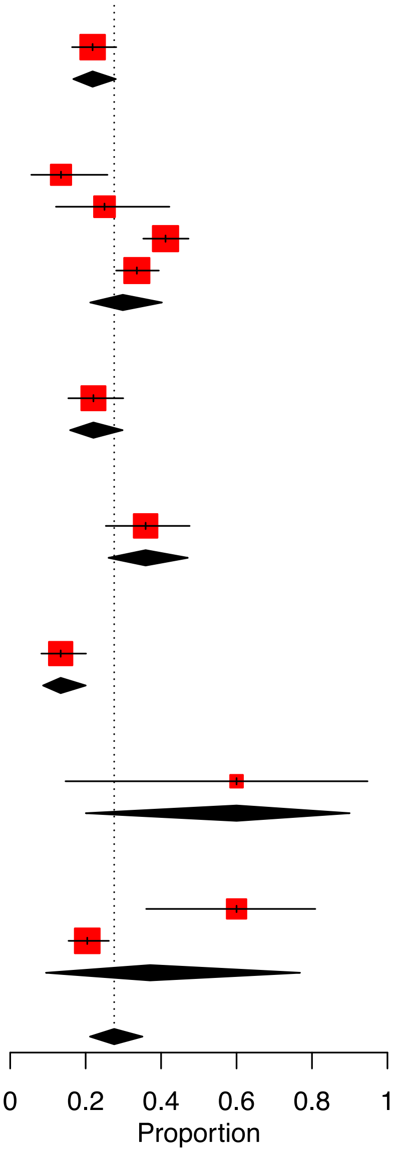

Heterogeneity: Tau2 = 0.2632; Chi2 = 71.85, df = 10 (P < 0.01); I2 = 86%

Supplement Figure 30: Publication bias for the secondary outcome - Culture proven sepsis in ELBW neonates.

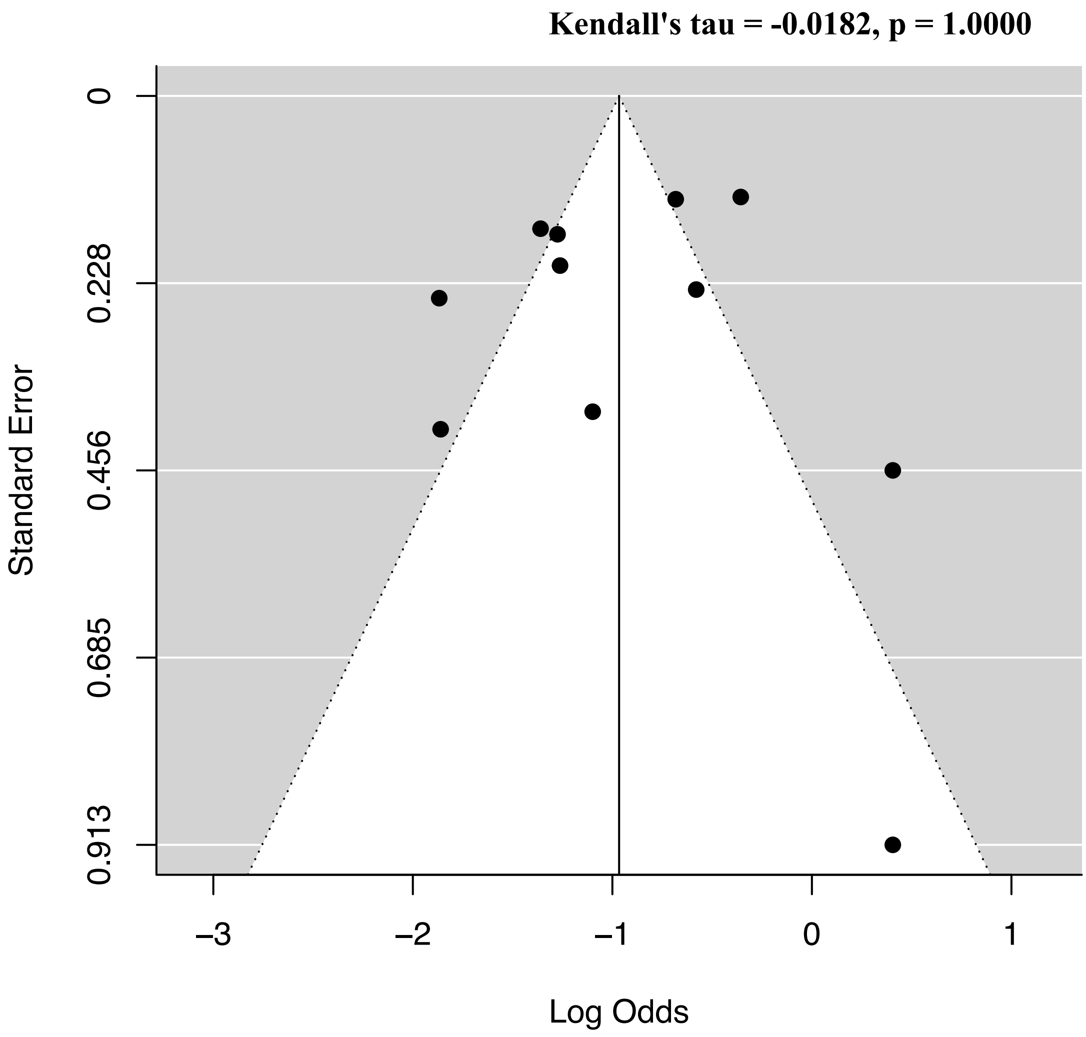

Supplement Figure 31: Secondary outcome- Culture proven sepsis in ELGANs

Study or Subgroup	Events	Total	Weight	IV, Random, 95% CI
CHINA				
WU 2019	250	1588	15.8%	0.16 [0.14; 0.18]
KONG 2020	5	50	11.5%	0.10 [0.03; 0.22]
KONG 2016	23	148	14.6%	0.16 [0.10; 0.22]
Total (95% CI)		1786	41.8%	0.16 [0.14; 0.17]
INDIA				
SAHOO 2020	20	95	14.3%	0.21 [0.13; 0.31]
Total (95% CI)		95	14.3%	0.21 [0.14; 0.30]
MALAYSIA				
HO 2001	46	168	15.1%	0.27 [0.21; 0.35]
Total (95% CI)		168	15.1%	0.27 [0.21; 0.35]
TURKEY				
KARABULUT 2019	11	87	13.5%	0.13 [0.06; 0.21]
KULALI 2019	85	165	15.2%	0.52 [0.44; 0.59]
Total (95% CI)		252	28.7%	0.29 [0.05; 0.74]
**Total (95% CI)**		**2301**	**100.0%**	**0.21 [0.12; 0.32]**

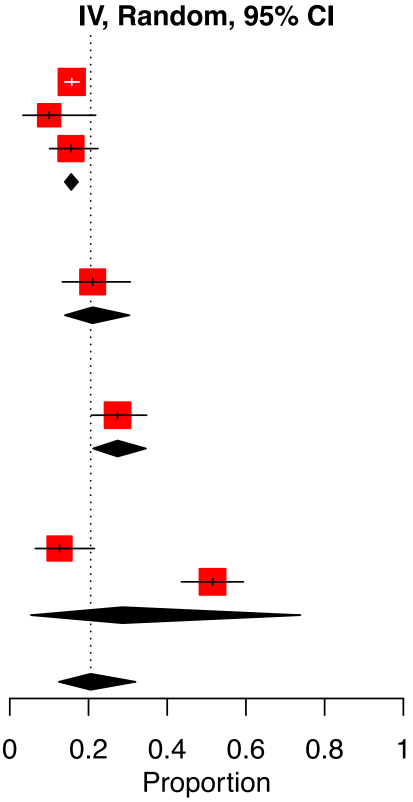

Heterogeneity: Tau = 0.5827; Chi = 117.49, df = 6 (P < 0.01); I = 95%

Supplement Figure 32: Secondary outcome- NEC in ELBW neonates

Study or Subgroup	Events	Total	Weight	IV, Random, 95% CI
**BRAZIL**				
SOUSA 2017	12	158	7.8%	0.08 [0.04; 0.13]
GOULART 2011	2	36	1.9%	0.06 [0.01; 0.19]
Total (95% CI)		194	9.7%	0.07 [0.04; 0.12]
**CHINA**				
WANG 2012	8	160	6.0%	0.05 [0.02; 0.10]
ZHANG 2020	5	68	4.1%	0.07 [0.02; 0.16]
LIN 2015	12	206	7.9%	0.06 [0.03; 0.10]
Total (95% CI)		434	18.0%	0.06 [0.04; 0.08]
**INDIA**				
SEHGAL 2004	3	52	2.7%	0.06 [0.01; 0.16]
MUKHOPADHYAY 2013	32	272	12.7%	0.12 [0.08; 0.16]
THAKUR 2013	26	283	11.8%	0.09 [0.06; 0.13]
Total (95% CI)		607	27.2%	0.10 [0.08; 0.13]
**IRAN**				
REZAEIZADEH 2018	12	127	7.7%	0.09 [0.05; 0.16]
Total (95% CI)		127	7.7%	0.09 [0.05; 0.16]
**MALAYSIA**				
BOO 2012	98	1051	17.7%	0.09 [0.08; 0.11]
Total (95% CI)		1051	17.7%	0.09 [0.08; 0.11]
**SOUTH AFRICA**				
TSHEHLA 2019	0	66	0.5%	0.00 [0.00; 0.05]
Total (95% CI)		66	0.5%	0.01 [0.00; 0.11]
**THAILAND**				
SRITIPSUKHO 2007	1	21	1.0%	0.05 [0.00; 0.24]
Total (95% CI)		21	1.0%	0.05 [0.01; 0.27]
**TURKEY**				
SERCE 2014	23	179	10.9%	0.13 [0.08; 0.19]
CENK CELEBI 2014	11	235	7.5%	0.05 [0.02; 0.08]
Total (95% CI)		414	18.4%	0.08 [0.03; 0.20]
**Total (95% CI)**		**2914**	**100.0%**	**0.08 [0.07; 0.10]**

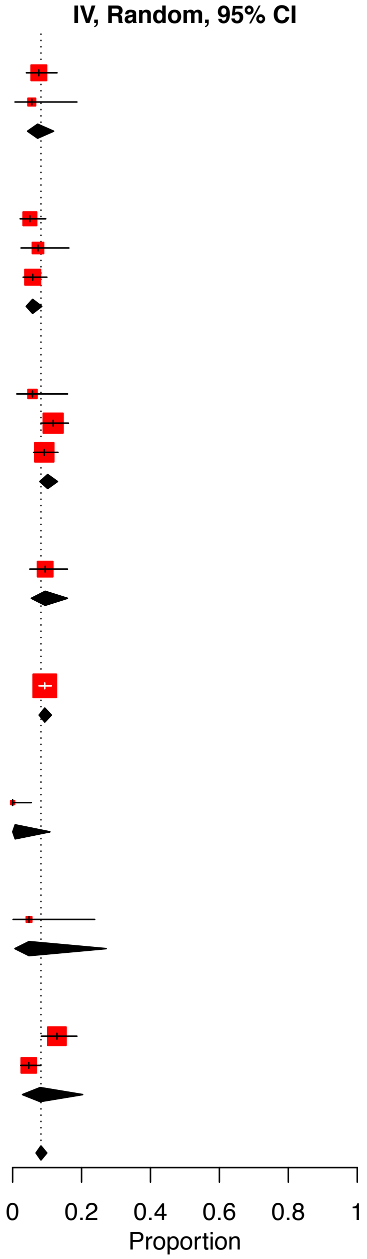

Heterogeneity: Tau2 = 0.0508; Chi2 = 21.99, df = 13 (P = 0.06); I2 = 41%

Supplement Figure 33: Publication bias for the secondary outcome - NEC in ELBW neonates.

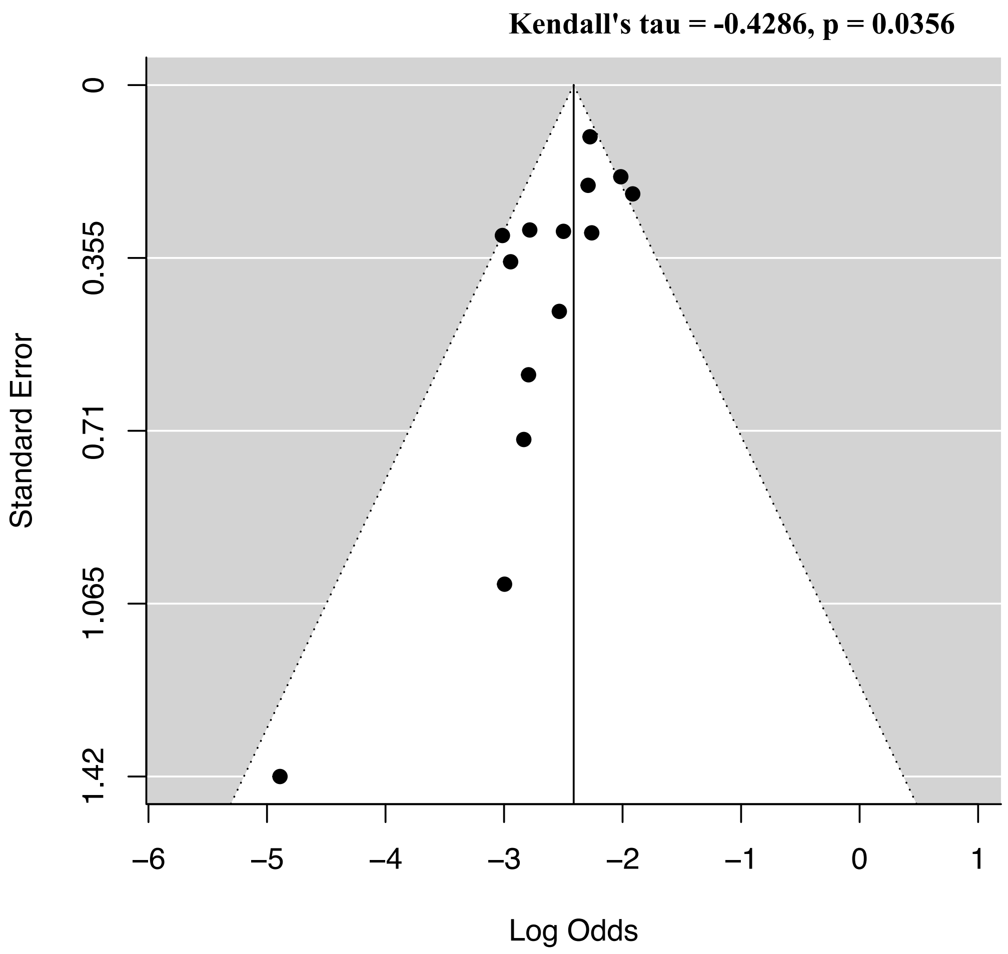

Supplement Figure 34: Secondary outcome- NEC in ELGANs

Study or Subgroup	Events	Total	Weight	IV, Random, 95% CI
CHINA				
WU 2019	160	1588	23.2%	0.10 [0.09; 0.12]
XU 2019	8	148	5.0%	0.05 [0.02; 0.10]
ZHOU 2014	3	50	2.1%	0.06 [0.01; 0.17]
JIANG 2020	21	267	10.2%	0.08 [0.05; 0.12]
KONG 2020	1	50	0.8%	0.02 [0.00; 0.11]
KONG 2016	9	118	5.5%	0.08 [0.04; 0.14]
Total (95% CI)		2221	47.5%	0.07 [0.05; 0.10]
INDIA				
SAHOO 2020	7	95	4.4%	0.07 [0.03; 0.15]
Total (95% CI)		95	4.4%	0.07 [0.04; 0.15]
IRAN				
REZAEIZADEH 2018	8	85	4.9%	0.09 [0.04; 0.18]
Total (95% CI)		85	4.9%	0.09 [0.05; 0.18]
MALAYSIA				
BOO 2012	98	1246	20.7%	0.08 [0.06; 0.10]
Total (95% CI)		1246	20.7%	0.08 [0.06; 0.09]
THAILAND				
PRIYAPOKIN 2020	7	50	4.2%	0.14 [0.06; 0.27]
Total (95% CI)		50	4.2%	0.14 [0.07; 0.27]
TUNISIA				
CHIOUKH 2018	6	109	4.0%	0.06 [0.02; 0.12]
Total (95% CI)		109	4.0%	0.06 [0.02; 0.12]
TURKEY				
SERCE 2014	13	156	7.2%	0.08 [0.05; 0.14]
KARABULUT 2019	7	87	4.4%	0.08 [0.03; 0.16]
KULALI 2019	4	45	2.7%	0.09 [0.02; 0.21]
Total (95% CI)		288	14.3%	0.08 [0.06; 0.12]
**Total (95% CI)**		**4094**	**100.0%**	**0.08 [0.07; 0.10]**

Heterogeneity: Tau2 = 0.0279; Chi2 = 20.39, df = 14 (P = 0.12); I2 = 31%

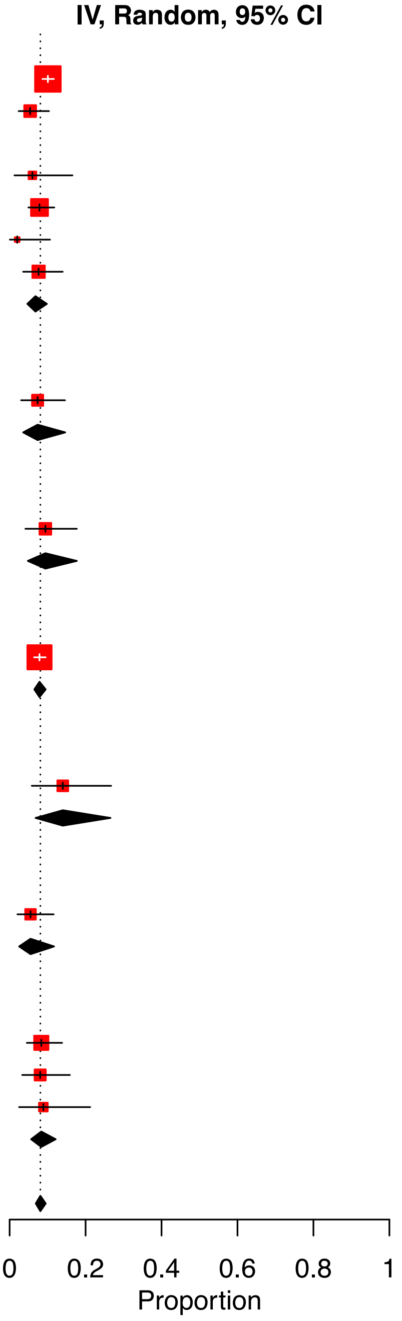

Supplement Figure 35: Publication bias for the secondary outcome - NEC in ELGANs.

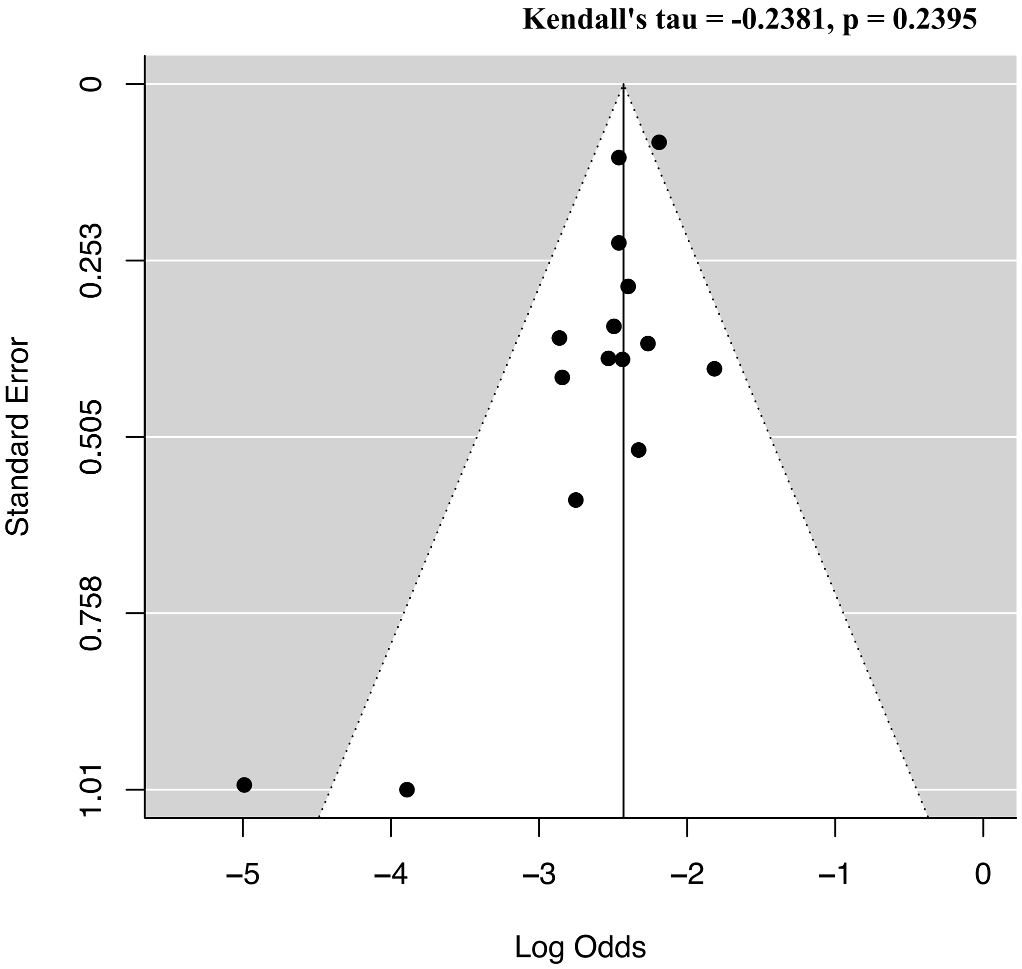

Supplement Figure 36: Secondary outcome- EUGR in ELBWs

Study or Subgroup	Events	Total	Weight	IV, Random, 95% CI
SOUTH AFRICA				
MABHANDI 2019	78	89	84.4%	0.88 [0.79; 0.94]
Total (95% CI)		89	84.4%	0.88 [0.79; 0.93]
UGANDA				
NAKUBULWA2020	16	18	15.6%	0.89 [0.65; 0.99]
Total (95% CI)		18	15.6%	0.89 [0.65; 0.97]
**Total (95% CI)**		**107**	**100.0%**	**0.88 [0.80; 0.93]**

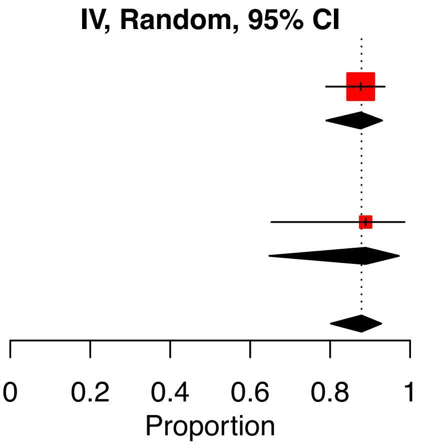

Heterogeneity: Tau = 0; Chi = 0.02, df = 1 (P = 0.88); I = 0%

Supplement Figure 37: Secondary outcome- Any ROP in ELBW neonates

Study or Subgroup	Events	Total	Weight	IV, Random, 95% CI
ARGENTINA				
LOMUTO 2008	85	190	2.6%	0.45 [0.38; 0.52]
Total (95% CI)		190	2.6%	0.45 [0.38; 0.52]
BOTSWANA				
GEZMU 2020A	12	32	2.2%	0.38 [0.21; 0.56]
Total (95% CI)		32	2.2%	0.38 [0.21; 0.54]
BRAZIL				
GONCLAVES 2014	14	24	2.1%	0.58 [0.37; 0.78]
LERMANN 2008	14	28	2.2%	0.50 [0.31; 0.69]
GOULART 2011	22	36	2.3%	0.61 [0.43; 0.77]
MEDINA VALENTON 2016	18	25	2.2%	0.72 [0.51; 0.88]
Total (95% CI)		113	8.7%	0.61 [0.52; 0.70]
CHINA				
LIU 2014	15	24	2.1%	0.62 [0.41; 0.81]
LIN 2015	58	140	2.5%	0.41 [0.33; 0.50]
LIU 2005	22	37	2.3%	0.59 [0.42; 0.75]
WU 2018	21	60	2.4%	0.35 [0.23; 0.48]
YAU 2014	70	131	2.5%	0.53 [0.45; 0.62]
ZHANG 2020	557	1099	2.6%	0.51 [0.48; 0.54]
CHEN 2015	122	161	2.6%	0.76 [0.68; 0.82]
Total (95% CI)		1652	17.1%	0.54 [0.44; 0.64]
EGYPT				
ABDELHADI 2013	18	26	2.2%	0.69 [0.48; 0.86]
HAKEEM 2012	2	3	0.9%	0.67 [0.09; 0.99]
Total (95% CI)		29	4.0%	0.69 [0.53; 0.85]
GHANA				
BRAIMAH 2020	12	20	2.0%	0.60 [0.36; 0.81]
Total (95% CI)		20	2.0%	0.60 [0.39; 0.81]
INDIA				
SEHGAL 2004	13	26	2.1%	0.50 [0.30; 0.70]
SIVANANDAN 2016	41	125	2.5%	0.33 [0.25; 0.42]
SIVANANDAN 2016	30	110	2.5%	0.27 [0.19; 0.37]
OOMMEN 2019	12	29	2.2%	0.41 [0.24; 0.61]
ROY 2006	12	36	2.3%	0.33 [0.19; 0.51]
MUKHOPADHYAY 2013	38	272	2.6%	0.14 [0.10; 0.19]
AGGARWAL 2002	4	12	1.8%	0.33 [0.10; 0.65]
AHUJA 2018	28	39	2.3%	0.72 [0.55; 0.85]
CHAUDHARI 2009	21	58	2.4%	0.36 [0.24; 0.50]
Total (95% CI)		707	20.8%	0.37 [0.25; 0.49]
INDONESIA				
SISWANTO 2018	32	59	2.4%	0.54 [0.41; 0.67]
Total (95% CI)		59	2.4%	0.54 [0.42; 0.67]
IRAN				
SAEIDI 2008	19	52	2.4%	0.37 [0.24; 0.51]
SAEIDI 2017	7	14	1.8%	0.50 [0.23; 0.77]
SABZEHEI 2013	22	53	2.4%	0.42 [0.28; 0.56]
ALIZADEH 2015	8	17	1.9%	0.47 [0.23; 0.72]
GHASEMINEJAD 2011	5	7	1.5%	0.71 [0.29; 0.96]
Total (95% CI)		143	10.0%	0.43 [0.35; 0.51]
JORDAN				
GHARAIBEH 2011	8	11	1.8%	0.73 [0.39; 0.94]
Total (95% CI)		11	1.8%	0.73 [0.46; 0.99]
MEXICO				
LARA–MOLINA 2013	18	44	2.3%	0.41 [0.26; 0.57]
MARTINEZ–CRUZ 2012	34	139	2.6%	0.24 [0.18; 0.32]
Total (95% CI)		183	4.9%	0.31 [0.15; 0.47]
SOUTH AFRICA				
TSHEHLA 2019	2	49	2.6%	0.04 [0.00; 0.14]
Total (95% CI)		49	2.6%	0.04 [0.00; 0.10]
SUDAN				
OMER 2014	2	6	1.4%	0.33 [0.04; 0.78]
Total (95% CI)		6	1.4%	0.33 [0.00; 0.71]
TURKEY				
SAYGILI 2016	72	110	2.5%	0.65 [0.56; 0.74]
ARAZ–ERSAN 2013	311	410	2.6%	0.76 [0.71; 0.80]
ATALAY 2013	11	20	2.0%	0.55 [0.32; 0.77]
CENK CELEBI 2014	173	235	2.6%	0.74 [0.67; 0.79]
CETINKAYA 2014	50	197	2.6%	0.25 [0.19; 0.32]
GEBE..ÇE 2016	6	18	2.0%	0.33 [0.13; 0.59]
ZIYLAN 2006	75	114	2.5%	0.66 [0.56; 0.74]
BAS 2015	1506	2694	2.6%	0.56 [0.54; 0.58]
Total (95% CI)		3798	19.5%	0.57 [0.46; 0.69]
**Total (95% CI)**		**6992**	**100.0%**	**0.49 [0.42; 0.55]**

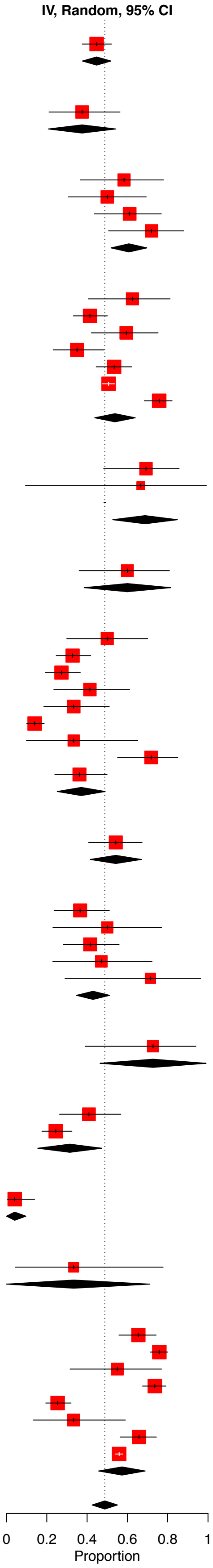

Heterogeneity Tau2= 0.3686; Chi2 = 504.36, df = 43 (P < 0.01); I2 = 91%

Supplement Figure 38: Publication bias for the secondary outcome - Any ROP in ELBW neonates.

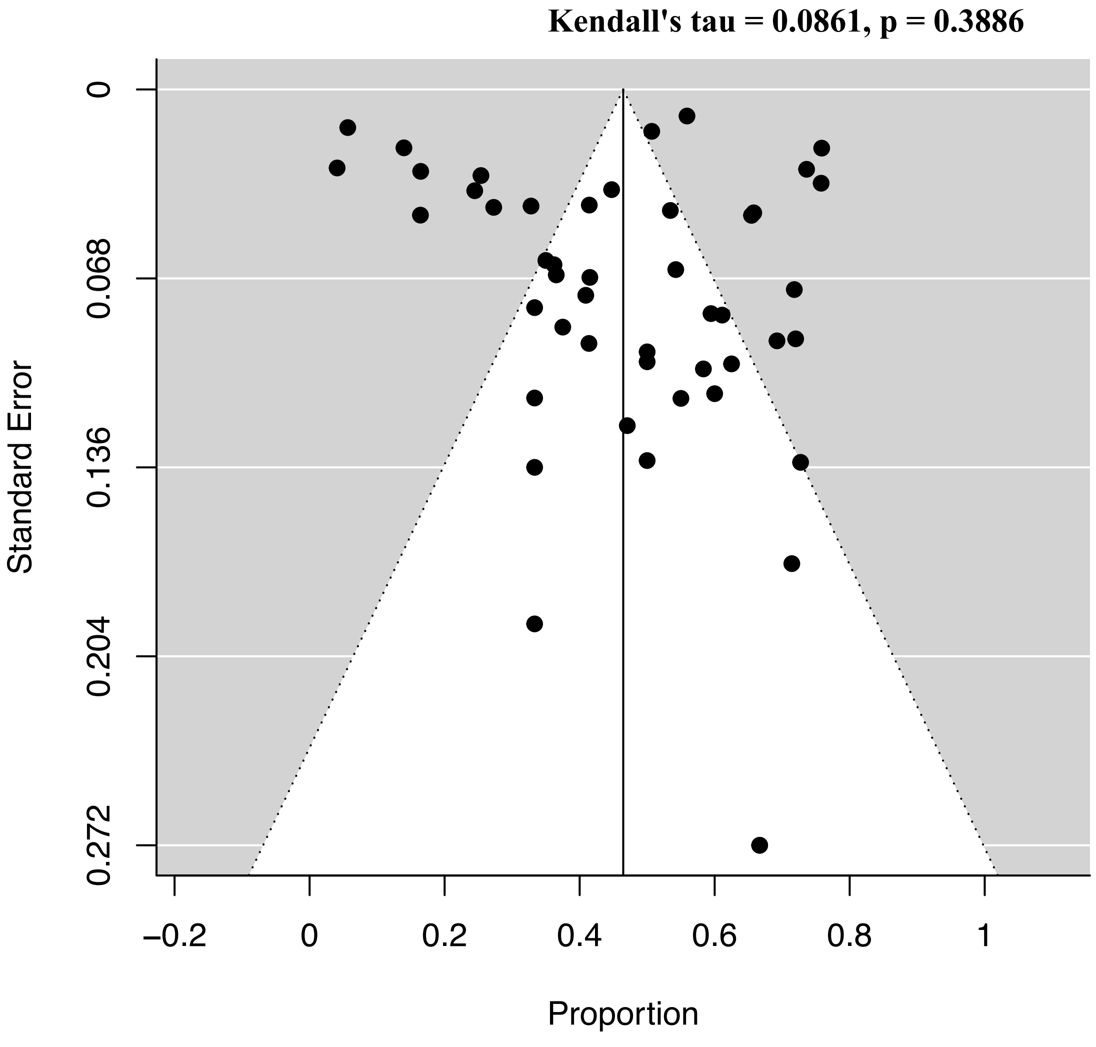

Supplement Figure 39: Secondary outcome- Any ROP in ELGANs

Study or Subgroup	Events	Total	Weight	IV, Random, 95% CI
BOTSWANA				
GEZMU 2020A	6	37	3.3%	0.16 [0.06; 0.32]
Total (95% CI)		37	3.3%	0.16 [0.07; 0.32]
BRAZIL				
LERMANN 2008	5	7	1.7%	0.71 [0.29; 0.96]
MEDINA VALENTON 2016	20	23	2.4%	0.87 [0.66; 0.97]
Total (95% CI)		30	4.1%	0.82 [0.64; 0.93]
CHINA				
LIU 2014	35	85	4.6%	0.41 [0.31; 0.52]
WU 2019	504	1117	5.2%	0.45 [0.42; 0.48]
XU 2019	17	148	4.4%	0.11 [0.07; 0.18]
ZHOU 2014	31	50	4.2%	0.62 [0.47; 0.75]
KONG 2020	9	50	3.7%	0.18 [0.09; 0.31]
KONG 2016	17	101	4.4%	0.17 [0.10; 0.26]
Total (95% CI)		1551	26.6%	0.30 [0.17; 0.46]
EGYPT				
ABDELHADI 2013	13	15	1.9%	0.87 [0.60; 0.98]
Total (95% CI)		15	3.8%	0.87 [0.69; 0.95]
GHANA				
BRAIMAH 2020	18	28	3.6%	0.64 [0.44; 0.81]
Total (95% CI)		28	3.6%	0.64 [0.45; 0.80]
INDIA				
SEHGAL 2004	7	9	1.8%	0.78 [0.40; 0.97]
SIVANANDAN 2016	24	39	4.0%	0.62 [0.45; 0.77]
Total (95% CI)		48	5.8%	0.64 [0.50; 0.76]
INDONESIA				
SISWANTO 2018	19	47	4.2%	0.40 [0.26; 0.56]
Total (95% CI)		47	4.2%	0.40 [0.27; 0.55]
IRAN				
SABZEHEI 2013	30	84	4.6%	0.36 [0.26; 0.47]
ALIZADEH 2015	17	32	3.8%	0.53 [0.35; 0.71]
KARKHANEH 2008	139	199	4.9%	0.70 [0.63; 0.76]
Total (95% CI)		315	13.4%	0.53 [0.30; 0.75]
MEXICO				
CAUICH–ARAGON 2017	2	2	0.6%	1.00 [0.16; 1.00]
ZEPEDA ROMERO 2016	5	9	2.2%	0.56 [0.21; 0.86]
Total (95% CI)		11	2.9%	0.61 [0.32; 0.84]
NEPAL				
SHRESTHA 2010	10	12	1.9%	0.83 [0.52; 0.98]
Total (95% CI)		12	1.9%	0.83 [0.52; 0.96]
PAKISTAN				
TAQUI 2008	10	15	2.8%	0.67 [0.38; 0.88]
Total (95% CI)		15	2.8%	0.67 [0.41; 0.85]
SOUTH AFRICA				
KIFTA 2016	44	98	4.7%	0.45 [0.35; 0.55]
Total (95% CI)		98	4.7%	0.45 [0.35; 0.55]
TURKEY				
SAHIN 2014	72	109	4.7%	0.66 [0.56; 0.75]
SAYGILI 2016	111	203	5.0%	0.55 [0.48; 0.62]
OZCAN 2015	11	18	3.1%	0.61 [0.36; 0.83]
ARAZ–ERSAN 2013	228	283	5.0%	0.81 [0.75; 0.85]
BAS 2015	1975	3737	5.3%	0.53 [0.51; 0.54]
Total (95% CI)		4350	23.1%	0.64 [0.51; 0.75]
**Total (95% CI)**		**6557**	**100.0%**	**0.53 [0.46; 0.59]**

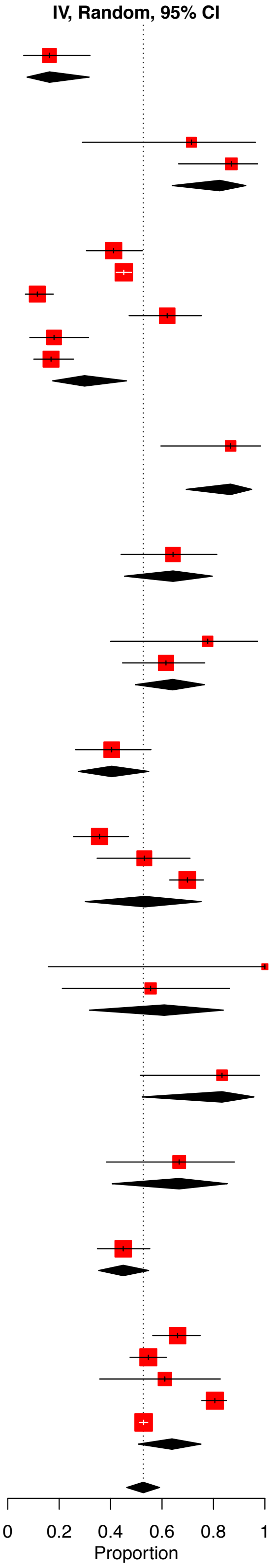

Heterogeneity Tau2= 0.3256; Chi2 = 327.97, df = 27 (P < 0.01); I2 = 92%

Supplement Figure 40: Publication bias for the secondary outcome - Any ROP in ELGANs.

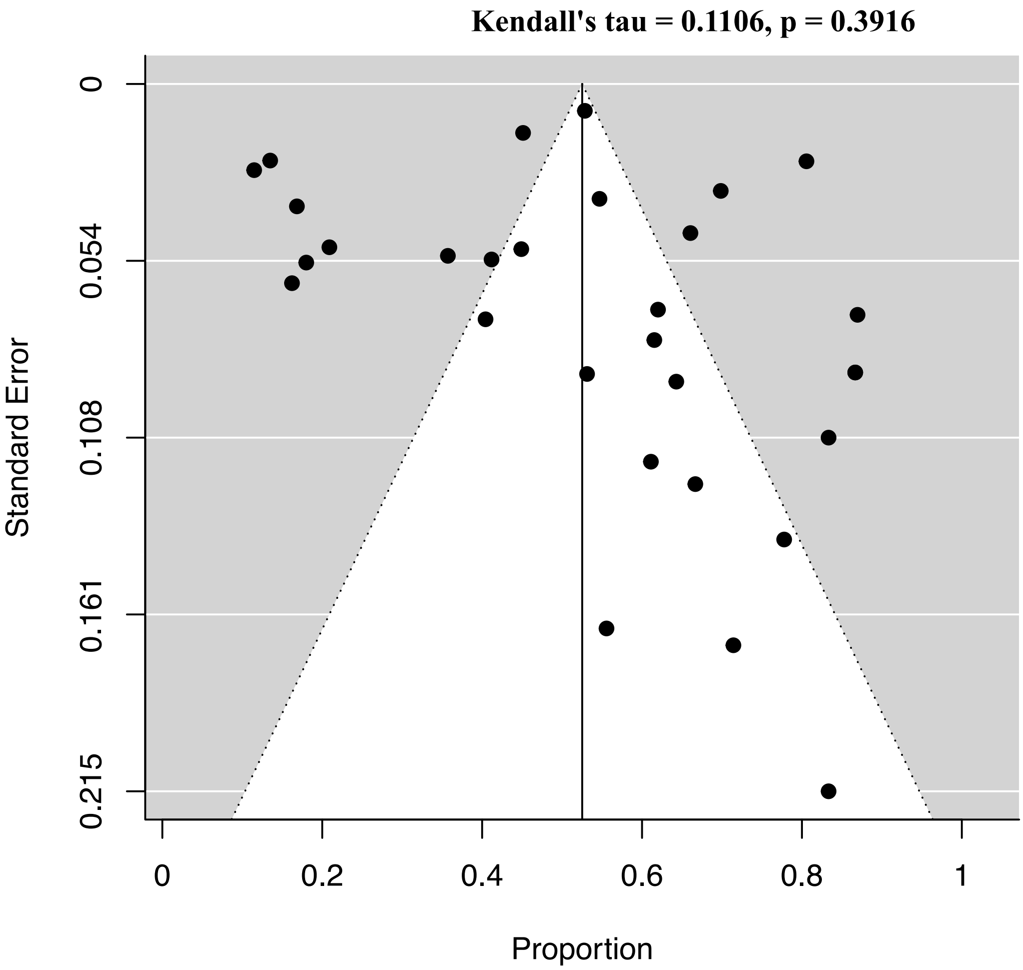

Supplement Figure 41: Secondary outcome- Severe ROP in ELBW neonates

Study or Subgroup	Events	Total	Weight	IV, Random, 95% CI
ARGENTINA				
LOMUTO 2008	50	190	6.9%	0.26 [0.20; 0.33]
Total (95% CI)		190	6.9%	0.26 [0.21; 0.33]
CHINA				
LIU 2005	4	37	4.1%	0.11 [0.03; 0.25]
WANG 2012	9	160	5.5%	0.06 [0.03; 0.10]
WU 2018	12	60	5.7%	0.20 [0.11; 0.32]
YAU 2014	19	131	6.3%	0.15 [0.09; 0.22]
ZHANG 2020	328	1099	7.4%	0.30 [0.27; 0.33]
Total (95% CI)		1487	34.1%	0.14 [0.08; 0.25]
INDIA				
THAKRE 2017	4	7	2.7%	0.57 [0.18; 0.90]
Total (95% CI)		7	2.7%	0.57 [0.23; 0.86]
IRAN				
SABZEHEI 2013	20	53	6.0%	0.38 [0.25; 0.52]
ALIZADEH 2015	6	17	4.2%	0.35 [0.14; 0.62]
KARKHANEH 2008	71	117	6.8%	0.61 [0.51; 0.70]
Total (95% CI)		187	17.0%	0.46 [0.29; 0.64]
PAKISTAN				
TAQUI 2008	8	14	4.0%	0.57 [0.29; 0.82]
Total (95% CI)		14	4.0%	0.57 [0.32; 0.79]
THAILAND				
SRITIPSUKHO 2007	0	21	1.1%	0.00 [0.00; 0.16]
Total (95% CI)		21	1.1%	0.02 [0.00; 0.28]
TURKEY				
SAYGILI 2016	29	110	6.6%	0.26 [0.18; 0.36]
SERCE 2014	20	179	6.4%	0.11 [0.07; 0.17]
ARAZ–ERSAN 2013	149	410	7.3%	0.36 [0.32; 0.41]
ZIYLAN 2006	22	114	6.4%	0.19 [0.13; 0.28]
BAS 2015	503	2694	7.4%	0.19 [0.17; 0.20]
Total (95% CI)		3507	34.1%	0.21 [0.14; 0.31]
**Total (95% CI)**		**5413**	**100.0%**	**0.24 [0.19; 0.30]**

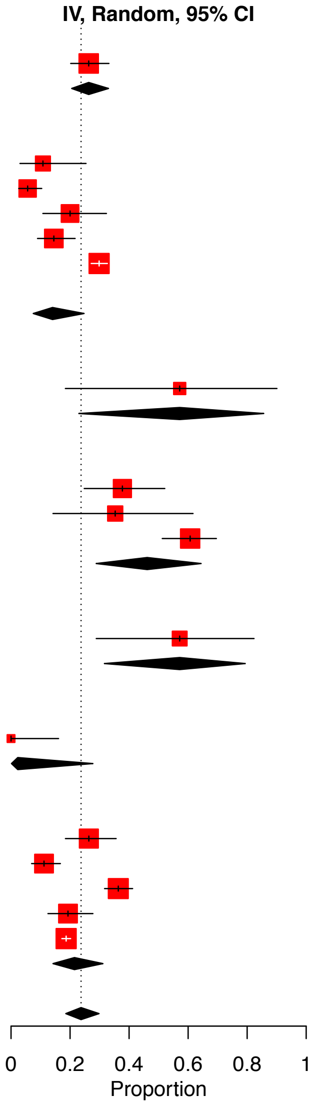

Heterogeneity: Tau2= 0.3385; Chi2 = 243.42, df = 17 (P < 0.01); I2 = 93%

Supplement Figure 42: Publication bias for the secondary outcome - Severe ROP in ELBW neonates.

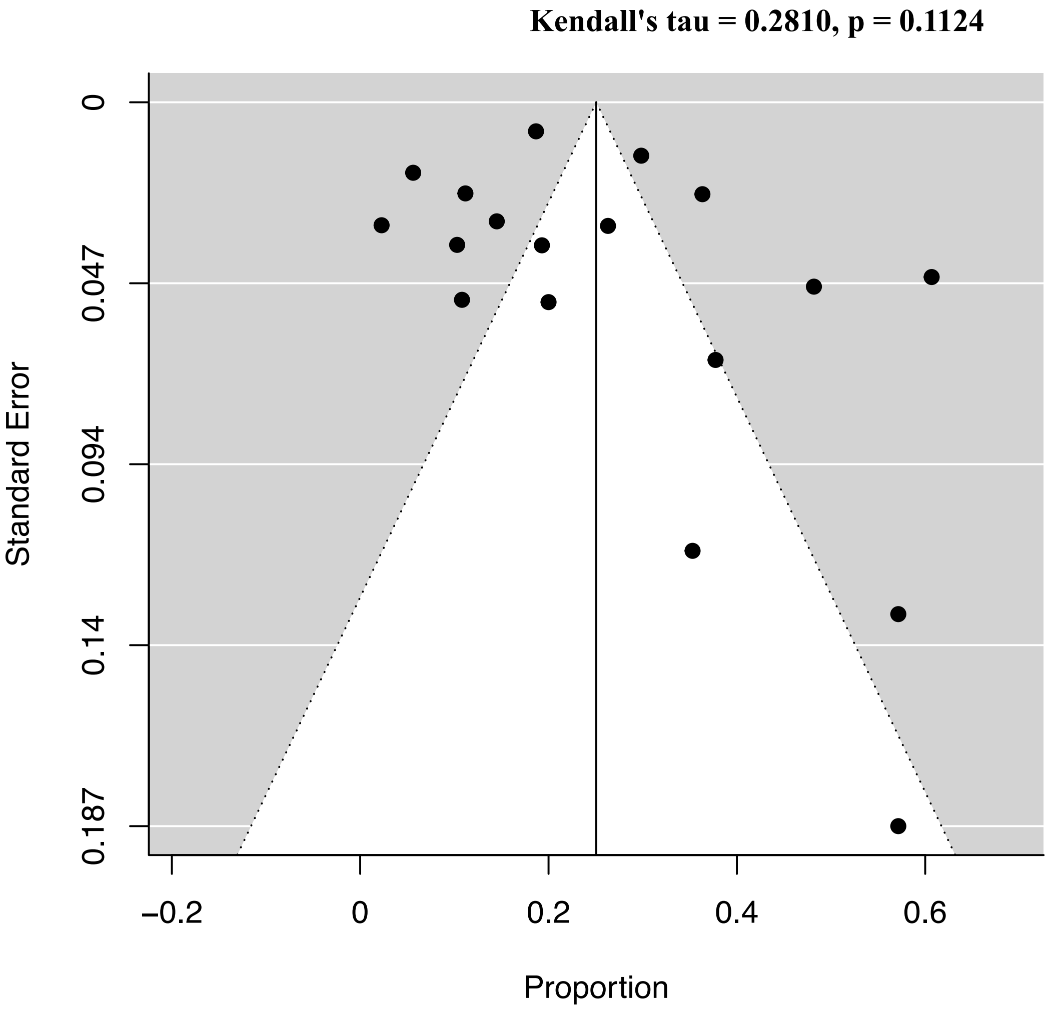

Supplement Figure 43: Secondary outcome- Severe ROP in ELGANs

Study or Subgroup	Events	Total	Weight	IV, Random, 95% CI
BRAZIL				
BONOTTO 2007	5	12	5.1%	0.42 [0.15; 0.72]
Total (95% CI)		12	5.1%	0.42 [0.18; 0.69]
CHINA				
WU 2019	163	1117	8.2%	0.15 [0.13; 0.17]
XU 2019	3	148	5.1%	0.02 [0.00; 0.06]
KONG 2020	2	50	4.2%	0.04 [0.00; 0.14]
Total (95% CI)		1315	17.5%	0.05 [0.01; 0.21]
EGYPT				
HADI 2013	3	15	4.7%	0.20 [0.04; 0.48]
Total (95% CI)		15	4.7%	0.20 [0.07; 0.47]
IRAN				
SABZEHEI 2013	27	84	7.5%	0.32 [0.22; 0.43]
ALIZADEH 2015	12	32	6.7%	0.38 [0.21; 0.56]
Total (95% CI)		116	14.2%	0.34 [0.26; 0.43]
NEPAL				
SHRESTHA 2010	4	12	4.9%	0.33 [0.10; 0.65]
Total (95% CI)		12	4.9%	0.33 [0.13; 0.62]
[illegible]				
TURKEY				
SAHIN 2014	42	109	7.7%	0.39 [0.29; 0.48]
SAYGILI 2016	80	203	8.0%	0.39 [0.33; 0.46]
SERCE 2014	17	156	7.4%	0.11 [0.06; 0.17]
ARAZ–ERSAN 2013	135	283	8.1%	0.48 [0.42; 0.54]
BAS 2015	565	3737	8.2%	0.15 [0.14; 0.16]
KULALI 2018	11	45	6.8%	0.24 [0.13; 0.40]
Total (95% CI)		4533	46.2%	0.27 [0.15; 0.45]
**Total (95% CI)**		**6003**	**100.0%**	**0.22 [0.16; 0.30]**

Heterogeneity: Tau2 = 0.5497; Chi2 = 307.09, df = 14 (P < 0.01); I2 = 95%

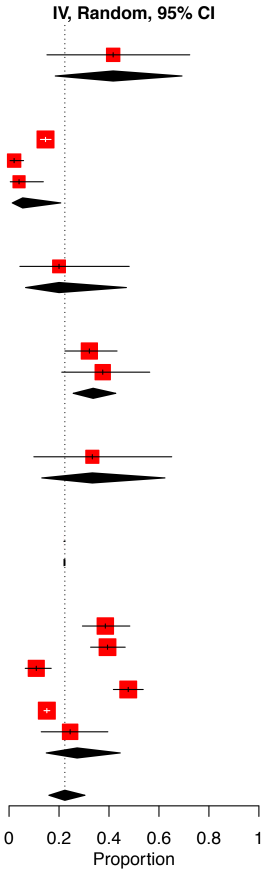

Supplement Figure 44: Publication bias for the secondary outcome - Severe ROP in ELGANs.

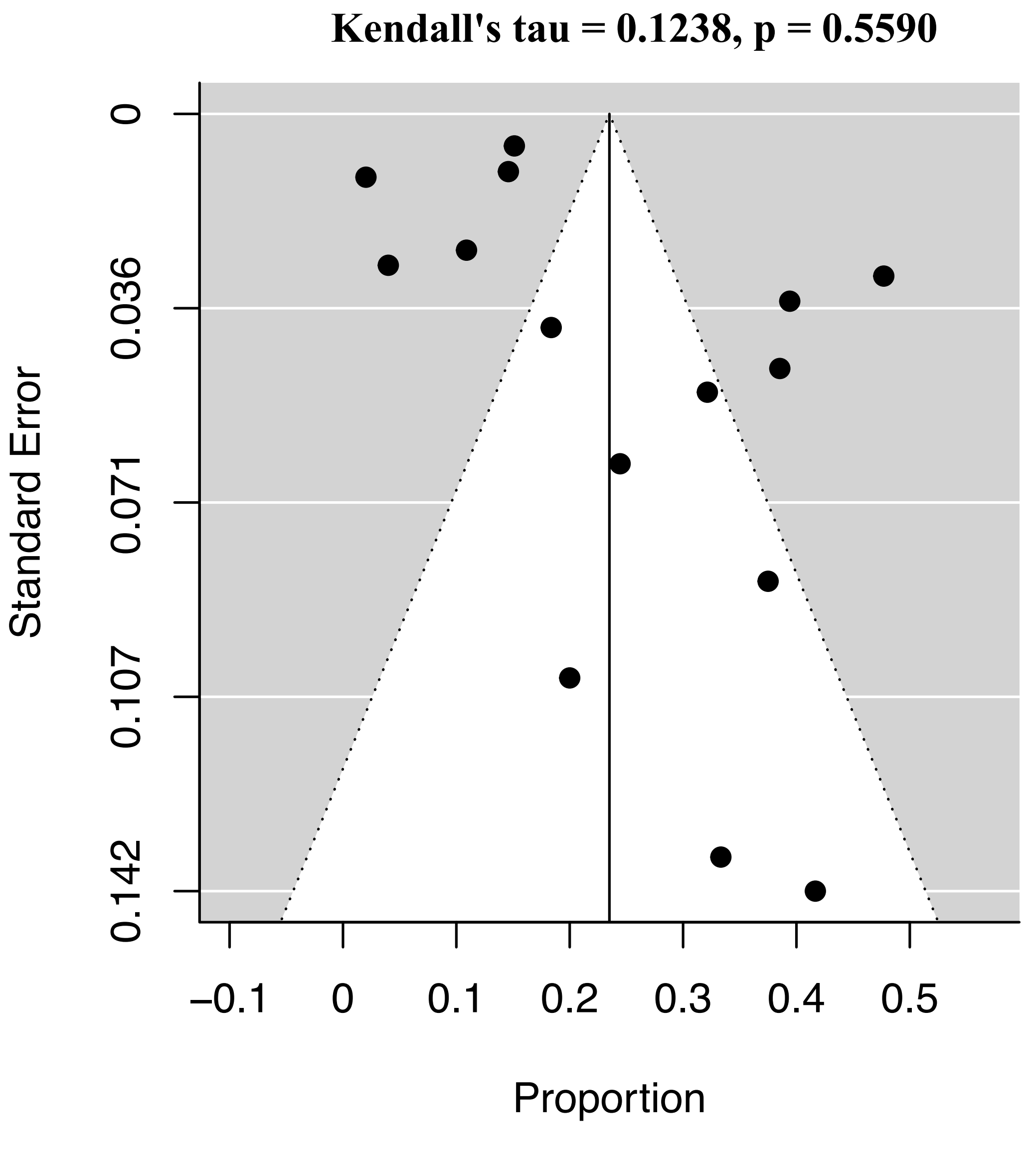

Supplement Figure 45: Secondary outcome- ROP requiring intervention in ELBW neonates

Study or Subgroup	Events	Total	Weight	IV, Random, 95% CI
ARGENTINA				
LOMUTO 2008	26	190	10.2%	0.14 [0.09; 0.19]
Total (95% CI)		190	10.2%	0.14 [0.09; 0.19]
BRAZIL				
LERMANN 2008	3	28	6.9%	0.11 [0.02; 0.28]
Total (95% CI)		28	6.9%	0.11 [0.03; 0.28]
CHINA				
LIN 2015	20	140	10.0%	0.14 [0.09; 0.21]
CHEN 2015	61	161	10.5%	0.38 [0.30; 0.46]
Total (95% CI)		301	20.5%	0.24 [0.08; 0.54]
INDIA				
SIVANANDAN 2016	18	125	9.9%	0.14 [0.09; 0.22]
MUKHOPADHYAY 2013	12	272	9.6%	0.04 [0.02; 0.08]
CHAUDHARI 2009	11	58	9.3%	0.19 [0.10; 0.31]
Total (95% CI)		455	28.9%	0.11 [0.05; 0.24]
IRAN				
SABZEHEI 2013	11	53	9.3%	0.21 [0.11; 0.34]
Total (95% CI)		53	9.3%	0.21 [0.12; 0.34]
JORDAN				
GHARAIBEH 2011	4	11	6.8%	0.36 [0.11; 0.69]
Total (95% CI)		11	6.8%	0.36 [0.14; 0.66]
TURKEY				
ATALAY 2013	3	20	6.8%	0.15 [0.03; 0.38]
CENK CELEBI 2014	86	235	10.6%	0.37 [0.30; 0.43]
Total (95% CI)		255	17.4%	0.27 [0.11; 0.53]
**Total (95% CI)**		**1293**	**100.0%**	**0.18 [0.12; 0.27]**

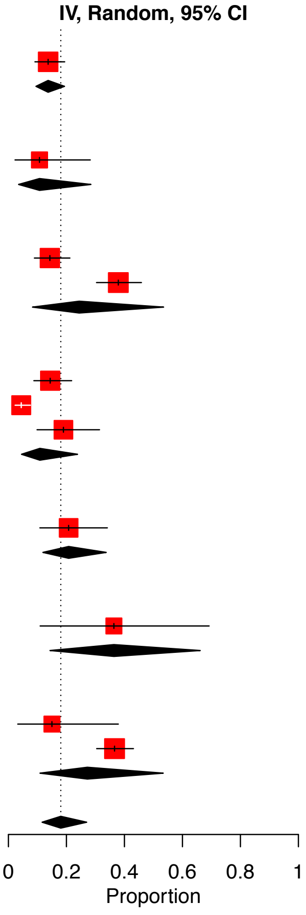

Heterogeneity: Tau2 = 0.6372; Chi2 = 107.53, df = 10 (P < 0.01); I2 = 91%

Supplement Figure 46: Publication bias for the secondary outcome - ROP requiring intervention in ELBW neonates.

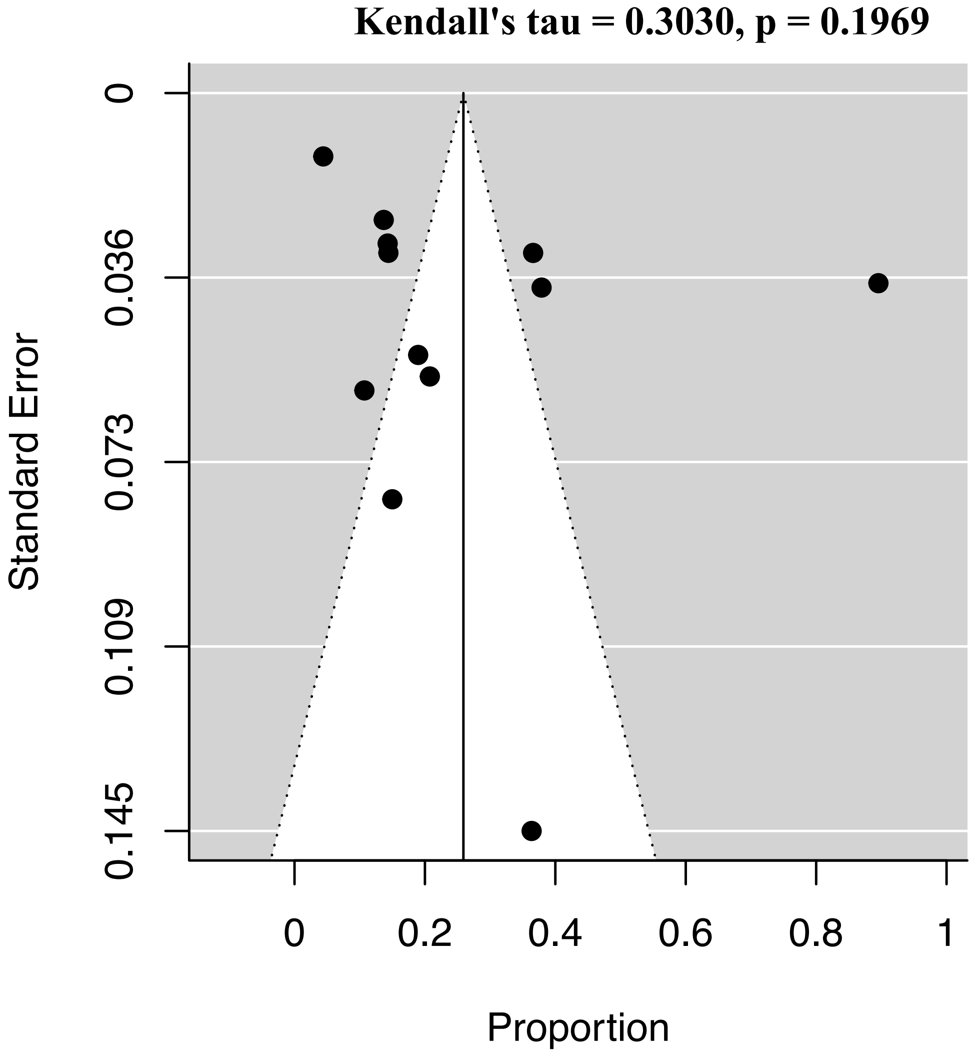

Supplement Figure 47: Secondary outcome- ROP requiring intervention in ELGANs

Study or Subgroup	Events	Total	Weight	IV, Random, 95% CI
BRAZIL				
LERMANN 2008	3	7	4.6%	0.43 [0.10; 0.82]
Total (95% CI)		7	4.6%	0.43 [0.14; 0.77]
CHINA				
WU 2019	88	1117	8.3%	0.08 [0.06; 0.10]
ZHOU 2014	6	50	6.6%	0.12 [0.05; 0.24]
Total (95% CI)		1167	14.9%	0.08 [0.06; 0.10]
GHANA				
BRAIMAH 2020	3	28	5.5%	0.11 [0.02; 0.28]
Total (95% CI)		28	10.2%	0.10 [0.04; 0.23]
INDIA				
SIVANANDAN 2016	11	39	7.2%	0.28 [0.15; 0.45]
SAHOO 2020	14	95	7.5%	0.15 [0.08; 0.23]
CHAUDHARI 2009	12	58	7.3%	0.21 [0.11; 0.33]
Total (95% CI)		192	22.0%	0.20 [0.14; 0.29]
IRAN				
SABZEHEI 2013	20	84	7.7%	0.24 [0.15; 0.34]
Total (95% CI)		84	7.7%	0.24 [0.16; 0.34]
MEXICO				
ZEPEDA ROMERO 2016	3	9	4.9%	0.33 [0.07; 0.70]
Total (95% CI)		9	4.9%	0.33 [0.11; 0.67]
THAILAND				
PIRIYAPOKIN 2020	21	50	7.6%	0.42 [0.28; 0.57]
Total (95% CI)		50	7.6%	0.42 [0.29; 0.56]
TURKEY				
SAHIN 2014	39	109	8.0%	0.36 [0.27; 0.46]
OZCAN 2015	4	18	5.8%	0.22 [0.06; 0.48]
KARABULUT 2019	9	87	7.2%	0.10 [0.05; 0.19]
KULALI 2018	9	45	7.0%	0.20 [0.10; 0.35]
Total (95% CI)		259	28.0%	0.21 [0.11; 0.37]
**Total (95% CI)**		**1796**	**100.0%**	**0.20 [0.13; 0.29]**

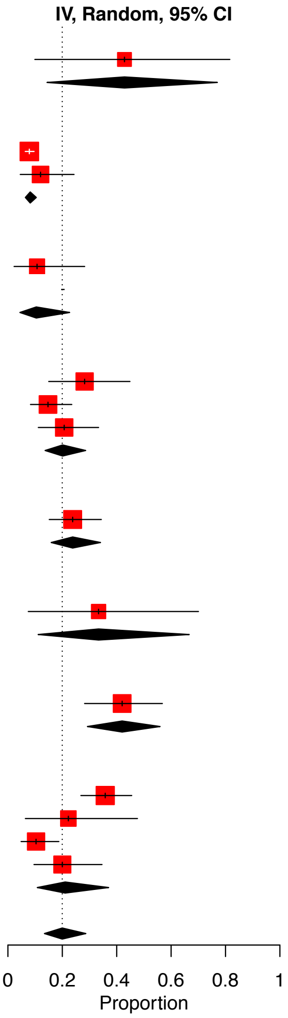

Heterogeneity Tau2= 0.6978; Chi = 121.06, df = 14 (P < 0.01); I2 = 88%

Supplement Figure 48: Publication bias for the secondary outcome - ROP requiring intervention in ELGANs.

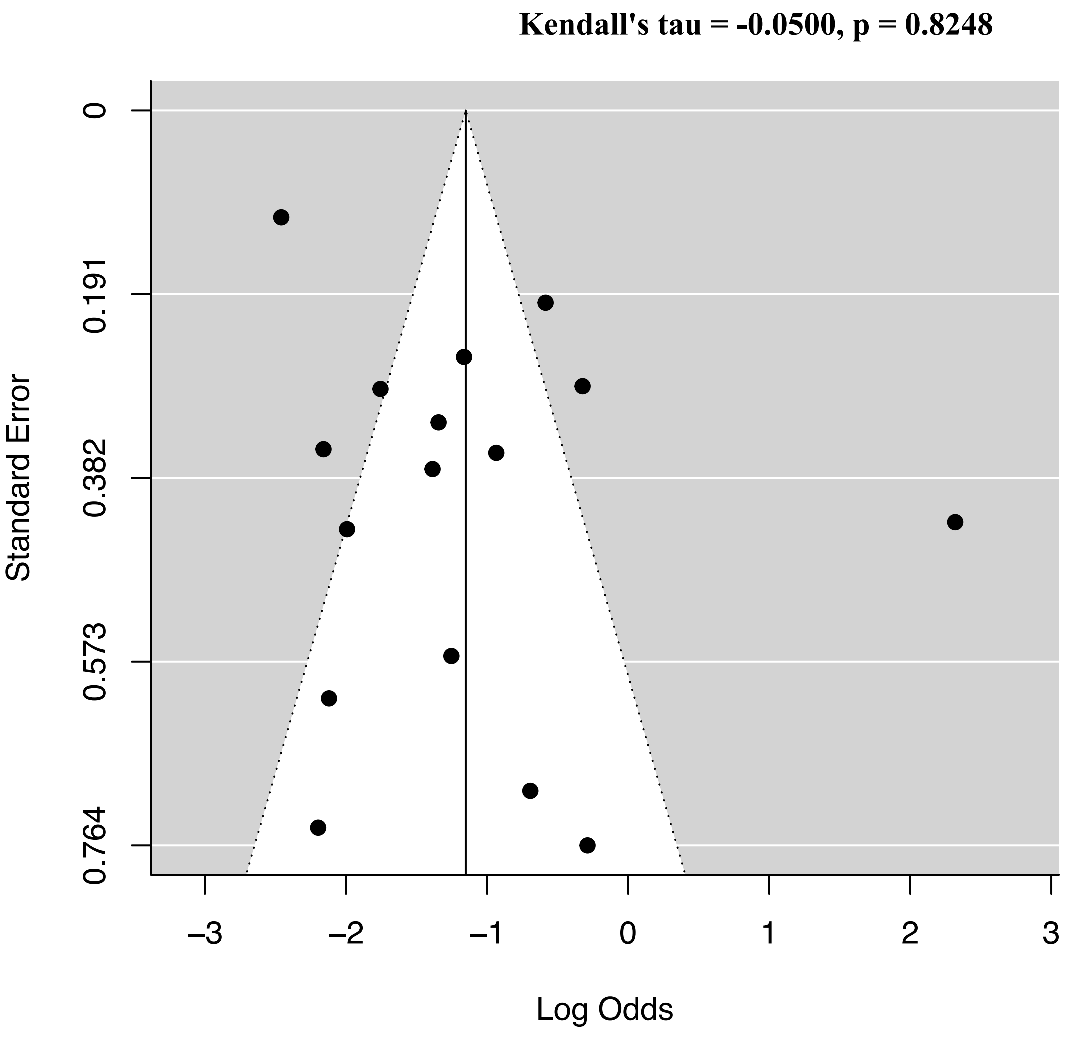

Supplement Figure 49: Sensitivity analysis - primary outcome - survival until discharge for ELBW neonates analyzed based on country of origin after excluding small sample size studies.

Study or Subgroup	Events	Total	Weight	IV, Random, 95% CI
ARGENTINA				
BUENOS AIRES 2012	182	451	2.1%	0.40 [0.36; 0.45]
Total (95% CI)		451	2.1%	0.40 [0.36; 0.45]
BANGLADESH				
ALI 2016	43	113	1.9%	0.38 [0.29; 0.48]
Total (95% CI)		113	1.9%	0.38 [0.30; 0.47]
BENIN				
OMOIGBERALE 2009	99	151	2.0%	0.66 [0.57; 0.73]
Total (95% CI)		151	2.0%	0.66 [0.58; 0.73]
BRAZIL				
PINHEIRO 2010	146	445	2.1%	0.33 [0.28; 0.37]
AZEREDO CARDOSO 2013	151	305	2.1%	0.50 [0.44; 0.55]
CARNEIRO 2012	27	57	1.7%	0.47 [0.34; 0.61]
DE BRITO 2003	38	79	1.8%	0.48 [0.37; 0.60]
DE MELLO 2007	28	60	1.8%	0.47 [0.34; 0.60]
DE ARAÚJO 2007	19	61	1.7%	0.31 [0.20; 0.44]
VILANOVA 2019	596	1325	2.1%	0.45 [0.42; 0.48]
CASTRO 2007	55	270	2.0%	0.20 [0.16; 0.26]
Total (95% CI)		2602	15.3%	0.39 [0.32; 0.47]
CAMEROON				
CHIABI 2014	3	74	1.0%	0.04 [0.01; 0.11]
Total (95% CI)		74	1.0%	0.04 [0.01; 0.12]
CHINA				
LIN 2015	129	258	2.1%	0.50 [0.44; 0.56]
WU 2019	598	1303	2.1%	0.46 [0.43; 0.49]
ZHANG 2020	68	100	1.9%	0.68 [0.58; 0.77]
Total (95% CI)		1661	6.1%	0.54 [0.43; 0.63]
COLOMBIA				
RUIZ–PELAEZ 2014	40	86	1.9%	0.47 [0.36; 0.58]
Total (95% CI)		86	1.9%	0.47 [0.36; 0.57]
ETHIOPIA				
MUHE 2019	28	165	1.9%	0.17 [0.12; 0.24]
MEKASHA 2020	28	164	1.9%	0.17 [0.12; 0.24]
Total (95% CI)		329	3.8%	0.17 [0.13; 0.21]
GHANA				
SACKEY 2019	113	560	2.1%	0.20 [0.17; 0.24]
WELBECK 2003	21	128	1.8%	0.16 [0.10; 0.24]
Total (95% CI)		688	3.9%	0.20 [0.17; 0.23]
INDIA				
SEHGAL 2004	30	52	1.7%	0.58 [0.43; 0.71]
BAJAJ 2020	29	97	1.8%	0.30 [0.21; 0.40]
MUKHOPADHYAY 2013	134	436	2.1%	0.31 [0.26; 0.35]
THAKUR 2013	173	283	2.1%	0.61 [0.55; 0.67]
NNPD 2004	21	101	1.8%	0.21 [0.13; 0.30]
SAHOO 2020	143	231	2.0%	0.62 [0.55; 0.68]
Total (95% CI)		1200	11.5%	0.43 [0.29; 0.59]
INDONESIA				
SISWANTO 2018	94	182	2.0%	0.52 [0.44; 0.59]
Total (95% CI)		182	2.0%	0.52 [0.44; 0.59]
IRAN				
REZAEIZADEH 2018	69	127	1.9%	0.54 [0.45; 0.63]
NAVAEI 2010	25	107	1.8%	0.23 [0.16; 0.33]
AFJEH 2013A	49	147	2.0%	0.33 [0.26; 0.42]
BASIRI 2015	11	64	1.6%	0.17 [0.09; 0.29]
HAGHIGHI 2013	11	52	1.5%	0.21 [0.11; 0.35]
Total (95% CI)		497	8.8%	0.29 [0.18; 0.44]
JAMAICA				
TROTMAN 2012	96	286	2.1%	0.34 [0.28; 0.39]
Total (95% CI)		286	2.1%	0.34 [0.28; 0.39]
KOSOVO				
AHMETI 2010	0	73	0.3%	0.00 [0.00; 0.05]
Total (95% CI)		73	0.3%	0.01 [0.00; 0.10]
MALAWI				
RYLANCE 2013	8	71	1.4%	0.11 [0.05; 0.21]
Total (95% CI)		71	1.4%	0.11 [0.06; 0.21]
MEXICO				
MARTINEZ 2010	54	152	2.0%	0.36 [0.28; 0.44]
OSORNO–COVARRUBIAS 2002	78	162	2.0%	0.48 [0.40; 0.56]
SAUCEDO 2008	334	727	2.1%	0.46 [0.42; 0.50]
MONTANO PEREZ 2019	20	52	1.7%	0.38 [0.25; 0.53]
Total (95% CI)		1093	7.8%	0.43 [0.37; 0.49]
MULTI–COUNTRY				
NEOCOSUR 2002	60	126	1.9%	0.48 [0.39; 0.57]
Total (95% CI)		126	1.9%	0.48 [0.39; 0.56]
NIGERIA				
UGWU 2010	6	149	1.3%	0.04 [0.01; 0.09]
Total (95% CI)		149	1.3%	0.04 [0.02; 0.09]
PAKISTAN				
ALI 2019	0	200	0.3%	0.00 [0.00; 0.02]
Total (95% CI)		200	0.3%	0.00 [0.00; 0.04]
SERBIA				
GORDANA 2019	89	157	2.0%	0.57 [0.49; 0.65]
Total (95% CI)		157	2.0%	0.57 [0.49; 0.64]
SOUTH AFRICA				
LUTHULI 2019	52	105	1.9%	0.50 [0.40; 0.59]
BALLOT 2010	50	143	2.0%	0.35 [0.27; 0.43]
BALLOT 2012	28	95	1.8%	0.29 [0.21; 0.40]
BALLOT 2017	224	546	2.1%	0.41 [0.37; 0.45]
HENDRIKS 2014	9	97	1.5%	0.09 [0.04; 0.17]
KALIMBA 2013	101	382	2.1%	0.26 [0.22; 0.31]
TSHEHLA 2019	36	71	1.8%	0.51 [0.39; 0.63]
NTULI 2020	26	50	1.7%	0.52 [0.37; 0.66]
VELAPHI 2005	186	453	2.1%	0.41 [0.36; 0.46]
Total (95% CI)		1942	17.0%	0.36 [0.30; 0.44]
TURKEY				
SERCE 2014	56	179	2.0%	0.31 [0.25; 0.39]
CETINKAYA 2014	102	197	2.0%	0.52 [0.45; 0.59]
Total (95% CI)		376	4.0%	0.41 [0.23; 0.62]
VIETNAM				
MILES 2017	31	63	1.8%	0.49 [0.36; 0.62]
Total (95% CI)		63	1.8%	0.49 [0.37; 0.61]
**Total (95% CI)**		**12570**	**100.0%**	**0.36 [0.33; 0.40]**

Heterogeneity: Tau2 = 0.3000; Chi2 = 931.46, df = 92 (P < 0.01); I2 = 80%

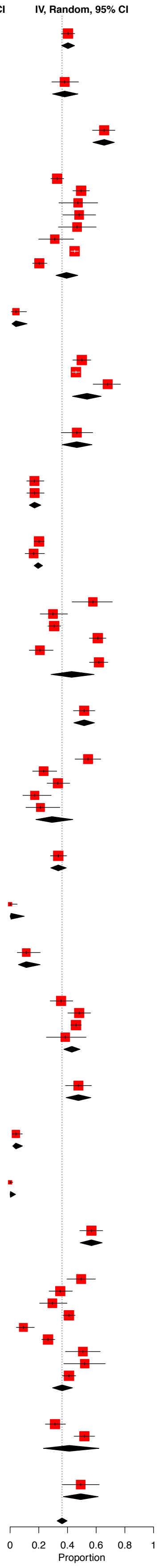

Supplement Figure 50: Sensitivity analysis - primary outcome - survival until discharge for ELGANs analyzed based on country of origin after excluding small sample size studies.

Study or Subgroup	Events	Total	Weight	IV, Random, 95% CI
ARGENTINA				
BUENOS AIRES 2012	225	426	3.0%	0.53 [0.48; 0.58]
Total (95% CI)		426	3.0%	0.53 [0.48; 0.58]
BENIN				
OMOIGBERALE 2009	128	190	2.9%	0.67 [0.60; 0.74]
Total (95% CI)		190	2.9%	0.67 [0.60; 0.74]
BRAZIL				
DE BRITO 2003	30	70	2.7%	0.43 [0.31; 0.55]
DE MELLO 2007	24	51	2.6%	0.47 [0.33; 0.62]
DECARVALHO 2007	34	108	2.8%	0.31 [0.23; 0.41]
Total (95% CI)		229	8.2%	0.40 [0.30; 0.49]
CAMEROON				
CHIABI 2014	6	75	2.2%	0.08 [0.03; 0.17]
Total (95% CI)		75	2.2%	0.08 [0.04; 0.17]
CHINA				
LI 2019	159	307	3.0%	0.52 [0.46; 0.58]
JIANG 2020	235	320	3.0%	0.73 [0.68; 0.78]
KONG 2020	35	50	2.6%	0.70 [0.55; 0.82]
KONG 2016	101	148	2.9%	0.68 [0.60; 0.76]
WU 2019	1076	2051	3.1%	0.52 [0.50; 0.55]
XU 2019	101	148	2.9%	0.68 [0.60; 0.76]
ZHOU 2014	50	72	2.7%	0.69 [0.57; 0.80]
ZHU 2020	308	441	3.0%	0.70 [0.65; 0.74]
Total (95% CI)		3537	23.1%	0.65 [0.58; 0.72]
COLOMBIA				
RUIZ–PELAEZ 2014	43	81	2.8%	0.53 [0.42; 0.64]
Total (95% CI)		81	2.8%	0.53 [0.42; 0.64]
ETHIOPIA				
MUHE 2019	15	104	2.6%	0.14 [0.08; 0.23]
Total (95% CI)		104	2.6%	0.14 [0.09; 0.23]
GHANA				
SACKEY 2019	141	539	3.0%	0.26 [0.22; 0.30]
WELBECK 2003	176	382	3.0%	0.46 [0.41; 0.51]
Total (95% CI)		921	6.0%	0.35 [0.19; 0.57]
INDIA				
MUKHOPADHYAY 2013	19	169	2.7%	0.11 [0.07; 0.17]
THAKUR 2013	28	81	2.8%	0.35 [0.24; 0.46]
SAHOO 2020	43	95	2.8%	0.45 [0.35; 0.56]
Total (95% CI)		345	8.3%	0.28 [0.11; 0.53]
INDONESIA				
SISWANTO 2018	94	185	2.9%	0.51 [0.43; 0.58]
Total (95% CI)		185	2.9%	0.51 [0.44; 0.58]
IRAN				
REZAEIZADEH 2018	40	85	2.8%	0.47 [0.36; 0.58]
NAVAEI 2010	29	122	2.8%	0.24 [0.17; 0.32]
AFJEH 2013	72	196	2.9%	0.37 [0.30; 0.44]
BASIRI 2015	24	68	2.7%	0.35 [0.24; 0.48]
Total (95% CI)		471	11.3%	0.35 [0.27; 0.45]
MEXICO				
OSORNO–COVARRUBIAS 2002	11	50	2.5%	0.22 [0.12; 0.36]
Total (95% CI)		50	2.5%	0.22 [0.13; 0.36]
MULTI–COUNTRY				
NEOCOSUR 2002	43	95	2.8%	0.45 [0.35; 0.56]
Total (95% CI)		95	2.8%	0.45 [0.36; 0.55]
SERBIA				
GORDANA 2019	141	220	3.0%	0.64 [0.57; 0.70]
Total (95% CI)		220	3.0%	0.64 [0.58; 0.70]
SOUTH AFRICA				
LUTHULI 2019	14	53	2.6%	0.26 [0.15; 0.40]
KIRSTEN 2012	231	309	3.0%	0.75 [0.70; 0.80]
Total (95% CI)		362	5.5%	0.51 [0.12; 0.89]
THAILAND				
MCGREADY 2018	2	132	1.5%	0.02 [0.00; 0.05]
Total (95% CI)		132	1.5%	0.02 [0.00; 0.06]
THAILAND				
PIRIYAPOKIN 2020	50	67	2.6%	0.75 [0.63; 0.84]
Total (95% CI)		67	2.6%	0.75 [0.63; 0.84]
TUNISIA				
CHIOUKH 2018	27	109	2.8%	0.25 [0.17; 0.34]
Total (95% CI)		109	2.8%	0.25 [0.18; 0.34]
TURKEY				
SERCE 2014	54	156	2.9%	0.35 [0.27; 0.43]
KULALI 2019	46	165	2.9%	0.28 [0.21; 0.35]
Total (95% CI)		321	5.8%	0.31 [0.25; 0.38]
**Total (95% CI)**		**7920**	**100.0%**	**0.43 [0.38; 0.49]**

Heterogeneity: Tau2 = 0.4977; Chi2 = 877.88, df = 60 (P < 0.01); I2 = 92%

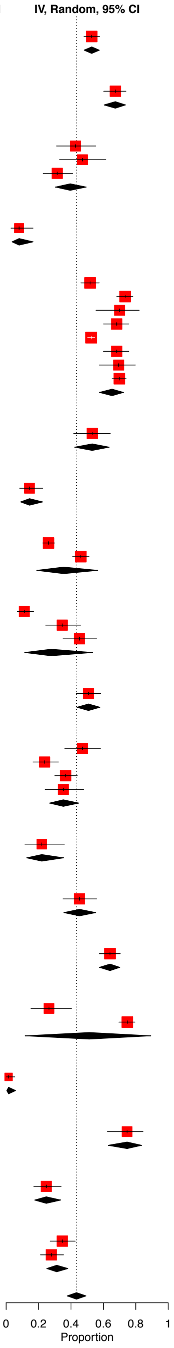

Supplement Figure 51: Sensitivity analysis - primary outcome - Comparison of two epochs (2000-2009 & 2010-2020) for survival until discharge for ELBW neonates analyzed based on country of origin.

Study or Subgroup	Events	Total	Weight	IV, Random, 95% CI
**2010-2020**				
SINGH 2020	1	9	0.4%	0.11 [0.00; 0.48]
OKELLA 2019	7	28	1.0%	0.25 [0.11; 0.45]
OKELLA 2019	14	36	1.1%	0.39 [0.23; 0.57]
RYLANCE 2013	8	71	1.1%	0.11 [0.05; 0.21]
SEID 2019	2	29	0.6%	0.07 [0.01; 0.23]
REZAEIZADEH 2018	69	127	1.4%	0.54 [0.45; 0.63]
SACKEY 2019	113	560	1.5%	0.20 [0.17; 0.24]
SERCE 2014	56	179	1.4%	0.31 [0.25; 0.39]
BAJAJ 2020	29	97	1.3%	0.30 [0.21; 0.40]
LUTHULI 2019	52	105	1.4%	0.50 [0.40; 0.59]
MILES 2017	31	63	1.3%	0.49 [0.36; 0.62]
MUHE 2019	28	165	1.4%	0.17 [0.12; 0.24]
SAINI 2016	2	17	0.6%	0.12 [0.01; 0.36]
ALI 2019	0	200	0.2%	0.00 [0.00; 0.02]
ABDUL–MUMIN 2020	3	21	0.7%	0.14 [0.03; 0.36]
ADHIKARI 2017	26	38	1.1%	0.68 [0.51; 0.82]
AHLSEN 2015	3	45	0.7%	0.07 [0.01; 0.18]
ALI 2016	43	113	1.4%	0.38 [0.29; 0.48]
AMADI 2015	2	22	0.6%	0.09 [0.01; 0.29]
ANDEGIORGISH 2020	13	22	1.0%	0.59 [0.36; 0.79]
ATALAY 2013	8	20	0.9%	0.40 [0.19; 0.64]
BALLOT 2017	224	546	1.5%	0.41 [0.37; 0.45]
BASIRI 2015	11	64	1.2%	0.17 [0.09; 0.29]
BOKADE 2018	17	47	1.2%	0.36 [0.23; 0.51]
CETINKAYA 2014	102	197	1.5%	0.52 [0.45; 0.59]
KIDAMBA 2018	5	15	0.8%	0.33 [0.12; 0.62]
LIN 2015	129	258	1.5%	0.50 [0.44; 0.56]
SUN 2013	10	28	1.0%	0.36 [0.19; 0.56]
TSHEHLA 2019	36	71	1.3%	0.51 [0.39; 0.63]
TAMENE 2020	0	23	0.2%	0.00 [0.00; 0.15]
THAKUR 2013	173	283	1.5%	0.61 [0.55; 0.67]
TOSIF 2019	10	45	1.1%	0.22 [0.11; 0.37]
TRAN 2015	1	26	0.4%	0.04 [0.00; 0.20]
NTULI 2020	26	50	1.2%	0.52 [0.37; 0.66]
WINKLER 2020	3	8	0.6%	0.38 [0.09; 0.76]
WU 2019	598	1303	1.5%	0.46 [0.43; 0.49]
ZHANG 2020	68	100	1.4%	0.68 [0.58; 0.77]
ZUNIGA 2013	1	11	0.4%	0.09 [0.00; 0.41]
SAHOO 2020	143	231	1.5%	0.62 [0.55; 0.68]
MEKASHA 2020	28	164	1.4%	0.17 [0.12; 0.24]
GUPTA 2020	15	49	1.2%	0.31 [0.18; 0.45]
HAGHIGHI 2013	11	52	1.1%	0.21 [0.11; 0.35]
CHIDIEBERE 2018	4	20	0.8%	0.20 [0.06; 0.44]
NEPAL 2020	0	3	0.2%	0.00 [0.00; 0.71]
NEVACINOVIC 2020	21	49	1.2%	0.43 [0.29; 0.58]
MONTANO PEREZ 2019	20	52	1.2%	0.38 [0.25; 0.53]
Total (95% CI)		5662	48.0%	0.33 [0.28; 0.38]
**2000-2001**				
SEHGAL 2004	30	52	1.2%	0.58 [0.43; 0.71]
PINHEIRO 2010	146	445	1.5%	0.33 [0.28; 0.37]
PERVIN 2015	1	15	0.4%	0.07 [0.00; 0.32]
ROY 2006	24	36	1.1%	0.67 [0.49; 0.81]
RUIZ–PELAEZ 2014	40	86	1.4%	0.47 [0.36; 0.58]
POUDEL 2010	8	23	1.0%	0.35 [0.16; 0.57]
OMOIGBERALE 2009	99	151	1.4%	0.66 [0.57; 0.73]
ALTAMEEMI 2019	7	32	1.0%	0.22 [0.09; 0.40]
ONALO 2015	11	42	1.1%	0.26 [0.14; 0.42]
MARTINEZ 2010	54	152	1.4%	0.36 [0.28; 0.44]
OSORNO–COVARRUBIAS 2002	78	162	1.4%	0.48 [0.40; 0.56]
GOLESTAN 2008	2	35	0.6%	0.06 [0.01; 0.19]
MUKHOPADHYAY 2013	134	436	1.5%	0.31 [0.26; 0.35]
NAVAEI 2010	25	107	1.3%	0.23 [0.16; 0.33]
SAUCEDO 2008	334	727	1.5%	0.46 [0.42; 0.50]
AFJEH 2013A	49	147	1.4%	0.33 [0.26; 0.42]
AHMETI 2010	0	73	0.2%	0.00 [0.00; 0.05]
AZEREDO CARDOSO 2013	151	305	1.5%	0.50 [0.44; 0.55]
BALLOT 2010	50	143	1.4%	0.35 [0.27; 0.43]
BALLOT 2012	28	95	1.3%	0.29 [0.21; 0.40]
BASU 2008	16	45	1.2%	0.36 [0.22; 0.51]
CARNEIRO 2012	27	57	1.3%	0.47 [0.34; 0.61]
DE BRITO 2003	38	79	1.3%	0.48 [0.37; 0.60]
DE MELLO 2007	28	60	1.3%	0.47 [0.34; 0.60]
ATASAY 2003	16	31	1.1%	0.52 [0.33; 0.70]
DE ARAÚJO 2007	19	61	1.3%	0.31 [0.20; 0.44]
KAREEM 2011	17	48	1.2%	0.35 [0.22; 0.51]
HENDRIKS 2014	9	97	1.1%	0.09 [0.04; 0.17]
KALIMBA 2013	101	382	1.5%	0.26 [0.22; 0.31]
SRITIPSUKHO 2007	11	21	1.0%	0.52 [0.30; 0.74]
OGUNLESI 2011	1	15	0.4%	0.07 [0.00; 0.32]
NEOCOSUR 2002	60	126	1.4%	0.48 [0.39; 0.57]
BUENOS AIRES 2012	182	451	1.5%	0.40 [0.36; 0.45]
TROTMAN 2012	96	286	1.5%	0.34 [0.28; 0.39]
TROTMAN 2007	20	47	1.2%	0.43 [0.28; 0.58]
UGWU 2010	6	149	1.0%	0.04 [0.01; 0.09]
VELAPHI 2005	186	453	1.5%	0.41 [0.36; 0.46]
NNPD 2004	21	101	1.3%	0.21 [0.13; 0.30]
WELBECK 2003	21	128	1.3%	0.16 [0.10; 0.24]
ZHANG 2011	18	39	1.2%	0.46 [0.30; 0.63]
GOODEN 2013	4	46	0.8%	0.09 [0.02; 0.21]
CHIABI 2014	3	74	0.8%	0.04 [0.01; 0.11]
KLINGENBERG 2003	5	11	0.7%	0.45 [0.17; 0.77]
CASTRO 2007	55	270	1.4%	0.20 [0.16; 0.26]
Total (95% CI)		6341	52.0%	0.33 [0.29; 0.37]
**Total (95% CI)**		**12003**	**100.0%**	**0.33 [0.30; 0.36]**

Test of moderators - Not significant

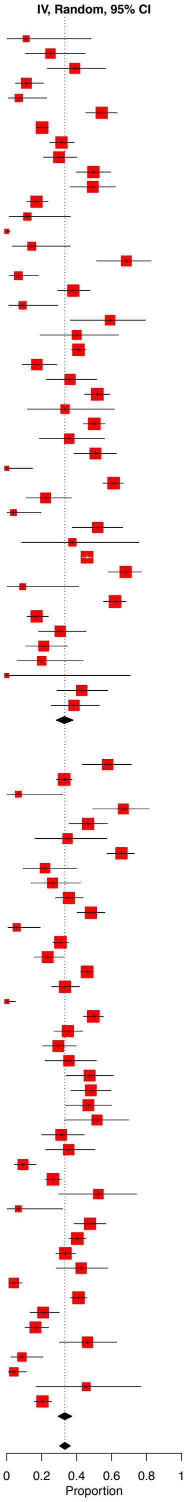

Supplement Figure 52: Sensitivity analysis - primary outcome - Comparison of two epochs (2000-2009 & 2010-2020) for survival until discharge for ELGANs analyzed based on country of origin.

Study or Subgroup	Events	Total	Weight	IV, Random, 95% CI
**2000-2009**				
SEHGAL 2004	4	9	1.4%	0.44 [0.14; 0.79]
PERVIN 2015	2	14	1.2%	0.14 [0.02; 0.43]
ROY 2006	7	14	1.6%	0.50 [0.23; 0.77]
RUIZ−PELAEZ 2014	43	81	2.1%	0.53 [0.42; 0.64]
POUDEL 2010	2	9	1.2%	0.22 [0.03; 0.60]
OMOIGBERALE 2009	128	190	2.2%	0.67 [0.60; 0.74]
OSORNO−COVARRUBIAS 2002	11	50	1.9%	0.22 [0.12; 0.36]
MUKHOPADHYAY 2013	19	169	2.1%	0.11 [0.07; 0.17]
NAVAEI 2010	29	122	2.1%	0.24 [0.17; 0.32]
ADINMA 2013	0	2	0.5%	0.00 [0.00; 0.84]
AFJEH 2013	72	196	2.2%	0.37 [0.30; 0.44]
BASU 2008	11	46	1.9%	0.24 [0.13; 0.39]
DE BRITO 2003	30	70	2.1%	0.43 [0.31; 0.55]
DE MELLO 2007	24	51	2.0%	0.47 [0.33; 0.62]
ATASAY 2003	24	37	1.9%	0.65 [0.47; 0.80]
DE ARAÚJO 2007	8	30	1.8%	0.27 [0.12; 0.46]
DECARVALHO 2007	34	108	2.1%	0.31 [0.23; 0.41]
KIRSTEN 2012	231	309	2.2%	0.75 [0.70; 0.80]
NEOCOSUR 2002	43	95	2.1%	0.45 [0.35; 0.56]
GORDANA 2019	141	220	2.2%	0.64 [0.57; 0.70]
WELBECK 2003	176	382	2.2%	0.46 [0.41; 0.51]
ZHANG 2011	11	29	1.9%	0.38 [0.21; 0.58]
YAKOOB 2014	11	38	1.9%	0.29 [0.15; 0.46]
QAZI 2011	0	3	0.5%	0.00 [0.00; 0.71]
CHIABI 2014	6	75	1.8%	0.08 [0.03; 0.17]
Total (95% CI)		2349	45.6%	0.37 [0.29; 0.46]
**2010-2020**				
PRADHAN 2019	5	18	1.6%	0.28 [0.10; 0.53]
SEID 2019	2	25	1.3%	0.08 [0.01; 0.26]
REZAEIZADEH 2018	40	85	2.1%	0.47 [0.36; 0.58]
SACKEY 2019	141	539	2.2%	0.26 [0.22; 0.30]
SERCE 2014	54	156	2.2%	0.35 [0.27; 0.43]
LI 2019	159	307	2.2%	0.52 [0.46; 0.58]
LUTHULI 2019	14	53	2.0%	0.26 [0.15; 0.40]
MILES 2017	2	5	1.0%	0.40 [0.05; 0.85]
MUHE 2019	15	104	2.0%	0.14 [0.08; 0.23]
ABDUL−MUMIN 2020	4	20	1.6%	0.20 [0.06; 0.44]
BASIRI 2015	24	68	2.1%	0.35 [0.24; 0.48]
KIDAMBA 2018	5	15	1.6%	0.33 [0.12; 0.62]
JIANG 2020	235	320	2.2%	0.73 [0.68; 0.78]
KULALI 2019	46	165	2.2%	0.28 [0.21; 0.35]
KONG 2020	35	50	2.0%	0.70 [0.55; 0.82]
KONG 2016	101	148	2.2%	0.68 [0.60; 0.76]
SUN 2013	7	32	1.8%	0.22 [0.09; 0.40]
TAMENE 2020	0	15	0.6%	0.00 [0.00; 0.22]
THAKUR 2013	28	81	2.1%	0.35 [0.24; 0.46]
TRAN 2015	2	29	1.3%	0.07 [0.01; 0.23]
NTULI 2020	9	25	1.8%	0.36 [0.18; 0.57]
UNDELA 2019	2	6	1.1%	0.33 [0.04; 0.78]
XU 2019	101	148	2.2%	0.68 [0.60; 0.76]
ZHANG 2020	20	32	1.9%	0.62 [0.44; 0.79]
ZHOU 2014	50	72	2.1%	0.69 [0.57; 0.80]
ZHU 2020	308	441	2.2%	0.70 [0.65; 0.74]
SAHOO 2020	43	95	2.1%	0.45 [0.35; 0.56]
GUPTA 2020	8	30	1.8%	0.27 [0.12; 0.46]
CHIOUKH 2018	27	109	2.1%	0.25 [0.17; 0.34]
NEPAL 2020	1	2	0.6%	0.50 [0.01; 0.99]
Total (95% CI)		3195	54.4%	0.39 [0.31; 0.47]
**Total (95% CI)**		**5544**	**100.0%**	**0.38 [0.32; 0.44]**

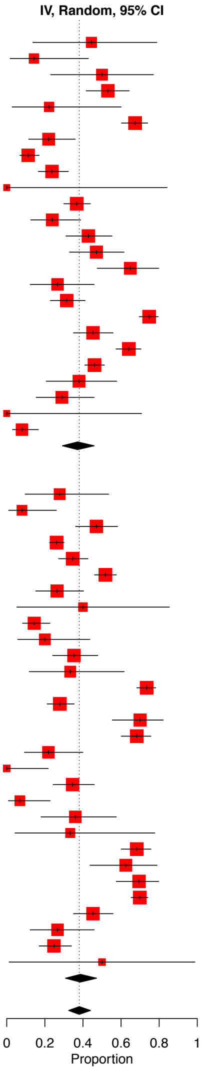

Test of moderators - Not significant

Supplement Table 1: Literature search strategy

MEDLINE

#	Searches	Results
1	Infant, Premature/	54373
2	Infant, Low Birth Weight/	18637
3	Infant, Extremely Premature/	2650
4	Infant, Very Low Birth Weight/	8594
5	(Preterm* or premature or "Low birth weight" or lbw or vlbw or elbw or "Low birth weights" or "Low-birth-weight" or "low-birth-weights Low birthweight" or "Low birthweights" or "pre-terms" or "Pre-term" or "Extremely premature").ab,ti.	203611
6	1 or 2 or 3 or 4 or 5	224386
7	Morbidity/	30480
8	Mortality/	45104
9	Survival/	4750
10	Risk/	123509
11	Developmental Disabilities/ or Neurodevelopmental Disorders/	22698
12	(risk* or morbidit* or mortalit* or surviv* or died or death* or dead* or outcome* or neurodevelop* or developmen*).ab,ti.	7142150
13	7 or 8 or 9 or 10 or 11 or 12	7195266
14	Developing Countries/	75481
15	(Afghanistan or Benin or "Burkina Faso" or Burundi or "Central African Republic" or Guinea or Chad or Comoros or "Congo Dem Rep" or Congo or Eritrea or Ethiopia or Gambia or "Guinea Bissau" or Haiti or Korea or "Dem Peoples Rep" or Somalia or Liberia or Madagascar or Malawi or Mali or Mozambique or Nepal or Niger or Rwanda or Senegal or "Sierra Leone" or "South Sudan" or Tanzania or Togo or Uganda or Zimbabwe).ab,ti.	281521

16	(Angola or Armenia or Bangladesh or Bhutan or Bolivia or "Cabo Verde" or Cambodia or Cameroon or "Cote d'Ivoire" or Djibouti or Egypt or "El Salvador" or Georgia or Ghana or Guatemala or Honduras or India or Indonesia or Jordan or Kenya or Kiribati or Kosovo or "Kyrgyz Republic" or "Lao PDR" or Lao or Lesotho or Mauritania or Micronesia or "Fed Sts" or Moldova or Mongolia or Morocco or Myanmar or Nicaragua or Nigeria or Pakistan or "Papua New Guinea" or Philippines or "Sao Tome" or Principe or "Solomon Islands" or "Sri Lanka" or Sudan or Swaziland or "Syrian Arab Republic" or syria or Tajikistan or "Timor Leste" or Tunisia or Ukraine or Uzbekistan or Vanuatu or Vietnam or "West Bank Gaza" or Yemen or Zambia).ab,ti.	320980
17	(Albania or Algeria or "American Samoa" or Argentina or Azerbaijan or Belarus or Belize or Bosnia or Herzegovina or Botswana or Brazil or Bulgaria or China or Colombia or "Costa Rica ORCroatia" or Cuba or Dominica or "Dominica Republic" or Ecuador or "Equatorial Guinea" or Fiji or Gabon or Grenada or Guyana or Iran or "Islamic Rep" or Iraq or Jamaica or Kazakhstan or Lebanon or Libya or Macedonia or FYR Malaysia or Maldives or "Marshall Islands" or Mauritius or Mexico or Montenegro or Namibia or Nauru or Panama or Paraguay or Peru or Romania or "Russian Federation" or Samoa or Serbia or "South Africa" or "St. Lucia" or "St. Vincent" or Grenadines or Suriname or Thailand or Tonga or Turkey or Turkmenistan or Tuvalu or Venezuela).ab,ti.	545809
18	("middle income countries" or "low income countries" or LMIC or "developing countries" OR "undeveloped countries" or "under developed countries" or "south asia" or "south Asian" or Asia or "middle income countries" or "resource limited" or Africa or Southeastern or "Pacific Islands" or "Micronesia" or "Middle East" or "South America" or (("Low income" or "Lower middle income" or "Upper middle income") adj3 (countr* or econom*))).ab,ti.	231604
19	14 or 15 or 16 or 17 or 18	1240831

20	6 and 13 and 19	10305
21	limit 20 to yr="2000 -Current"	9041

EMBASE

#	Searches	Results
1	Infant, Premature/	99778
2	Infant, Low Birth Weight/	32922
3	Infant, Extremely Premature/	104816
4	Infant, Very Low Birth Weight/	12579
5	(Preterm* or premature or "Low birth weight" or lbw or vlbw or elbw or "Low birth weights" or "Low-birth-weight" or "low-birth-weights Low birthweight" or "Low birthweights" or "pre-terms" or "Pre-term" or "Extremely premature").ab,ti.	271015
6	1 or 2 or 3 or 4 or 5	307247
7	Morbidity/	351146
8	Mortality/	765117
9	Survival/	309766
10	Risk/	501486
11	Developmental Disabilities/ or Neurodevelopmental Disorders/	185762
12	(risk* or morbidit* or mortalit* or surviv* or died or death* or dead* or outcome* or neurodevelop* or developmen*).ab,ti.	9699888
13	7 or 8 or 9 or 10 or 11 or 12	10026886
14	Developing Countries/	84094
15	(Afghanistan or Benin or "Burkina Faso" or Burundi or "Central African Republic" or Guinea or Chad or Comoros or "Congo Dem Rep" or Congo or Eritrea or Ethiopia or Gambia or "Guinea Bissau" or Haiti or Korea or "Dem Peoples Rep" or Somalia or Liberia or Madagascar or Malawi or Mali or Mozambique or Nepal or Niger or Rwanda or Senegal or "Sierra Leone" or "South Sudan" or Tanzania or Togo or Uganda or Zimbabwe).ab,ti.	318361

16	(Angola or Armenia or Bangladesh or Bhutan or Bolivia or "Cabo Verde" or Cambodia or Cameroon or "Cote d’Ivoire" or Djibouti or Egypt or "El Salvador" or Georgia or Ghana or Guatemala or Honduras or India or Indonesia or Jordan or Kenya or Kiribati or Kosovo or "Kyrgyz Republic" or "Lao PDR" or Lao or Lesotho or Mauritania or Micronesia or "Fed Sts" or Moldova or Mongolia or Morocco or Myanmar or Nicaragua or Nigeria or Pakistan or "Papua New Guinea" or Philippines or "Sao Tome" or Principe or "Solomon Islands" or "Sri Lanka" or Sudan or Swaziland or "Syrian Arab Republic" or syria or Tajikistan or "Timor Leste" or Tunisia or Ukraine or Uzbekistan or Vanuatu or Vietnam or "West Bank Gaza" or Yemen or Zambia).ab,ti.	411885
17	(Albania or Algeria or "American Samoa" or Argentina or Azerbaijan or Belarus or Belize or Bosnia or Herzegovina or Botswana or Brazil or Bulgaria or China or Colombia or "Costa Rica ORCroatia" or Cuba or Dominica or "Dominica Republic" or Ecuador or "Equatorial Guinea" or Fiji or Gabon or Grenada or Guyana or Iran or "Islamic Rep" or Iraq or Jamaica or Kazakhstan or Lebanon or Libya or Macedonia or FYR Malaysia or Maldives or "Marshall Islands" or Mauritius or Mexico or Montenegro or Namibia or Nauru or Panama or Paraguay or Peru or Romania or "Russian Federation" or Samoa or Serbia or "South Africa" or "St. Lucia" or "St. Vincent" or Grenadines or Suriname or Thailand or Tonga or Turkey or Turkmenistan or Tuvalu or Venezuela).ab,ti.	686270
18	("middle income countries" or "low income countries" or LMIC or "developing countries” OR “undeveloped countries" or "under developed countries" or "south asia" or "south Asian" or Asia or "middle income countries" or "resource limited" or Africa or Southeastern or "Pacific Islands" or "Micronesia" or "Middle East" or "South America" or (("Low income" or "Lower middle income" or "Upper middle income") adj3 (countr* or econom*))).ab,ti.	289763
19	14 or 15 or 16 or 17 or 18	1543787

20	6 and 13 and 19	15218
21	limit 20 to yr="2000 -Current"	14094
22	limit 21 to embase	8523

COCHRANE

ID	Search	Hit
#1	MeSH descriptor: [Infant, Premature] explode all trees	3752
#2	MeSH descriptor: [Infant, Low Birth Weight] explode all trees	2178
#3	(Preterm* or premature or "Low birth weight" or lbw or vlbw or elbw or "Low birth weights" or "Low birthweight" or "Low birthweights" or "pre-terms" or "Pre-term" or "Extremely premature"):ti,ab,kw (Word variations have been searched)	30019
#4	#1 or #3	30019
#5	Afghanistan or Benin or "Burkina Faso" or Burundi or "Central African Republic" or Guinea or Chad or Comoros or "Congo Dem Rep" or Congo or Eritrea or Ethiopia or Gambia or "Guinea Bissau" or Haiti or Korea or "Dem Peoples Rep" or Somalia or Liberia or Madagascar or Malawi or Mali or Mozambique or Nepal or Niger or Rwanda or Senegal or "Sierra Leone" or "South Sudan" or Tanzania	30585
#6	Angola or Armenia or Bangladesh or Bhutan or Bolivia or "Cabo Verde" or Cambodia or Cameroon or "Cote d'Ivoire" or Djibouti or Egypt or "El Salvador" or Georgia or Ghana or Guatemala or Honduras or India or Indonesia or Jordan or Kenya or Kiribati or Kosovo or "Kyrgyz Republic" or "Lao PDR" or Lao or Lesotho or Mauritania or Micronesia or "Fed Sts" or Moldova or Mongolia or Morocco or Myanmar or Nicaragua or Nigeria or Pakistan or "Papua New Guinea" or Philippines or "Sao Tome" or Principe or "Solomon Islands" or "Sri Lanka" or Sudan or Swaziland or "Syrian Arab Republic" or syria or Tajikistan or "Timor Leste" or Tunisia or Ukraine or Uzbekistan or Vanuatu or Vietnam or "West Bank Gaza"	57183
#7	Albania or Algeria or "American Samoa" or Argentina or Azerbaijan or Belarus or Belize or Bosnia or Herzegovina or Botswana or Brazil or Bulgaria or China or Colombia or "Costa Rica ORCroatia" or Cuba or Dominica or "Dominica Republic" or Ecuador or "Equatorial Guinea" or Fiji or Gabon or Grenada or Guyana or Iran or "Islamic Rep" or Iraq or Jamaica or Kazakhstan or Lebanon or Libya or Macedonia or FYR Malaysia or Maldives or "Marshall Islands" or Mauritius or Mexico or Montenegro or Namibia or Nauru or Panama or Paraguay or Peru or Romania or "Russian Federation" or Samoa or Serbia or "South Africa" or "St. Lucia" or "St. Vincent" or Grenadines or Suriname or Thailand or Tonga or	131036

#8	(Africa or Asia or Caribbean or "West Indies" or "South America" or "Latin America" or "Central America")	19479
#9	((developing or "less* developed" or "under developed" or underdeveloped or "middle income" or "low* income" or underserved or "under served" or deprived or poor*) NEAR (countr*	10590
#10	((developing or "less* developed" or "under developed" or underdeveloped or "middle income" or "low* income") NEXT	41
#11	low NEXT (GDP or GNP or "gross domestic" or "gross national")	49
#12	(LMIC or LMICs or "third world" or "LAMI country" or "LAMI countries")	603
#13	("transitional country" or "transitional countries")	23
#14	{OR #5-#13}	218156
#15	#4 AND #14 with Cochrane Library publication date from Jan 2000 to present	4635
	**Review 459, trial from CT.gov 340, ICTRP 99 and CINAHL 65**	

Web of Science

ID	Hit	Search
# 26	3,008	#24 AND #21<br>Refined by: PUBLICATION YEARS: (2020 OR 2006 OR 2019 OR 2005 OR 2018 OR 2004 OR 2017 OR 2003 OR 2016 OR 2002 OR 2015 OR 2001 OR 2014 OR 2000 OR 2013 OR 2012 OR 2011 OR 2010 OR 2009 OR 2008 OR 2007)<br>*Databases= WOS, BCI, BIOSIS, DRCI, KJD, RSCI, SCIELO, ZOOREC Timespan=All years Search language=Auto*
# 25	3,327	#24 AND #21<br>*Databases= WOS, BCI, BIOSIS, DRCI, KJD, RSCI, SCIELO, ZOOREC Timespan=All years Search language=Auto*
# 24	27,668	#23 OR #22<br>*Databases= WOS, BCI, BIOSIS, DRCI, KJD, RSCI, SCIELO, ZOOREC Timespan=All years Search language=Auto*
# 23	22,797	AB=((extremely or very) NEAR/2 (preterm OR premature or "low birth"))<br>*Databases= WOS, BCI, BIOSIS, DRCI, KJD, RSCI, SCIELO, ZOOREC Timespan=All years Search language=Auto*

# 22	13,650	TI=((extremely or very) NEAR/2 (preterm OR premature or "low birth"))<br>*Databases= WOS, BCI, BIOSIS, DRCI, KJD, RSCI, SCIELO, ZOOREC Timespan=All years Search language=Auto*
# 21	37,871	#20 AND #17 AND #1<br>*Databases= WOS, BCI, BIOSIS, DRCI, KJD, RSCI, SCIELO, ZOOREC Timespan=All years Search language=Auto*
# 20	17,278,105	#19 OR #18<br>*Databases= WOS, BCI, BIOSIS, DRCI, KJD, RSCI, SCIELO, ZOOREC Timespan=All years Search language=Auto*
# 19	15,551,453	AB=(risk* or morbidit* or mortalit* or surviv* or died or death* or dead* or outcome* or neu rodevelop* or developmen*)<br>*Databases= WOS, BCI, BIOSIS, DRCI, KJD, RSCI, SCIELO, ZOOREC Timespan=All years Search language=Auto*
# 18	4,270,784	TI=(risk* or morbidit* or mortalit* or surviv* or died or death* or dead* or outcome* or neur odevelop* or developmen*)<br>*Databases= WOS, BCI, BIOSIS, DRCI, KJD, RSCI, SCIELO, ZOOREC Timespan=All years Search language=Auto*
# 17	8,642,556	#16 OR #15 OR #14 OR #13 OR #12 OR #11 OR #10 OR #9 OR #8 OR #7 OR #6 OR #5 O R #4 OR #3 OR #2<br>*Databases= WOS, BCI, BIOSIS, DRCI, KJD, RSCI, SCIELO, ZOOREC Timespan=All years Search language=Auto*
# 16	560	AB=("transitional country" or "transitional cou ntries")<br>*Databases= WOS, BCI, BIOSIS, DRCI, KJD, RSCI, SCIELO, ZOOREC Timespan=All years Search language=Auto*
# 15	150	Ti=("transitional country" or "transitional count ries")<br>*Databases= WOS, BCI, BIOSIS, DRCI, KJD, RSCI, SCIELO, ZOOREC Timespan=All years Search language=Auto*
# 14	33,770	AB=(LMIC or LMICs or "third world" or "LA MI country" or "LAMI countries")<br>*Databases= WOS, BCI, BIOSIS, DRCI, KJD, RSCI, SCIELO, ZOOREC Timespan=All years Search language=Auto*

# 13	8,432	TI=(LMIC or LMICs or "third world" or "LA MI country" or "LAMI countries") *Databases= WOS, BCI, BIOSIS, DRCI, KJD, RSCI, SCIELO, ZOOREC Timespan=All years Search language=Auto*
# 12	1,257	AB=(low NEAR/2 (GDP or GNP or "gross domestic" or "gross national")) *Databases= WOS, BCI, BIOSIS, DRCI, KJD, RSCI, SCIELO, ZOOREC Timespan=All years Search language=Auto*
# 11	39	TI=(low NEAR/2 (GDP or GNP or "gross domestic" or "gross national")) *Databases= WOS, BCI, BIOSIS, DRCI, KJD, RSCI, SCIELO, ZOOREC Timespan=All years Search language=Auto*
# 10	16,987	AB=((developing or "less* developed" or "und er developed" or underdeveloped or "middle in come" or "low* income") NEAR/3 (economy or economies)) *Databases= WOS, BCI, BIOSIS, DRCI, KJD, RSCI, SCIELO, ZOOREC Timespan=All years Search language=Auto*
# 9	2,986	TI=((developing or "less* developed" or "unde r developed" or underdeveloped or "middle inc ome" or "low* income") NEAR/3 (economy or economies)) *Databases= WOS, BCI, BIOSIS, DRCI, KJD, RSCI, SCIELO, ZOOREC Timespan=All years Search language=Auto*
# 8	3,279,059	AB=((developing or "less* developed" or "und er developed" or underdeveloped or "middle in come" or "low* income" or underserved or "un der served" or deprived or poor*) NEAR/ 3 (countr* or nation* or population* or world)) *Databases= WOS, BCI, BIOSIS, DRCI, KJD, RSCI, SCIELO, ZOOREC Timespan=All years Search language=Auto*
# 7	62,387	TI=((developing or "less* developed" or "unde r developed" or underdeveloped or "middle inc ome" or "low* income" or underserved or "und er served" or deprived or poor*) NEAR/ 3 (countr* or nation* or population* or world)) *Databases= WOS, BCI, BIOSIS, DRCI, KJD, RSCI, SCIELO, ZOOREC Timespan=All years Search language=Auto*

# 6	3,232,681	AB=(Africa or Asia or Caribbean or "West Indies" or "South America" or "Latin America" or " Central America")<br>*Databases= WOS, BCI, BIOSIS, DRCI, KJD, RSCI, SCIELO, ZOOREC Timespan=All years Search language=Auto*
# 5	395,886	TI=(Africa or Asia or Caribbean or "West Indies" or "South America" or "Latin America" or " Central America")<br>*Databases= WOS, BCI, BIOSIS, DRCI, KJD, RSCI, SCIELO, ZOOREC Timespan=All years Search language=Auto*
# 4	3,400,926	TS=(Albania or Algeria or "American Samoa" or Argentina or Azerbaijan or Belarus or Belize or Bosnia or Herzegovina or Botswana or Brazil or Bulgaria or China or Colombia or "Costa Rica ORCroatia" or Cuba or Dominica or "Dominica Republic" or Ecuador or "Equatorial Guinea" or Fiji or Gabon or Grenada or Guyana or Iran or "Islamic Rep" or Iraq or Jamaica or Kazakhstan or Lebanon or Libya or Macedonia or FYR Malaysia or Maldives or "Marshall Islands" or Mauritius or Mexico or Montenegro or Namibia or Nauru or Panama or Paraguay or Peru or Romania or "Russian Federation" or Samoa or Serbia or "South Africa" or "St. Lucia" or "St. Vincent" or Grenadines or Suriname or Thailand or Tonga or Turkey or Turkmenistan or Tuvalu or Venezuela)<br>*Databases= WOS, BCI, BIOSIS, DRCI, KJD, RSCI, SCIELO, ZOOREC Timespan=All years Search language=Auto*

# 3	1,853,053	TS= (Angola or Armenia or Bangladesh or Bhutan or Bolivia or "Cabo Verde" or Cambodia or Cameroon or "Cote d'Ivoire" or Djibouti or Egypt or "El Salvador" or Georgia or Ghana or Guatemala or Honduras or India or Indonesia or Jordan or Kenya or Kiribati or Kosovo or "Kyrgyz Republic" or "Lao PDR" or Lao or Lesotho or Mauritania or Micronesia or "Fed Sts" or Moldova or Mongolia or Morocco or Myanmar or Nicaragua or Nigeria or Pakistan or "Papua New Guinea" or Philippines or "Sao Tome" or Principe or "Solomon Islands" or "Sri Lanka" or Sudan or Swaziland or "Syrian Arab Republic" or syria or Tajikistan or "Timor Leste" or Tunisia or Ukraine or Uzbekistan or Vanuatu or Vietnam or "West Bank Gaza" or Yemen or Zambia) *Databases= WOS, BCI, BIOSIS, DRCI, KJD, RSCI, SCIELO, ZOOREC Timespan=All years Search language=Auto*
# 2	1,270,275	TS=(Afghanistan or Benin or "Burkina Faso" o r Burundi or "Central African Republic" or Gui nea or Chad or Comoros or "Congo Dem Rep" or Congo or Eritrea or Ethiopia or Gambia or " Guinea Bissau" or Haiti or Korea or "Dem Peo ples Rep" or Somalia or Liberia or Madagascar or Malawi or Mali or Mozambique or Nepal or Niger or Rwanda or Senegal or "Sierra Leone" or "South Sudan" or Tanzania or Togo or Ugan da or Zimbabwe) *Databases= WOS, BCI, BIOSIS, DRCI, KJD, RSCI, SCIELO, ZOOREC Timespan=All years Search language=Auto*
# 1	348,195	TS=(Preterm* or premature or "Low birth weig ht" or lbw or elbw or vlbw or "Low birth weigh ts" or "Low birthweight" or "Low birthweights " or "pre-terms" or "Pre- term" or "Extremely premature") *Databases= WOS, BCI, BIOSIS, DRCI, KJD, RSCI, SCIELO, ZOOREC Timespan=All years*

Supplement Table 2: Risk of bias of included studies

**Author/Year**	**Representativeness of the sample (★ If Attrition < 20% and if study is conducted after 2000)**	**Outcome defined clearly and assessed appropriately. Denominator defined clearly (★ If Both Yes)**	**All characteristics of patients known to affect the outcome reported? (Antenatal steroids, Gender, SGA Status, Apgar And Level of NICU) (★ If Minimum 3 Are Reported)**	**Data collection (★ If Prospective)**	**Overall Rob Assessment: ★★★★ - Low ★★★ Or ★★ - Intermediate ★ Or None - High**
Abdul-Mumin 2020	★	★	-	-	Intermediate
Adegoke 2014	★	★	-	★	Intermediate
Adhikari 2017	★	★	-	-	Intermediate
Adinma 2012	★	★	-	-	Intermediate
Afjeh 2013	★	★	-	-	Intermediate
Afjeh 2017	★	★	-	★	Intermediate
Aggarwal 2002	-	-	-	★	High
Ahlsen 2015	★	★	-	★	Intermediate
Ahmeti 2010	?	?	?	-	Unclear
Ali 2016	?	?	?	-	Unclear
Ali 2019	-	-	-	★	High
Alizadeh 2015	★	★	-	-	Intermediate
Altameemi 2019	-	-	-	?	High
Amadi 2015	-	-	-	-	High
Amadi 2019	-	-	-	★	High
Andegiorgish 2020	★	★	-	-	Intermediate
Araz-Ersan 2013	★	★	-	-	Intermediate
Arnold 2010	★	★	-	-	Intermediate
Atalay 2013	-	★	-	-	High
Atasay 2003	-	★	-	★	Intermediate
Azeredo Cardoso 2013	★	★	-	-	Intermediate
Bajaj 2020	★	★	-	-	Intermediate
Ballot 2010	★	★	-	-	Intermediate
Ballot 2012	-	★	-	★	Intermediate
Ballot 2017	★	★	-	★	Intermediate
Ballot 2017	-	-	-	★	High

Bas 2015	★	★	-	-	Intermediate
Bas 2018	-	-	-	★	High
Basiri 2015	-	★	-	★	Intermediate
Basu 2008	?	★	-	-	High
Buenos Aires 2012	★	★	-	★	Intermediate
Bhunwal 2017	★	★	-	★	Intermediate
Bokade 2018	★	★	-	★	Intermediate
Bolat 2012	★	★	-	★	Intermediate
Bonotto 2007	-	★	-	★	Intermediate
Boo 2012	★	★	-	-	Intermediate
Braimah 2020	-	★	-	★	Intermediate
Carneiro 2012	-	-	-	-	High
Castro 2007	★	★	-	★	Intermediate
Cauich-Aragon 2017	★	★	-	-	Intermediate
Cenk Celebi 2014	★	-	★	-	Intermediate
Cetinkaya 2014	★	★	★	-	Intermediate
Chaudhari 2009	★	-	-	★	Intermediate
Chen 2012	★	★	-	★	Intermediate
Chen 2015	-	★	-	★	Intermediate
Chen 2019	★	★	★	-	Intermediate
Chiabi 2014	★	★	-	-	Intermediate
Chidiebere 2018	★	-	★	★	Intermediate
Chiouk 2018	★	★	★	-	Intermediate
Coyles 2020	★	★	-	-	Intermediate
Dearaoujo 2007	-	-	-	-	High
Debritoa 2003	-	-	-	★	High
Decarvalho 2007	★	★	-	★	Intermediate
Demello 2007	-	★	-	-	High
Gebesce 2016	★	-	-	-	High
Gezmu 2020	-	★	-	★	Intermediate
Gharaibeh 2011	-	-	-	-	High
Ghaseminejad 2011	-	-	-	★	High
Golestan 2008	★	★	-	★	Intermediate
Goncalves 2014	★	★	-	★	Intermediate
Gooden 2013	★	★	-	-	Intermediate
Gordana 2019	★	★	-	-	Intermediate

Goulart 2011	-	★	★	-	Intermediate
Gupta 2020	★	★	-	★	Intermediate
Hadi 2013	-	-	-	-	High
Haghighi 2013	★	★	-	-	Intermediate
Hakeem 2012	★	★	-	★	Intermediate
Hendriks 2014	★	★	-	★	Intermediate
Ho 2001	-	★	-	★	Intermediate
Hussain 2020	-	★	-	★	Intermediate
Jiang 2020	★	★	-	★	Intermediate
Jirapaet 2010	-	★	★	★	Intermediate
Jodeiry 2012	-	-	-	★	High
Kalimba 2013	★	★	★	-	Intermediate
Karabulut 2019	-	★	★	-	Intermediate
Kareem 2011	-	-	-	-	High
Karkhaneh 2008	★	★	-	★	Intermediate
Kidamba 2018	★	★	-	★	Intermediate
Kirsten 2012	★	★	★	★	Low
Klingenberg 2003	★	-	-	★	Intermediate
Koksal 2002	★	★	-	★	Intermediate
Kong 2016	★	★	★	★	Low
Kong 2020	★	★	-	-	Intermediate
Kulali 2019	★	★	★	-	Intermediate
Lara-Molina 2013	-	-	-	-	High
Lermann 2006	★	★	-	★	Intermediate
Li 2018	-	★	-	★	Intermediate
Li 2019	★	★	-	-	Intermediate
Lin 2015	★	★	★	-	Intermediate
Liu 2005	-	★	-	-	High
Liu 2014	★	★	-	-	Intermediate
Lomuto 2010	★	★	-	-	Intermediate
Luthuli 2019	-	-	-	-	High
Mabhandi 2019	-	★	-	-	High
Martinez 2010	★	★	★	-	Intermediate
Martinez Cruz 2012	★	-	-	★	Intermediate
Mcgready 2018	-	★	-	-	High
Medina-Valenton 2016	★	-	-	★	Intermediate

Mekasha 2020	★	★	-	★	Intermediate
Miles 2017	★	★	-	★	Intermediate
Moghaddam 2015	★	★	★	-	Intermediate
Montano Perez 2019	★	★	-	-	Intermediate
Muhe 2019	★	★	-	★	Intermediate
Mukhopadhya y 2013	★	★	★	★	Low
Nakubulwa 2020	★	★	-	-	Intermediate
Navaei 2010	★	★	-	-	Intermediate
Neocosur 2002	-	★	-	★	Intermediate
Nepal 2020	★	★	-	-	Intermediate
Nevacinovic 2020	-	★	-	-	High
Nnpd 2004	★	★	-	★	Intermediate
Ntuli 2020	★	★	★	-	High
Ogunlesi 2011	-	★	-	★	Intermediate
Okello 2019	★	★	-	-	Intermediate
Omer 2014	-	-	-	★	High
Omoigberale 210	★	-	-	-	High
Onalo 2015	★	-	-	-	High
Oniriyuka 2010	★	★	-	★	Intermediate
Oommen 2019	★	★	★	★	Low
Osorno-Covarrubias	-	★	-	★	Intermediate
Ozcan 2015	-	-	-	-	High
Pervin 2015	★	★	-	★	Intermediate
Pinheiro 2010	-	★	-	-	High
Piriyapokin 2020	★	★	★	-	Intermediate
Poudel 2009	★	★	-	-	Intermediate
Pourarian 2016	★	★	-	★	Intermediate
Prabha 2014	★	★	-	-	Intermediate
Pradhan 2019	★	★	★	★	Low
Qazi 2011	★	★	-	★	Intermediate
Qian 2008	★	★	★	★	Low
Rezaeizadeh 2018	★	★	-	★	Intermediate
Roy 2006	★	★	★	-	Intermediate
Ruiz-Pelaez 2014	★	★	-	★	Intermediate
Rylance 2013	★	★	-	-	Intermediate
Sabzehei 2013	-	★	-	★	Intermediate

Saucedo 2008	★	★	-	-	Intermediate
Sackey 2019	★	★	-	-	Intermediate
Saeidi 2017	-	-	-	★	High
Sahin 2014	★	★	-	-	Intermediate
Sahoo 2020	★	★	★	★	Low
Saiedi 2009	-	-	-	★	High
Saini 2016	★	★	-	★	Intermediate
Salaluddin 2018	-	-	-	-	High
Saygili 2016	-	★	-	-	High
Sehgal 2004	-	★	-	★	Intermediate
Seid 2019	★	★	-	-	Intermediate
Serce 2014	★	★	-	-	Intermediate
Shrestha 2009	★	★	-	★	Intermediate
Singh 2020	★	★	-	★	Intermediate
Siswanto 2018	★	★	-	-	Intermediate
Sivanandan 2016	-	-	-	-	High
Sousa 2017	-	★	-	★	Intermediate
Sritipsukho 2007	★	★	-	-	Intermediate
Sun 2013	★	★	-	★	Intermediate
Tamene 2020	-	★	-	-	High
Taqui 2008	-	★	-	-	High
Thakre 2017	-	★	-	★	Intermediate
Thakur 2013	★	★	★	-	Intermediate
Tosif 2019	-	★	-	-	High
Tran 2015	★	★	-	★	Intermediate
Trotman 2006	-	★	-	-	High
Trotman 2007	-	-	★	-	High
Trotman 2007	-	★	-	-	High
Trotman 2012	-	★	-	-	High
Tshehla 2019	★	★	★	-	Intermediate
Ugwu 2010	★	★	-	-	Intermediate
Undela 2019	-	★	-	★	Intermediate
Velaphi 2005	-	★	-	-	High
Viau 2015	★	★	-	-	Intermediate
Vilanova 2019	★	★	-	-	Intermediate
Visserkift 2016	-	★	-	-	High

Vural 2007	-	★	-	★	Intermediate
Wang 2012	-	★	★	-	Intermediate
Welbeck 2003	-	-	-	-	High
Winkler 2020	-	★	-	-	High
Wu 2018	★	★	-	-	Intermediate
Wu 2019	-	★	★	-	Intermediate
Xu 2013	★	★	-	★	Intermediate
Xu 2019	★	★	-	-	Intermediate
Yadav 2019	★	★	-	★	Intermediate
Yakoob 2014	-	-	-	-	High
Yau 2014	★	★	★	-	Intermediate
Zea Vera 2019	-	-	-	-	High
Zepeda-Romero 2016	-	★	-	-	High
Zhang 2011	★	★	-	-	Intermediate
Zhang 2016	-	★	-	★	Intermediate
Zhang 2019	-	★	-	★	Intermediate
Zhang 2020	★	★	★	-	Intermediate
Zhou 2014	-	★	★	-	Intermediate
Zhu 2020	★	★	-	-	Intermediate
Ziadeh 2000	-	★	-	-	High
Ziylan 2006	-	★	-	-	High
Zuniga 2013	★	★	-	-	Intermediate

1.Abdul-Mumin A, Owusu SA, Abubakari A. Factors Associated with Treatment Outcome of Preterm Babies at Discharge from the Neonatal Intensive Care Unit (NICU) of the Tamale Teaching Hospital, Ghana. *Int J Pediatr*. 2020;2020:1–7.

2.Adegoke SA, Olugbemiga AO, Bankole KP, Tinuade OA. Intraventricular hemorrhage in newborns weighing <1500 g: Epidemiology and short-term clinical outcome in a resource-poor setting. *Ann Trop Med Public Heal*. 2014;7(1):48–54.

3.Adhikari S, Rao KS, B K G, Bahadur N. Morbidities and Outcome of a Neonatal Intensive Care in Western Nepal. *J Nepal Health Res Counc*. 2017;15(2):141–5.

4.Adinma ED. Maternal and perinatal outcome of eclampsia in tertiary health institution in Southeast Nigeria. *J Matern Neonatal Med*. 2013;26(2):211–4.

5.Afjeh SA, Sabzehei MK, Fallahi M, Esmaili F. Outcome of very low birth weight infants over 3 years report from an Iranian center. *Iran J Pediatr.* 2013;26(2):211-214.

6.Afjeh SA, Sabzehei MK, Shariati MK, Shamshiri AR, Esmaili F. Evaluation of initial respiratory support strategies in VLBW Neonates with RDS. *Arch Iran Med.* 2017;20(3):158–64.

7.Aggarwal R, Deorari AK, Azad R V., Kumar H, Talwar D, Sethi A, et al. Changing profile of retinopathy of prematurity. *J Trop Pediatr*. 2002;48(4):239–42.

8.Ahlsén AK, Spong E, Kafumba N, Kamwendo F, Wolff K. Born too small: Who survives in the public hospitals in Lilongwe, Malawi. *Arch Dis Child Fetal Neonatal Ed*. 2015;100(2):F150–4.

9.Ahmeti F, Azizi I, Hoxha S, Kulik-Rechberger B, Rechberger T. Mode of delivery and mortality among preterm newborns. *Ginekologia Polska.* 2010 Mar;81(3):203-207.

10.Celebi AR, Petricli IS, Hekimoglu E, Demirel N, Bas AY. The incidence and risk factors of severe retinopathy of prematurity in extremely low birth weight infants in Turkey. *Med Sci Monit.* 2014 Sep 15;20:1647-53.

11.Ali M, Rashid N, Khan MA, Anwar A. Pattern of Admission and Outcome of Low Birth Weight neonates in tertiary care hospital. *Pakistan J Med Heal Sci*. 2020;14(2):307–8.

12.Ali MA, Latif T, Islam MN, Hossain MA, Fakir HJ, Haque SA,et al. Status of Low Birth Weight Babies in Mymensingh Medical College Hospital. *Mymensingh Med J*. 2016;25(4):647-651.

13.Alizadeh Y, Zarkesh M, Moghadam RS, Esfandiarpour B, Behboudi H, Karambin MM, et al. Incidence and Risk Factors for Retinopathy of Prematurity in North of Iran. *J Ophthalmic Vis Res*. 2015;10(4):424-428.

14.Altameemi QDY, Ali MKM. Outcome of low birth weight infants in AL-zahraa teaching hospital in Kut; Iraq (Comparison between 2003 and 2008). *Indian J Public Heal Res Dev*. 2019;10(6):611–5.

15.Amadi HO, Okonkwo IR, Abioye IO, Abubakar AL, Olateju EK, Adesina CT,et al. A new low-cost commercial bubble CPAP (bCPAP) machine compared with a traditional bCPAP device in Nigeria. *Paediatr Int Child Health*. 2019;39(3):184–92.

16. Amadi HO, Olateju EK, Alabi P, Kawuwa MB, Ibadin MO, Osibogun AO. Neonatal hyperthermia and thermal stress in low-and middle-income countries: A hidden cause of death in extremely low-birthweight neonates. *Paediatr Int Child Health* 2015;35(3):273–81.

17.Andegiorgish AK, Andemariam M, Temesghen S, Ogbai L, Ogbe Z, Zeng L. Neonatal mortality and associated factors in the specialized neonatal care unit Asmara, Eritrea. *BMC Public Health*. 2020;20(1):1–9.

18.Pinheiro CE, Peres MA, D' Orsi E. Increased survival among lower-birthweight children in Southern Brazil. *Rev Saude Publica*. 2010;44(5):776-784.

19.Araújo BF, Tanaka AC. Risk factors associated with very low birth weight in a low-income population. *Cad Saude Publica*. 2007 Dec;23(12):2869-77.

20.Araz-Ersan B, Kir N, Akarcay K, Aydinoglu-Candan O, Sahinoglu-Keskek N, Demirel A, et al. Epidemiological analysis of retinopathy of prematurity in a referral centre in Turkey. *Br J Ophthalmol*. 2013;97(1):15–7.

21.Arnold M, Moore SW, Sidler D, Kirsten GF. Long-term outcome of surgically managed necrotizing enterocolitis in a developing country. *Pediatr Surg Int*. 2010;26(4):355–60.

22.Atalay D, Salihoğlu O, Can E, Beşkardeş A, Hatipoğlu S. Short-term outcomes of very low birth weight infants born at a tertiary care hospital, istanbul, Turkey. *Iran J Pediatr*.2013;23(2):205-211.

23.Atasay B, Günlemez A, Ünal S, Arsan S. Outcomes of very low birth weight infants in a newborn tertiary center in Turkey, 1997-2000. *Turk J Pediatr*.2003;45(4):283–9.

24.Bajaj M, Sharma J, Mahajan S, Sharma M, Sharma P. To retrospectively review and assess the survival rate of newborns admitted over 3 years to a Newborn Intensive Care Unit at a Tertiary Care Institute in Northern India. *J Clin Neonatol*. 2020;9(4):266.

25.Ballot DE, Agaba F, Cooper PA, Davies VA, Ramdin T, Chirwa L, et al. A review of delivery room resuscitation in very low birth weight infants in a middle income country. *Matern Heal Neonatol Perinatol*. 2017;3(1):1–8.

26.Ballot DE, Chirwa TF, Cooper PA. Determinants of survival in very low birth weight neonates in a public sector hospital in Johannesburg. *BMC Pediatr*. 2010;10:1–11.

27.Ballot DE, Potterton J, Chirwa T, Hilburn N, Cooper PA. Developmental outcome of very low birth weight infants in a developing country. BMC Pediatr. 2012 Feb 1;12:11

28.Ballot DE, Ramdin T, Rakotsoane D, Agaba F, Chirwa T, Davies VA, et al. Assessment of developmental outcome in very low birth weight infants in Southern Africa using the Bayley Scales of Infant Development (III). *BMJ Paediatr Open*. 2017;1(1):1–7.

29.Bas AY, Demirel N, Koc E, Ulubas Isik Di, Hirfanoglu IM, Tunc T. Incidence, risk factors and severity of retinopathy of prematurity in Turkey (TR-ROP study): A prospective, multicentre study in 69 neonatal intensive care units. *Br J Ophthalmol*. 2018;102(12):1711–6.

30.Bas AY, Koc E, Dilmen U, Oguz SS, Ovali F, Demirel N, et al. Incidence and severity of retinopathy of prematurity in Turkey. *Br J Ophthalmol*. 2015;99(10):1311–4.

31.Basiri B, Ashari FE, Shokouhi M, Sabzehei MK. Neonatal mortality and its main determinants in premature infants hospitalized in neonatal intensive care unit in Fatemieh hospital, Hamadan, *Iran. J Compr Pediatr*. 2015;6(3):e26965

32.Basu S, Rathore P, Bhatia BD. Predictors of mortality in very low birth weight neonates in India. *Singapore Med J*. 2008;49(7):556–60.

33.Bhunwal S, Mukhopadhyay K, Bhattacharya S, Dey P, Dhaliwal LK. Bronchopulmonary Dysplasia in Preterm Neonates in a Level III Neonatal Unit in India. *Indian Pediatr*. 2018;55(3): 211–5.

34.Bokade C, Meshram R. Morbidity and mortality patterns among outborn referral neonates in central India: Prospective observational study. *J Clin Neonatol*. 2018;7(3):130.

35.Bolat F, Uslu S, Bolat G, Comert S, Can E, Bulbul A, et al. Healthcare-associated infections in a neonatal intensive care unit in Turkey. *Indian Pediatr*. 2012;49(12):951–7.

36.Bonotto, Beatriz L, Moreira, Ramos AT, Carvalho, Siqueira D. Prevalence of retinopathy of prematurity in premature babies examined during the period 1992-1999, Joinville (SC): Evaluation of associated risks - Screening. *Arquivos Brasileiros de Oftalmologia. 2007;70*(1):55-61.

37.Boo NY, Cheah IGS. Risk factors associated with necrotising enterocolitis in very low birth weight infants in Malaysian neonatal intensive care units. *Singapore Med J*. 2012;53(12):826–31.

38.Boshoff Coyles L, Joolay Y, Tooke L. Bovine or Porcine: Does the Type of Surfactant Matter? *J Trop Pediatr.* 2020;66(5):534–41.

39.Braimah IZ, Enweronu-Laryea C, Sackey AH, Kenu E, Agyabeng K, Ofori-Adjei ID et al. Incidence and risk factors of retinopathy of prematurity in Korle-Bu Teaching Hospital: A baseline prospective study. *BMJ Open*. 2020;10(8).

40.Cardoso RCA, Flores PVG, Vieira CL, Bloch KV, Pinheiro RS, Fonseca SC, et al. Infant mortality in a very low birth weight cohort from a public hospital in Rio de Janeiro, RJ, Brazil. *Rev Bras Saude Matern Infant*. 2013;13(3):237–46.

41.Carneiro JA, Vieira MM, Reis TC, Caldeira AP. Risk factors for mortality of very low birth weight newborns at a neonatal intensive care unit. *Rev Paul Pediatr*. 2012;30(3):369–76.

42.Carvalho ABR de, Brito ÂSJ de, Matsuo T. Health care and mortality of very low birth weight newborns. *Revista de Saúde Pública* , *41* (6), 1003-1012. 2007;41(6):1003–12.

43.Castro, Eveline C. M, Leite, Álvaro J. M. Hospital mortality rates of infants with birth weight less than or equal to 1,500 g in the northeast of Brazil. *Jornal de Pediatria*, *83*(1), 27-32. J Pediatr (Rio J). 2007;83(1):27–32.

44.Cetinkaya M, Ercan TE, Saglam OK, Buyukkale G, Kavuncuoglu S, Mete F. Efficacy of prophylactic fluconazole therapy in decreasing the incidence of Candida infections in extremely low birth weight preterm infants. *Am J Perinatol*. 2014;31(12):1043-1048.

45.Chaudhari S, Patwardhan V, Vaidya U, Kadam S, Kamat A. Retinopathy of prematurity in a tertiary care center-Incidence, risk factors and outcome. *Indian Pediatr*. 2009;46(3):219–24.

46.Chen HJ, Wei KL, Zhou C Le, Yao YJ, Yang YJ, Fan XF, et al. Incidence of brain injuries in premature infants with gestational age ^{2}34 weeks in ten urban hospitals in China. *World J Pediatr.* 2013;9(1):17–24.

47.Chen X, Li H, Qiu X, Yang C, Walther FJ. Neonatal hematological parameters and the risk of moderate-severe bronchopulmonary dysplasia in extremely premature infants. *BMC Pediatr*. 2019;19(1):1–7.

48.Chen YH, Lien RI, Tsai S, Chang CJ, Lai CC, Chao AN, et al. Natural history of retinopathy of prematurity: Two-year outcomes of a prospective study. *Retina*. 2015;35(1):141–8.

49.Chiabi A, Mah E, Ntsama Essomba MJ, Nguefack S, Mbonda E, Tchokoteu PF. Factors associated with the survival of very low birth weight neonates at the Yaounde gynaeco-obstetric and pediatric hospital, Cameroon. *Arch Pediatr.* 2014;21(2):142–6.

50.Chidiebere ODI, Uchenna E, Christian I, Nwabueze AI, Stephen AO, Ifeyinwa O. The Low-birth weight Infants: Pattern of Morbidity and Mortality in a Tertiary Healthcare Facility in the South Eastern Nigeria. *Ann Med Health Sci Res.* 2018;8:4-10

51.Dautović GV, Doronjski A, Đurić GV. Incidence of bronchopulmonary dysplasia and mortality of very low birth weight infants in Vojvodina. *Srp Arh Celok Lek.*2020;148(1-2):52-57

52.Brito AS, Matsuo T, Gonzalez MR, de Carvalho AB, Ferrari LS. CRIB score, birth weight and gestational age in neonatal mortality risk evaluation. *Rev Saude Publica.*2003;37(5):597–602.

53.de Mello FB, de Almeida MFB, dos Santos AMN, Costa H de PF, Miyoshi MH, Amaro ER. Factors associated with survival of very-low-birth-weight infants in a Brazilian fee-paying maternity in the 1990s. *J Trop Pediatr*. 2007;53(3):153–7.

54.Chioukh FZ, Ben Ameur K, Kasdallah N, Blibech S, Ben Hamida E, Ayadi I, et al. Extremely preterm infants in Tunisia: Where are we now? *Tunis Med.* 2018;96(8-9):510-513.

55.Gebeşçe A, Uslu H, Keleş E, Yildirim A, Gürler B, Yazgan H, et al. Retinopathy of prematurity: Incidence, risk factors, and evaluation of screening criteria.*Turkish J Med Sci* 2016;46(2):315–20.

56.Gezmu AM, Shifa JZ, Quinn GE, Nkomazana O, Ngubula JC, Joel D, et al. Incidence of retinopathy of prematurity in botswana: A prospective observational study. *Clin Ophthalmol.* 2020;14:2417–25.

57.Gharaibeh A, Khassawneh M, Khriesat W, Alkhatib S, Migdadi Y. Adopting western retinopathy of prematurity screening programs in eastern countries, are we screening properly. *Middle East Afr J Ophthalmol*. 2011;18(3):209–13.

58.Ghaseminejad A, Niknafs P. Distribution of retinopathy of prematurity and its risk factors. *Iran J Pediatr*. 2011;21(2):209–14.

59.Golestan M, Fallah R, Karbasi SA. Neonatal mortality of low birth weight infants in Yazd, Iran. *Iran J Reprod Med*. 2008;6(4):205–8.

60.Gonçalves E, Násser LS, Martelli DR, Alkmim IR, Mourão TV, Caldeira AP, et al. Incidence and risk factors for retinopathy of prematurity in a Brazilian reference service. *São Paulo Med J.* 2014;132 (2): 85-9

61.Gooden M, Younger N, Trotman H. What is the best predictor of mortality in a very low birth weight infant population with a high mortality rate in a medical setting with limited resources? *Am J Perinatol*. 2014;31(6):441–6.

62.Goulart AL, de Morais MB, Kopelman BI. Impact of perinatal factors on growth deficits in preterm infants. *Rev Assoc Med Bras*. 2011;57(3):269–75.

63.Grupo Colaborative Neocosur. Very-Low-Borth-Weight Infant Outcomes in 11 South American NICUs. *J Perinatol*. 2002;22:2–7.

64.Gupta S, Adhisivam B, Bhat BV, Plakkal N, Amala R. Short Term Outcome and Predictors of Mortality Among Very Low Birth Weight Infants – A Descriptive Study. *Indian J Pediatr*. 2020.

65.Hadi AM, Hamdy IS. Correlation between risk factors during the neonatal period and appearance of retinopathy of prematurity in preterm infants in neonatal intensive care units in Alexandria, Egypt. *Clin Ophthalmol*. 2013;7:831-837.

66.Gutiérrez Saucedo ME, Hernández Herrera RJ, Luna García SA, Flores Santos R, Alcalá Galván LG, Martínez Gaytán V. Perinatal mortality at Hospital de Ginecoobstetricia No. 23 of Monterrey, Nuevo León, 2002-2006 period. *Ginecol Obstet Mex*. 2008;76(5):243-248.

67.Haghighi L, Nojomi M, Mohabbatian B, Najmi Z. Survival predictors of preterm neonates: Hospital based study in Iran (2010-2011). *Iran J Reprod Med*. 2013;11(12):957–64.

68.Hakeem AA, Mohamed G, Othman M. Retinopathy of prematurity: A study of incidence and risk factors in NICU of Al-Minya University Hospital in Egypt. *J Clin Neonatol.* 2012;1:76.

69.Hendriks H, Kirsten GF, Voss M, Conradie H. Is continuous positive airway pressure a feasible treatment modality for neonates with respiratory distress syndrome in a rural district hospital? *J Trop Pediatr*. 2014;60(5):348–51.

70.Ho JJ. Late onset infection in very low birth weight infants in Malaysian Level 3 neonatal nurseries. *Pediatr Infect Dis J*. 2001;20(6):557–60.

71.Hussain, M. The Value of Surfactant Therapy in Preterm Neonates with Respiratory Distress Syndrome. *Indian Journal of Public Health Research & Development.*2020;11(2): 2137-2141.

72.Jiang S, Yan W, Li S, Zhang L, Zhang Y, Shah PS, et al. Mortality and Morbidity in Infants <34 Weeks' Gestation in 25 NICUs in China: A Prospective Cohort Study. *Front Pediatr*. 2020;8:33.

73.Jodeiry B, Heidarzadeh M, Sahmani-Asl S, Hoseini M, Javaherizadeh H, Eliasi S, et al. Study of intraventricular hemorrhage in VLBW neonates admitted in Al-Zahra Hospital, Tabriz, Iran. *Niger J Med*. 2012;21(1):92-97.

74.Jirapaet K, Jirapaet V, Sritipsukho S. Safety of initiating early enteral feeding with slow volume advancement in preterm infants. *J Med Assoc Thail*. 2010;93(10):1177–87.

75.Kalimba E,Ballot D. Survival of extremely low-birth-weight infants. *South African Journal Of Child Health.*2013;*7*(1):13-16.

76.Karabulut B, Paytoncu S. Efficacy and Safety of Oral Paracetamol vs. Oral Ibuprofen in the Treatment of Symptomatic Patent Ductus Arteriosus in Premature Infants. *Pediatr Drugs.* 2019;21(2):113–21.

77.Karkhaneh R, Mousavi SZ, Riazi-Esfahani M, Ebrahimzadeh SA, Roohipoor R, Kadivar M, et al. Incidence and risk factors of retinopathy of prematurity in a tertiary eye hospital in Tehran. *Br J Ophthalmol*. 2008;92(11):1446–9.

78.Kirsten GF, Kirsten CL, Henning PA, Smith J, Holgate SL, Bekker A, et al. The outcome of ELBW infants treated with NCPAP and inSurE in a resource-limited institution.*Pediatrics*. 2012;129(4).

79.Moundzika-Kibamba JC, Nakwa FL. Neonatal mortality at Leratong hospital. *SAJCH South African J Child Heal.* 2018;12:24–8.

80.Klingenberg C, Olomi R, Oneko M, Sam N, Langeland N. Neonatal morbidity and mortality in a Tanzanian tertiary care referral hospital. *Ann Trop Paediatr*. 2003;23(4):293–9.

81.Kong XY, Xu FD, Wu R, Wu H, Ju R, Zhao XL, et al. Neonatal mortality and morbidity among infants between 24 to 31 complete weeks: A multicenter survey in China from 2013 to 2014. *BMC Pediatr*. 2016;16(1):1–8.

82.Kong X, Xu F, Wang Z, Zhang S, Feng Z. Antenatal corticosteroids administration on mortality and morbidity in premature twins born at 25~34 gestational weeks: A retrospective multicenter study. *Eur J Obstet Gynecol Reprod Biol*. 2020;253:259–65.

83.Köksal N, Baytan B, Bayram Y, Nacarküçük E. Risk factors for intraventricular haemorrhage in very low birth weight infants. *Indian J Pediatr*. 2002;69(7):561-564.

84.Kulali F, Bas AY, Erol S, Yucel H, Guzel FI, Yalvac S, et al. Survival of periviable infants: 5-year experience at a single center. *J Matern Neonatal Med*. 2020;33(22):3725–31.

85.Lara-Molina NC, Ramírez-Ortiz MA, Ríos-Medina S, Karol J. Méndez-Ochoa, María T, Tinoco-Zamudio et al. State screening program for retinopathy of prematurity in the state of Michoacán. *Gac Med Mex*. 2013; 149 (3): 280-285.

86.Lermann VL, Borges Fortes Filho J, Procianoy RS. The prevalence of retinopathy of prematurity in very low birth weight newborn infants. *J Pediatr (Rio J)*. 2006;82(1):27–32.

87. GuangXi Cooperative Research Group for Extremely Preterm Infants., Li Y, Meng DH, Wei QF, Pan XN, Liang WH, Huang HY, Zhen H, Zhang SY, Wei Y, Wu CB, Wei YC, Zhou JX, Lu GX. Neurodevelopmental outcomes of extremely preterm infants in southern China: A multicenter study. Early Hum Dev. 2019 Jun;133:5-10

88.Li Y, Wei QF, Meng DH, Pan XN, Mo Y, Yao LP et al. Treatment outcomes and associated factors among extremely preterm infants in a major children hospital in Guangxi, China. *Pediatr Neonatol*. 2018;59(3):263–6.

89.Lin HJ, Du LZ, Ma XL, Shi LP, Pan JH, Tong XM, et al. Mortality and morbidity of extremely low birth weight infants in the mainland of china: A multi-center study. *Chin Med J* (Engl). 2015;128(20):2743–50.

90.Liu PM, Fang PC, Huang C Bin, Kou HK, Chung MY, Yang YH et al. Risk factors of retinopathy of prematurity in premature infants weighing less than 1600 g. *Am J Perinatol.* 2005;22(2):115–20.

91.Liu Q, Yin ZQ, Ke N, Chen L, Chen XK, Fang J, et al. Incidence of retinopathy of prematurity in Southwestern China and analysis of risk factors.*Med Sci Monit*. 2014;20:1442–51.

92.Lomuto CC, Galina L, Brussa M, Quiroga A, Alda E, Beníteza AM, et al. Epidemiology of retinopathy of prematurity in publics services from Argentina during 2008. *Arch Argent Pediatr.* 2010;108(1):24–30.

93.Luthuli NP, McKerrow NH. Short-term outcomes of infants with an extremely low birth weight in a resource-limited neonatal intensive care unit, grey's hospital, kwazulu-natal. *South African J Child Heal*. 2019;13(3):120–4.

94.Mabhandi T, Ramdin T, Ballot DE. Growth of extremely low birth weight infants at a tertiary hospital in a middle-income country.*BMC Pediatr*. 2019;19(1):1–9.

95.Padilla Martínez YM, Hernández Herrera RJ. Conditions associated with mortality in neonates weighing < 1000 g at the Obstetric-Gynecology Hospital, Unit N° 23, Monterrey, Mexico (2005-2006). *Bol Med Hosp Infant Mex*. 2010;67:335-343.

96.Martínez-Cruz CF, Salgado-Valladares M, Poblano A, Trinidad-Pérez MC. Risk factors associated with retinopathy of prematurity and visual alterations in infants with extremely low birth weight. *Rev Investig Clin*. 2012;64(2):136–43.

97.McGready R, Paw MK, Wiladphaingern J, Min AM, Carrara VI, Moore KA et al. The overlap between miscarriage and extreme preterm birth in a limited-resource setting on the thailand-myanmar border: A population cohort study. *Wellcome Open Res*. 2018;1:1–23.

98.Medina-Valentón E, Salgado-López DG, López-Morales CM.Retinopathy of prematurity in a second level hospital in Mexico. *Rev Mex Pediatr*. 2016; 83 (3): 80-84.

99.Mekasha A, Tazu Z, Muhe L, Abayneh M, Gebreyesus G, Girma A et al. Factors Associated with the Death of Preterm Babies Admitted to Neonatal Intensive Care Units in Ethiopia: A Prospective, Cross-sectional, and Observational Study. *Glob Pediatr Heal*. 2020;7.

100.Miles M, Dung KTK, Ha LT, Liem NT, Ha K, Hunt RW et al. The cause-specific morbidity and mortality, and referral patterns of all neonates admitted to a tertiary referral hospital in the northern provinces of Vietnam over a one year period. *PLoS One*. 2017;12(3):1–12.

101.Moghaddam PS, Aghaali M. Survival of 798 low birth weight infants according to birth weight and gestational age. *Shiraz E Med J*. 2015;16(10).

102.Montaño-Pérez CM, Cázarez-Ortiz M, Juárez-Astorga A, Ramírez-Moreno MA. Morbidity and mortality in newborns under 1,000 grams in a third level public institution in Mexico. *Rev Mex Pediatr.* 2019; 86 (3):108-111.

103.Moundzika-Kibamba JC, Nakwa FL. Neonatal mortality at Leratong hospital. *South African J Child Heal.* 2018;12(1):24–8.

104.Muhe LM, McClure EM, Nigussie AK, Mekasha A, Worku B, Worku A et al. Major causes of death in preterm infants in selected hospitals in Ethiopia (SIP): a prospective, cross-sectional, observational study. *Lancet Glob Heal*. 2019;7(8):e1130–8.

105.Mukhopadhyay K, Louis D, Murki S, Mahajan R, Dogra MR, Kumar P. Survival and morbidity among two cohorts of extremely low birth weight neonates from a tertiary hospital in Northern India. *Indian Pediatr*. 2013;50(11):1047–50.

106.Nakubulwa C, Musiime V, Namiiro FB, Tumwine JK, Hongella C, Nyonyintono J et al. Delayed initiation of enteral feeds is associated with postnatal growth failure among preterm infants managed at a rural hospital in Uganda. *BMC Pediatr*. 2020;20(1):1–9.

107.Navaei F, Aliabady B, Moghtaderi J, Moghtaderi M, Kelishadi R. Early outcome of preterm infants with birth weight of 1500 g or less and gestational age of 30 weeks or less in Isfahan city, Iran. *World J Pediatr*.2010;6(3):228–32.

108.Nepal D, Agrawal S, Shrestha S, Rayamajhi A. Morbidity pattern and hospital outcome of neonates admitted in Tertiary Care Hospital, Nepal. *J Nepal Paediatr Soc*. 2020;40(2):107–13.

109.Nevačinović E, Cerovac A, Bogdanović G, Cerovac E, Tupek T, Zukić H. Perinatal Mortality According to Level of Perinatal Healthcare Institutions in Low Birth Weight Infants: Cross Sectional Multicentric Study. *Int J Prev Med*. 2020;11:72.

110.Ntuli TS, Mashego MPA, Shipalana N, Sutton C, Hamese MHK. Factors associated with preterm very low birthweight infant mortality at a tertiary hospital in Limpopo province, South Africa. *South African J Child Heal*. 2020;14(1):10–4.

111.Obaid KA, Alazzawi DSH. Outcome of low birth weight infants in Diyala province of Iraq. *J Trop Pediatr*. 2011;57(4):280–2.

112.Ogunlesi TA. Factors influencing the survival of newborn babies weighing <1.5 kg in Sagamu, Nigeria. *Arch Gynecol Obstet*. 2011;284(6):1351–7.

113.Okello F, Egiru E, Ikiror J, Acom L, Loe KSM, Olupot-Olupot P et al. Reducing preterm mortality in eastern Uganda: The impact of introducing low-cost bubble CPAP on neonates <1500 g. *BMC Pediatr*. 2019;19(1):1–7.

114.Omer IM, Hassan HA. The prevalence and risk factors of retinopathy of prematurity among preterm babies admitted to Soba Neonatal Intensive Care Unit. *Sudan J Paediatr* . 2014;14(2):17–21.

115.Omoigberale AI, Sadoh WE, Nwaneri DU. A 4 year review of neonatal outcome at the University of Benin Teaching Hospital, Benin City. *Niger J Clin Pract*. 2010;13(3):321–5.

116.Onalo R, Olateju EK. A Four-Year Retrospective Review of Very Low Birth Weight Babies Seen at the University of Abuja Teaching Hospital, Abuja, Nigeria. *Niger Postgrad Med J* 2015;22(2):93–9.

117.Onyiriuka AN. Incidence of delivery of low birthweight infants in twin estations. *Niger J Clin Pract*. 2010;13(4):365–70.

118.Oommen SP, Santhanam S, John H, Roshan R, Swathi TO, Padankatti C et al. Neurodevelopmental Outcomes of Very Low Birth Weight Infants at 18-24 Months, Corrected Gestational Age in a Tertiary Health Centre: A Prospective Cohort Study. *J Trop Pediatr.* 2019 Dec 1;65(6):552-560..

119.Osorno-covarrubias L, Neonat P, Vela-urtecho G, Ped M. Graphic representation of the neonatal mortality risk at a regional perinatal center in Mérida, Yucatán, México. *Salud pública de México.* 2002;44:345-8.

120.Özcan P, Con R, Çelik HT. Incidence, Risk Factors and Treatment Outcomes of Retinopathy of Prematurity in the Southeastern Anatolian Region Province of Şanlıurfa in Turkey. *Turkiye Klin J Med Sci*. 2015;35:240–7.

121.Qazi G. Obstetric and perinatal outcome of multiple pregnancy. *J Coll Physicians Surg Pak*. 2011;21(3):142-145.

122.Paul VK, Jain S, Jain P, Verma M, Chacko B, Dani VS et al. Morbidity and mortality among outborn neonates at 10 tertiary care institutions in India during the year 2000. *J Trop Pediatr*. 2004;50(3):170–4.

123.Pervin J, Gustafsson FE, Moran AC, Roy S, Persson LA, Rahman A. Implementing Kangaroo mother care in a resource-limited setting in rural Bangladesh. *Acta Paediatr*. 2015;104(5):458-465.

124.Piriyapokin N, Chuthapisith J, Emrat K, Nuntnarumit P. Outcomes of preterm infants born with marginal viability in a University Hospital in Thailand. *J Paediatr Child Health*. 2020 Jun;56(6): 943-949

125.Poudel P, Budhathoki S. Perinatal characteristics and outcome of VLBW infants at NICU of a developing country: an experience at eastern Nepal. *J Matern Fetal Neonatal Med*. 2010;23(5): 441-447.

126.Pourarian S, Farahbakhsh N, Sharma D, Cheriki S, Bijanzadeh F. Prevalence and risk factors associated with the patency of ductus arteriosus in premature neonates: a prospective observational study from Iran. *J Matern Neonatal Med*. 2017;30(12):1460–4.

127.Prabha PCN, George RT, Francis F. Profile and outcome of neonates requiring ventilation: The Kerala experience. *Curr Pediatr Res*. 2014;18(2):57–62.

128.Pradhan D, Nishizawa Y, Chhetri HP. Prevalence and outcome of preterm births in the National Referral Hospital in Bhutan: An observational study. *J Trop Pediatr*. 2020;66(2):163-70.

129.Qian L, Liu C, Zhuang W, Guo Y, Yu J, Chen H et al. Neonatal respiratory failure: a 12-month clinical epidemiologic study from 2004 to 2005 in China. *Pediatrics*. 2008;121(5):e1115-e1124.

130.Rezaeizadeh G, Dalili H, Shariat M, Fallahi M, Other Members Of Maternal Group O, Nayeri F. The Iranian Neonatal Registry: Primary Results. *Arch Iran Med.* 2018 Apr 1;21(4):145-152.

131.Roy KK, Baruah J, Kumar S, Malhotra N, Deorari AK, Sharma JB. Maternal antenatal profile and immediate neonatal outcome in VLBW and ELBW babies. *Indian J Pediatr.* 2006;73(8):669–73.

132.Ruiz-Peláez JG, Charpak N. Bronchopulmonary dysplasia epidemic: incidence and associated factors in a cohort of premature infants in Bogotá, Colombia.*Biomedica*. 2014;34(1):29–39.

133.Rylance S, Ward J. Early mortality of very low-birthweight infants at Queen Elizabeth Central Hospital, Malawi. *Paediatr Int Child Health*. 2013;33(2):91–6.

134.Sabzehei MK, Afjeh SA, Farahani AD, Shamshiri AR, Esmaili F. Retinopathy of prematurity: Incidence, risk factors, and outcome. *Arch Iran Med*. 2013;16(9):507–12.

135.Sackey AH, Tagoe LG. Admissions and mortality over a 5-year period in a limited-resource neonatal unit in Ghana. *Ghana Med J*. 2019;53(2):117–25.

136.Saeidi R, Hashemzadeh A, Ahmadi S, Rahmani S. Prevalence and predisposing factors of retinopathy of prematurity in very low-birth-weight infants discharged from NICU. *Iran J Pediatr*. 2009;19(1):59–63.

137.Saeidi R, Taraghi B, Saeidi M. Incidence of retinopathy of prematurity (ROP) in low birth wight newborns. *Iran J Neonatol*. 2017;8(4):102–6.

138.Şahin A, Şahin M, Türkcü FM, Cingü AK, Yüksel H, Çınar Y et al. Incidence of Retinopathy of Prematurity in Extremely Premature Infants. *ISRN Pediatr*. 2014: 2014:4.

139.Sahoo T, Anand P, Verma A, Saksena M, Sankar MJ, Thukral A et al. Outcome of extremely low birth weight (ELBW) infants from a birth cohort (2013–2018) in a tertiary care unit in North India. *J Perinatol*. 2020;40(5):743–9.

140.Saini N, Chhabra S, Chhabra S, Garg L, Garg N. Pattern of neonatal morbidity and mortality: A prospective study in a District Hospital in Urban India. *J Clin Neonatol*. 2016;5(3):183.

141.Salahuddin A, Jan AZ, Zahid SB, Aleem M. To share our experience of invasive ventilation in NICU at Rehman Medical Institute, Peshawar-Pakistan. *Med Forum Mon*. 2018;29(7):40–3.

142.Saygili O, Özcan E, Kimyon S, Mete A, Kenan S, Güngör K et al. Retinopathy of prematurity screening in a tertiary hospital in the south-east region of Turkey: Prevalence and relation with multiple pregnancies. *Retina-Vitreus*. 2017;26(2):105–9.

143.Sehgal A, Telang S, Passah SM, Jyothi MC. Maternal and Neonatal Profile and Immediate Outcome in Extremely Low Birth Weight Babies in Delhi. *Trop Doct*. 2004;34(3):165–8.

144.Seid SS, Ibro SA, Ahmed AA, Olani Akuma A, Reta EY, Haso TK et al.Causes and factors associated with neonatal mortality in Neonatal Intensive Care Unit (NICU) of Jimma University Medical Center, Jimma, South West Ethiopia. *Pediatric Health Med Ther*. 2019;10:39-48.

145. Serçe O, Similar D , Gürsoy T, Ovalı , Karatekin G.Third Line Intensive Care of a Reference Hospital in Istanbul Clinical Prognosis of Newborns with Very Low Birth Weight Followed in the Unit. *Zeynep Kamil Medical Bulletin*.2020;370:1–9.

146.Shrestha JB, Bajimaya S, Sharma A, Shresthal J, Karmacharya P. Incidence of retinopathy of prematurity in a neonatal intensive care unit in Nepal. *J Pediatr Ophthalmol Strabismus.* 2010;47(5):297–300.

147.Singh J, Dalal P, Gathwala G. Clinical profile and predictors of mortality among the referred neonates at a tertiary care centre in north India: a prospective observational study. *Trop Doct.* 2020;50(3):221–7.

148.Siswanto JE, Widodo NH, Sauer PJJ. Eleven years of retinopathy of prematurity in one neonatal intensive care unit in Jakarta, Indonesia. *Arch Dis Child.* 2018;103(6):619–21.

149.Sivanandan S, Chandra P, Deorari AK, Agarwal R. Retinopathy of Prematurity: AIIMS, New Delhi Experience. *Indian Pediatr*. 2016;53(November):S123–8.

150.Sousa DS, Sousa Júnior AS, Santos ADR, Melo EV, Lima SO, Almeida-Santos MA et al. Morbidity in extreme low birth weight newborns hospitalized in a high risk public maternity. *Rev Bras Saúde Matern Infant*. 2017;17(1):139–47.

151.Sritipsukho S, Suarod T, Sritipsukho P. Survival and outcome of very low birth weight infants born in a university hospital with level II NICU. *J Med Assoc Thail*. 2007;90(7):1323–9.

152.Sun L, Yue H, Sun B, Han L, Qi M, Tian Z, et al. Estimation of birth population-based perinatal-neonatal mortality and preterm rate in China from a regional survey in 2010. *J Matern Fetal Neonatal Med.* 2013;26(16):1641–8.

153.Tamene A, Abeje G, Addis Z. Survival and associated factors of mortality of preterm neonates admitted to Felege Hiwot specialized hospital, Bahir Dar, Ethiopia. *SAGE Open Med*. 2020;8:205031212095364.

154.Taqui AM, Syed R, Chaudhry TA, Ahmad K, Salat MS. Retinopathy of prematurity: Frequency and risk factors in a tertiary care hospital in Karachi, Pakistan. *J Pak Med Assoc*. 2008;58(4):186–90.

155.Tavosnanska J, Carreras IM, Fariña D, Luchtenberg G, Celadilla ML, Celotto M et al. Mortality and morbidity of very low birth weight newborn infants assisted in Buenos Aires public hospitals. *Arch Argent Pediatr*. 2012 Oct;110(5):394-403.

156.Thakre S, Deshmukh P, Kalyanshetti G, Mishrikotkar J. Incidence, severity, and risk factors of retinopathy of prematurity in central Maharashtra, India. *Perinatology*. 2017;18(2):50–5.

157.Thakur N, Saili A, Kumar A, Kumar V. Predictors of mortality of extremely low birthweight babies in a tertiary care centre of a developing country. *Postgrad Med J*. 2013;89(1058):679–84.

158.Tosif S, Jatobatu A, Maepioh A, Subhi R, Francis KL, Duke T. Cause-specific neonatal morbidity and mortality in the Solomon Islands: An assessment of data from four hospitals over a three-year period. *J Paediatr Child Health*. 2020;56(4):607–14.

159.Tran HT, Doyle LW, Lee KJ, Dang NM, Graham SM. Morbidity and mortality in hospitalised neonates in central Vietnam. *Acta Paediatr Int J Paediatr*. 2015;104(5):e200–5.

160.Trotman H. Review of mortality of very low birthweight infants at the University Hospital of the West Indies over the past four decades. *West Indian Med J*. 2012;61(4):356–60.

161.Trotman H, Barton M, Mitchell V. Outcome of neonates ventilated in the main intensive care unit at The University Hospital of the West Indies: A 15-year experience. *Trop Doct*. 2007;37(4): 249–50.

162.Trotman H, Bell Y. Neonatal sepsis in very low birthweight infants at the University Hospital of the West Indies. *West Indian Med J*. 2006;55(3):165–9.

163.Trotman H, Lord C. Outcome of extremely low birthweight infants at the University Hospital of the West Indies, Jamaica. *West Indian Med J.* 2007;56(5):409–13.

164.Tshehla RM, Coetzee M, Becker PJ. Mortality and morbidity of very low-birthweight and extremely low-birthweight infants in a tertiary hospital in Tshwane. *South African J Child Heal*. 2019;13(2):89–97.

165.Ugwu R, Eneh A. The Proportion Of Low Birth Weight Babies Due To Small For Gestational Age (Sga) And Prematurity In Port Harcourt, South-South Nigeria - Changing Trends*. The Internet Journal of Pediatrics and Neonatology.*2010;13(1):1–6.

166.Undela K, Mohammed BTS, Gurumurthy P, Doreswamy SM. Impact of preterm birth and low birth weight on medical conditions, medication use and mortality among neonates: a prospective observational cohort study. *World J Pediatr*. 2019;15(3):281–8.

167.Cauich-Aragón LM, De la Fuente-Torres MA, Sánchez-Buenfil E, Farías-Cid R. Epidemiological characterization of retinopathy of prematurity at the Korea-Mexico Friendship Hospital. Period 2005 to 2014. *Perinatol and Reprod Humana.* 2017; 31: 21–7.

168.Velaphi SC, Mokhachane M, Mphahlele RM, Beckh-Arnold E, Kuwanda ML, Cooper PA. Survival of very-low-birth-weight infants according to birth weight and gestational age in a public hospital. *South African Med J*. 2005;95(7):504–9.

169.Viau ÂC, Kawakami MD, Teixeira MLP, Waldvogel BC, Guinsburg R, De Almeida MFB. First-and fifth-minute Apgar scores of 0-3 and infant mortality: A population-based study in Sao Paulo State of Brazil. *J Perinat Med*. 2015;43(5):619–25.

170.Vilanova CS, Hirakata VN, De Souza Buriol VC, Nunes M, Goldani MZ, Da Silva CH. The relationship between the different low birth weight strata of newborns with infant mortality and the influence of the main health determinants in the extreme south of brazil. *Popul Health Metr.* 2019;17(1):1–13.

171.Vural M, Yilmaz I, Ilikkan B, Erginoz E, Perk Y. Intraventricular hemorrhage in preterm newborns: Risk factors and results from a University Hospital in Istanbul, 8 years after. *Pediatr Int.* 2007;49(3):341–4.

172.Wang H, Gao X, Liu C, Yan C, Lin X, Yang C et al. Morbidity and mortality of neonatal respiratory failure in China: Surfactant treatment in very immature infants. *Pediatrics*. 2012;129(3):e731-e740.

173.Welbeck J, Biritwum RB, Mensah G. Factors affecting the survival of the Òat riskÓ newborn at Korle Bu Teaching Hospital, Accra, Ghana. *West Afr J Med*. 2003;22(1):55–8.

174.Winkler LA, Stypulkowski A, Noon S, Babwanga T, Lutahoire J. A multi-year analysis of kangaroo mother care outcomes in low birth weight babies at a nyakahanga hospital in rural Tanzania. *Afr Health Sci*. 2020;20(1):498–508.

175.Wu F, Liu G, Feng Z, Tan X, Yang C, Ye X et al. Short-term outcomes of extremely preterm infants at discharge: A multicenter study from Guangdong province during 2008-2017. *BMC Pediatr.* 2019;19(1):1–11.

176.Wu T, Zhang L, Tong Y, Qu Y, Xia B, Mu D. Retinopathy of prematurity among very low-birth-weight infants in china: Incidence and perinatal risk factors. *Investig Ophthalmol Vis Sci*. 2018;59(2):757–63.

177.Xu F, Kong X, Duan S, Lv H, Ju R, Li Z et al. Care Practices, Morbidity and Mortality of Preterm Neonates in China, 2013–2014: a Retrospective study. *Sci Rep* 2019;9(1):2013–4.

178.Xu Y, Zhou X, Zhang Q, Ji X, Zhang Q, Zhu J et al. Screening for retinopathy of prematurity in China: a neonatal units-based prospective study. *Invest Ophthalmol Vis Sci*. 2013;54(13):8229–36.

179.Yadav SK, Giri A. Safety of early rescue surfactant replacement therapy for preterm neonates with respiratory distress syndrome at neonatal intensive care unit of a tertiary hospital. *J Nepal Paediatr Soc.* 2019;39(3):162–7.

180.Yakoob MY. Characteristics of very preterm infants delivered at a tertiary care center in Pakistan. *Mymensingh Med J.* 2014;23(3):558–62.

181.Yau GSK, Lee JWY, Tam VTY, Liu CCL, Chu BCY, Yuen CYF. Incidence and risk factors for retinopathy of prematurity in extreme low birth weight Chinese infants. *Int Ophthalmol*. 2015;35(3):365–73.

182.Zea-Vera A, Turín CG, Rueda MS, Guillén-Pinto D, Medina-Alva P, Tori A et al. Intraventricular hemorrhage and periventricular leukomalacia in low birth-weight neonates in three hospitals in Lima, Peru. *Rev Peru Med Exp Salud Publica*. 2019;36(3):448–53.

183.Zepeda-Romero LC, Lundgren P, Gutierrez-Padilla JA, Gomez-Ruiz LM, Corona MQ, Orozco-Monroy JV et al. Oxygen Monitoring Reduces the Risk for Retinopathy of Prematurity in a Mexican Population. *Neonatology.* 2016;110(2):135–40.

184.Zhang H, Fang J, Su H, Chen M. Risk factors for bronchopulmonary dysplasia in neonates born at 1500 g (1999-2009). *Pediatr Int*. 2011;53(6):915–20.

185.Zhang J, Liu GH, Zhao YW, Wang HQ, Mao SG, Mao GS et al. A multicenter epidemiological investigation of brain injury in hospitalized preterm infants in Anhui, China. *Chinese J Contemp Pediatr.* 2019;21(2):114–9.

186.Zhang L, Qiu Y, Yi B, Ni L, Zhang L, Taxi P, et al. Mortality of neonatal respiratory failure from Chinese northwest NICU network. *J Matern Neonatal Med*. 2017;30(17):2105–11.

187.Zhang T, Chen J, Wu H, Pan W, Yang X, Li Y, Liu M, Huang Y. Improved survival and survival without bronchopulmonary dysplasia in very low birth weight infants after active perinatal care. *Niger J Clin Pract.* 2020 Jul;23(7):980-987.

188.Zhou WQ, Mei YB, Zhang XY, Li QP, Kong XY, Feng ZC. Neonatal outcomes of very preterm infants from a neonatal intensive care center. *World J Pediatr*. 2014;10(1):53–8.

189.Zhu Z, Wang J, Chen C, Zhou J. Hospitalization charges for extremely preterm infants: a ten-year analysis in Shanghai, China. *J Med Econ*. 2020;1-8.

190.Ziadeh S. Outcome of twin pregnancies in North Jordan. *J Obstet Gynaecol*. 2000;20(5): 492-494.

191.Ziylan S, Yabas Ö, Zorlutuna N, Daruga I. Retinopathy of Prematurity in Babies with a Birth Weight of 1000gr or Less. *Turk J Ophthalmol* 2006;36(5):406-410.

192.Zuniga I, Van den Bergh R, Ndelema B, Bulckaert D, Manzi M, Lambert V et al. Characteristics and mortality of neonates in an emergency obstetric and neonatal care facility, rural Burundi. *Public Heal Action*. 2013;3(4):276–81.
